# Hydration-dependent exciton-phonon coupling in a metal-organic framework photocatalyst

**DOI:** 10.1038/s41467-026-76097-z

**Published:** 2026-08-05

**Authors:** Beatriz Mouriño, Antonios Alvertis, Alex Smith, Nency P. Domingues, Fatmah Mish Ebrahim, Aurélie Champagne, Jeffrey B. Neaton, Berend Smit

**Affiliations:** 1 Laboratory of Molecular Simulation (LSMO), Institut des Sciences et Ingénierie Chimiques, Ecole Polytechnique Fédérale de Lausanne (EPFL), Sion, Switzerland; 2 Materials Sciences Division, Lawrence Berkeley National Laboratory, Berkeley, CA USA; 3 Department of Physics, The University of Texas at Austin, Austin, TX USA; 4 Oden Institute for Computational Engineering and Sciences, The University of Texas at Austin, Austin, TX USA; 5 Department of Physics, University of California, Berkeley, Berkeley, CA USA; 6 Université de Bordeaux, CNRS, Bordeaux INP, ICMCB, UMR 5026, Pessac, France; 7 Kavli Energy Nanosciences Institute at Berkeley, Berkeley, CA USA; 8 Present Address: Department of Chemistry, Columbia University, New York, New York, NY USA

**Keywords:** Theory and computation, Materials for energy and catalysis, Metal-organic frameworks, Electronic properties and materials, Electronic structure

## Abstract

Metal-organic frameworks (MOFs) have potential for light-harvesting applications owing to their tunable optoelectronic properties via building block selection. The presence of bound solvent in MOFs is known to impact gas adsorption properties, but little is known about their effect on optical properties. Here, we investigate how the presence of bound water can modulate the optical properties of a reported MOF photocatalyst, SU-101. Using the GW approximation and the ab initio Bethe-Salpeter equation (BSE) and treating exciton-phonon coupling with a finite difference method, we show that exciton-phonon interactions in SU-101 are strong and highly sensitive to the presence of bound water molecules. Specifically, we find that the presence of bound water is associated with a stronger localization of excitons due to phonons. Guided by our calculations, we synthesize SU-101 and study this MOF under different water loadings. We measure photoluminescence (PL) emission and UV-Vis absorption spectra of the MOFs, the latter in good agreement with our computational predictions. By considering the interplay of water loading, phonons, and excitons, our calculations rationalize measured UV-Vis absorption and PL spectra. Our work also highlights the pivotal role of exciton-phonon interactions and their sensitivity to hydration on the optoelectronic properties of MOFs with large pores and flexible organic linkers.

## Abstract (web summary)

Here the authors combine experiment and theory to show that bound water affects exciton-phonon coupling in SU-101. The results help explain hydration-dependent UV-Vis and PL spectra, and show that excitons and phonons can play an important role in MOFs.

## Introduction

Metal-organic frameworks (MOFs) are porous modular materials composed of organic linkers and metal nodes connected in various underlying nets^1,2^. The highly tunable nature of MOFs makes them interesting for a wide variety of applications^3–5^. For light-harvesting applications, in particular, the combined metallic and organic nature of MOFs can be synergistic^6^. Light-absorbing organic linkers can shift the optical gap into the commercially relevant visible range^7^, while metal nodes can act as electron acceptors upon excitation. For photocatalysis, this modular design could potentially enhance charge separation upon light absorption, thus improving photocatalytic performance^8^. In view of these promising characteristics, recent studies have increasingly explored MOFs as photocatalysts from both experimental and computational perspectives^7,9–14^.

In MOFs, the metal sites are mostly coordinated by the surrounding organic linkers through reversible bonds, such as metal-carboxylate bonds, which influence the stability of these materials^15,16^. Additionally, the metal sites in MOFs can be coordinated by bound solvents^17^, i.e., water (hydration). Because metal-solvent bonds are typically more labile than metal-linker bonds, the solvent can usually be removed without collapsing the framework^17^. The removal of bound solvents upon activation leads to coordinatively unsaturated metal sites that can act as catalytic hotspots (e.g., Lewis acid sites) and/or enhance the gas adsorption properties of a MOF^17–19^. Although the effect of bound solvents on catalysis and gas adsorption is widely established^17–19^, fewer studies focused on how bound solvents can affect the optical properties of a MOF^19–21^, and therefore its light-harvesting feasibility.

The optical properties of MOFs are largely influenced by their porous and hybrid organic-inorganic nature. Prototypical bulk all-inorganic semiconductors usually display sufficient dielectric screening to lead to weakly bound excited electron-hole pairs (excitons)^22^, and some phonon modulation of their optoelectronic properties^23^. On the other hand, classic organic semiconductors tend to have more strongly bound excitons with binding energies on the order of 0.5 eV to 1 eV^24^, with phonons reported to have significant effects (~0.5 eV) on fundamental gaps^25,26^ and on excitonic properties^27,28^. For MOFs, due to their hybrid organic-inorganic nature and inherent porosity, studies report relatively large exciton binding energies^29,30^ and significant phonon effects^31,32^. The strong excitonic effects and electron-phonon coupling (EPC) in MOFs stem from electronic excitations localized on the organic linkers and are similar to those reported in organic solids, which can exhibit strong exciton-phonon coupling leading to localization^27^. As with organic solids and other molecular systems, displacements of atoms along high-frequency intramolecular coordinates drive this strong coupling^33^, and soft degrees of freedom give rise to the temperature dependence^27^ and even self-trapping^34^. In inorganic semiconductors, Wannier–Mott excitons are delocalized over many unit cells and couple predominantly to polar optical phonon modes^35,36^, a mechanism that is essentially absent when the exciton is sub-unit-cell in extent in a less-polar MOF. Nevertheless, the nature of excitons in MOFs, how strongly they interact with phonons, and whether this interaction is in any way hydration-dependent remain largely unexplored.

In this work, we investigate the role of bound water and exciton-phonon coupling in SU-101^37^, a reported photocatalyst with a Tauc-derived optical gap of 2.6 eV^14,38^. SU-101 is composed of ellagic acid-derived linkers and one-dimensional Bi-oxo clusters^37^. Bi sites in SU-101 are additionally coordinated by water, which can be removed after activation to expose Lewis acid sites^39^. Moreover, experimental evidence of light-induced H-bond breaking suggests strong exciton-phonon coupling in SU-101^40^. Using a finite-displacement method^27,41^ and ab initio many-body perturbation theory, we show that both the inclusion of phonons and the presence of bound water have large effects on band edge energies, fundamental gaps, optical gaps and exciton binding energies. Specifically, our calculations predict phonon-induced localization of excitons, the extent of which is hydration-dependent. To validate our findings, we synthesize SU-101 as reported in the literature^37^, develop an activation protocol, and measure its ultraviolet-visible (UV-Vis) absorption and photoluminescence (PL) emission spectra. The good agreement with experimental trends in optical absorption allows us to rationalize changes in the UV-Vis spectra upon bound solvent removal. Altogether, our work highlights how exciton-phonon interactions can be significant for MOFs, and lead to important changes even with relatively small structural modification.

## Results

### Structural effects with and without water

SU-101 is composed of ellagate organic linkers and rod-shaped metal nodes, with Bi being further coordinated by bound solvent (water). For more details on this MOF, we refer the reader to the Supplementary Information Section 1. To examine the structure of SU-101 with and without bound water, we synthesize SU-101 and obtain two derived samples (Supplementary Information Sections 2.1 and 15 for more details): 1) bound water (as-made, with free water removed), and 2) activated SU-101 (fully dehydrated). Upon removal of coordinated water, Le Bail refinement of the PXRD data in Fig. S1 reveals a contraction along the *a* and *b* axes (~3%), and an expansion along the *c* axis (~3%). The shift of the low-angle (110) reflection to higher 2*θ* is consistent with a reduction in *d*-spacing within the *ab* plane, while the expansion along *c* is determined from the refined lattice parameters. The *c* axis is the direction in which SU-101’s metal nodes arrange in “channels” in a rod-like shape. Therefore, activating SU-101 leads not only to an exposure of open metal sites^39^, but also to a contraction in the direction of the rod-shaped metal node.

**Fig. 1:**
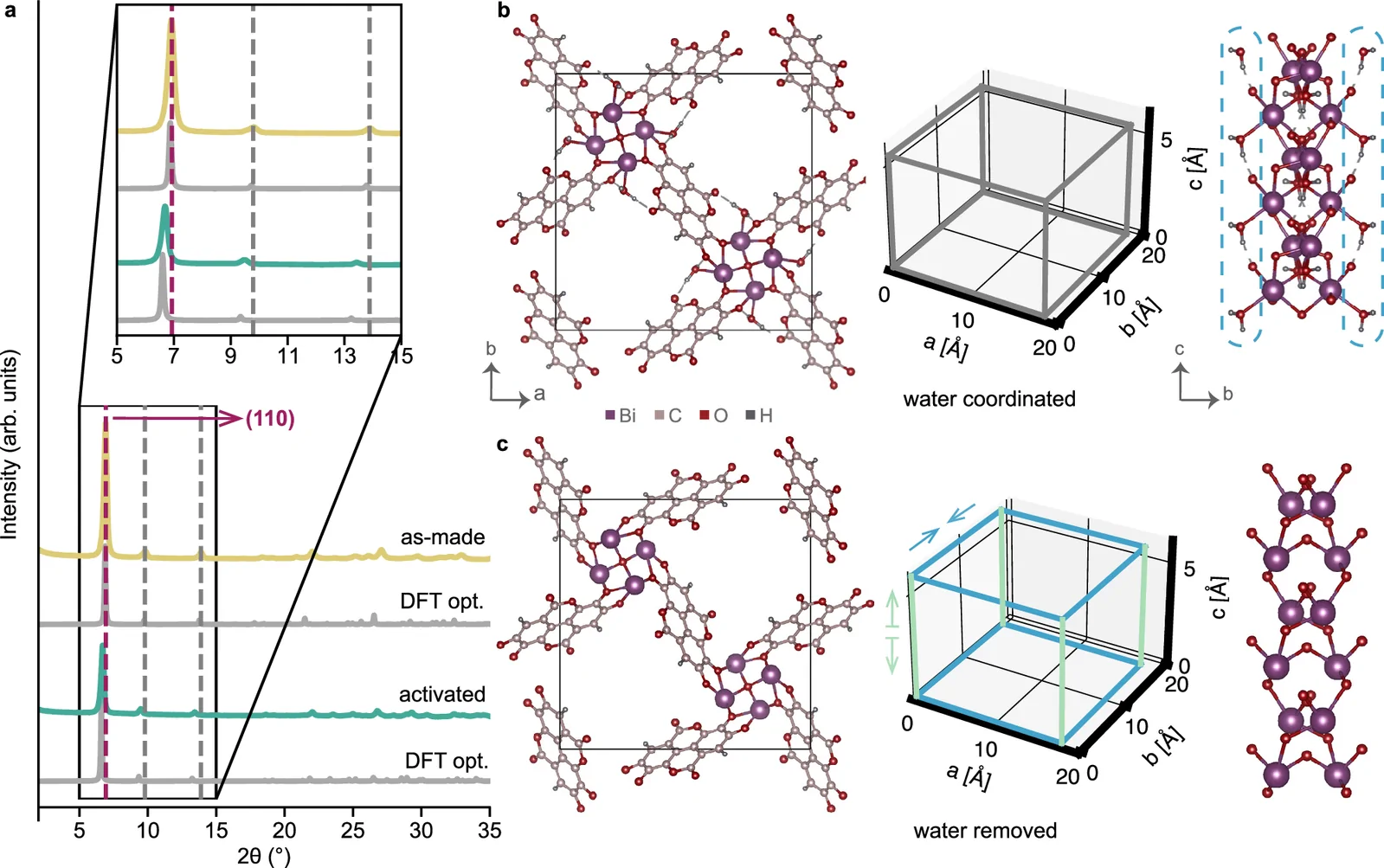
Structural changes upon bound water removal. **a** X-ray powder diffraction profile for the as-made (water-coordinated) and activated (water removed) material and comparison with the simulated profile from DFT structure optimization. View in *ab* plane, cell parameters, and view of rod-shaped metal node array along the *c* axis for **b**. Water-bound SU-101, and **c** activated SU-101, obtained from a previously reported structural model (CCDC 2003313^37^). Water-bound SU-101 is obtained by DFT-optimizing the structure after free solvent removal, whereas the activated SU-101 is obtained by DFT-optimizing the structure after removing all water molecules. Reduction of *a* and *b* lattice parameters upon removal of coordinated water is shown in blue, and increase of *c* in green, (see Table 1 for values); H-bonds highlighted.

To understand the structural changes in SU-101 upon water removal, we perform structural relaxation with density functional theory (DFT) using the Perdew-Burke-Ernzerhof (PBE) functional^42^ (see “Density functional theory calculations”). The DFT calculations are performed on the primitive cells of both bound water (132 atoms) and activated (108 atoms) SU-101 (space group *P*4_2_/*n*). Our calculations show a contraction of *a* and *b* of ~4%, and an expansion of *c* axis of ~7%, in good agreement with the measured anisotropic changes of the lattice parameters (Table 1). The changes in lattice parameters are due to the loss of H-bonds between Bi-coordinated water and neighboring O atoms, which lead to shorter Bi-Bi distances in water-bound SU-101, and potentially improved *π*-*π* stacking (Fig. 1). Partial hydration would likely result in structural changes between those observed for the bound water and fully activated states.

**Table 1:** Experimental and calculated lattice parameters for SU-101 before (bound water) and after (activated) removal of coordinated water

Case	*a* = *b* (Å)	*c* (Å)	*α* = *β* = *γ* (^∘^)	*V* (Å^3^)
Bound water				
Theory	18.91	5.55	90	1983.52
Experiments	18.61 (7)	5.54 (1)	-	-
Activated				
Theory	18.18	5.95	90	1967.16
Experiments	18.03 (7)	5.71 (4)	-	-

An underestimate of the true fundamental gap (see “Excitonic effects computed without phonons”), the DFT-PBE gap of SU-101 is reduced by ~0.3 eV upon bound water removal (1.91 eV for hydrated, 1.62 eV for fully activated SU-101). The band structures in Fig. S2 reveal that the bound water does not contribute to the valence and conduction band states. Instead, the band gap reduction originates from structural changes associated with water removal, which promotes breaking of H-bonds and elongation of Bi-Bi distances. The presence of Bi with its polarizable core and the degree of Bi-Bi spacing also affects the optoelectronic properties of SU-101, e.g., via contributions to dielectric screening.

### Excitonic effects computed without phonons

In light-harvesting systems, particularly organic systems, light absorption and related optical properties are often dominated by excitonic effects, or effects associated with photoexcited electron-hole pairs bound by the screened Coulomb interaction. In MOF-5, a commonly studied MOF with Zn clusters and 1,4-benzo dicarboxylic acid linkers, the exciton binding energy was reported to be substantial, on the order of a few eV^29^. Although studies on exciton binding energies in MOFs are rare, MOFs can host strongly bound excitons due to their inherent porosity, low polarizability, and consequent weak dielectric screening^29,30^. Given the relevance of excitons in MOFs, we investigate the excited-state and excitonic properties of SU-101, employing the ab initio GW-Bethe-Salpeter equation (BSE), as implemented in the BerkeleyGW package^43–45^.

**Fig. 2:**
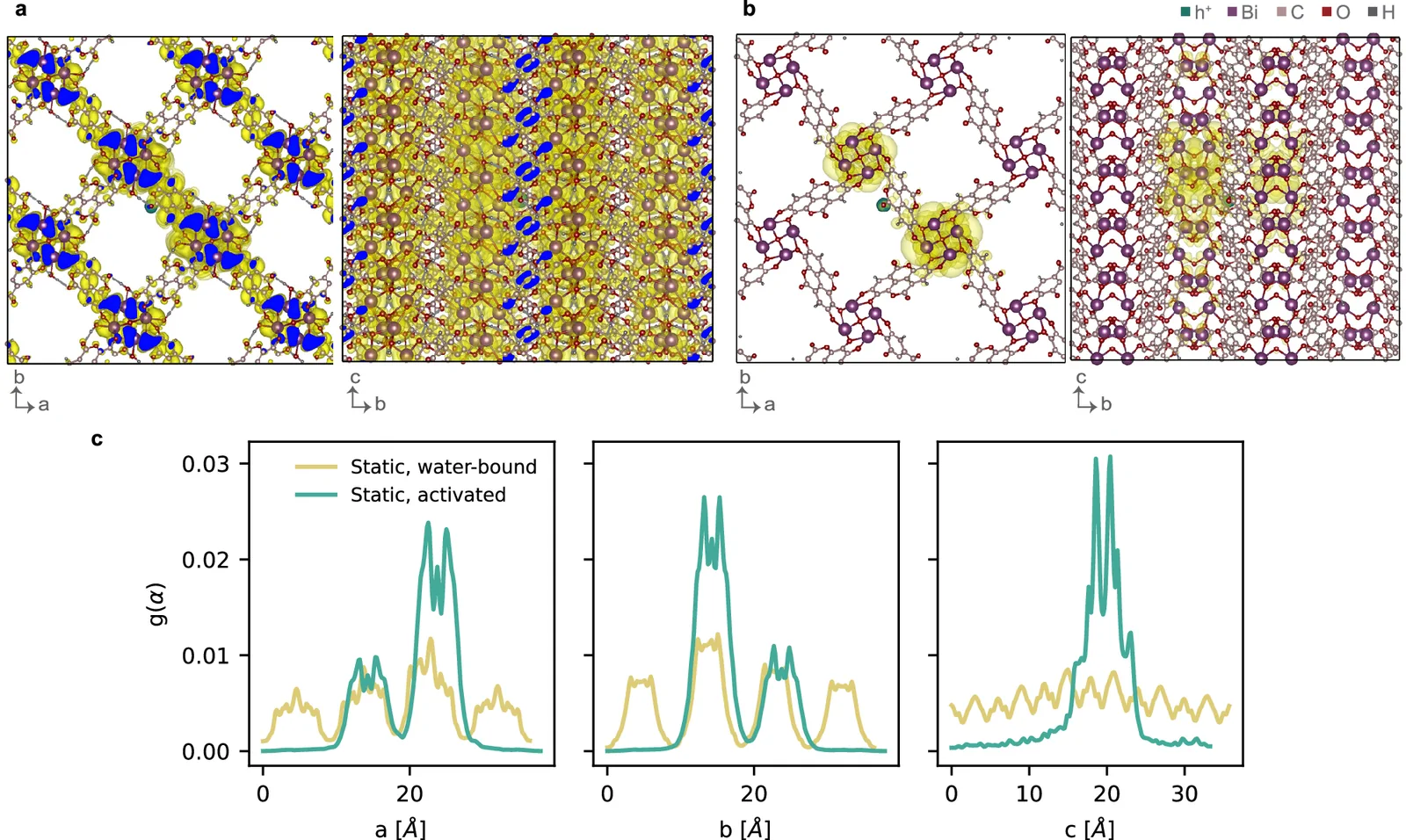
Changes in first exciton localization upon bound water removal. First exciton (singlet, fixed hole at center of unit cell) for **a** water-bound and **b** activated SU-101. Isosurfaces are shown enclosing 80% of the exciton probability density, with the corresponding isosurface values (water-bound: 10, activated: 558.5). Blue regions correspond to isosurface cross-sections. **c** For both water-bound and activated SU-101, we plot the planar averaged electron-hole correlation function *g*(*α*) (*α* is the direction of interest)^66^, which provides a profile of the probability of finding an electron given a hole fixed at the origin (see “Post-processing” and SI Section 14). The range and origin in Å in the *x* axis of **c** for all lattice parameters correspond to those of the 2 × 2 × 6 supercells represented in (**a**, **b**).

With descriptors based on prior ground-state DFT calculations (Supplementary Information Section 3)^46^, we find that the water loading in SU-101 could influence charge separation. To evaluate this effect further, we perform ab initio GW-BSE calculations on the system with (water-bound/hydrated, ${{{\rm{Bi}}}}_{2}{{\rm{O}}}{({{{\rm{H}}}}_{2}{{\rm{O}}})}_{2}({{{\rm{C}}}}_{14}{{{\rm{H}}}}_{2}{{{\rm{O}}}}_{8})$) and without (activated, Bi_2_O(C_14_H_2_O_8_)) coordinated water molecules at Bi sites. We first evaluate their optoelectronic properties without the effect of phonons. Water-bound SU-101 is computed to have a quasiparticle gap of 3.02 eV and a lowest optical (bright) transition at 2.93 eV. This leads to a relatively modest exciton binding energy of 90 meV, which suggests that excitons are somewhat more delocalized in this MOF (Fig. 2a) than in MOF-5, which has a reported exciton binding energy of ~3.5 eV. The reduced exciton binding energy in water-bound SU-101 compared to MOF-5 originates from the details of the different linkers and pore sizes, as well as the presence of more polarizable Bi atoms in SU-101. Bi contributes states to the conduction band edge and to its relatively higher computed clamped-ion dielectric constant (*ϵ*_*∞*_ = 3.195), compared to MOF-5 (*ϵ*_*∞*_ = 1.47^47^).

On the other hand, we calculate that activated (water-free) SU-101 has a fundamental *G*_0_*W*_0_ gap of 2.8 eV, with a first optical (bright) transition at 2.36 eV. The excitons in activated SU-101 are more strongly bound, with an exciton binding energy of 440 meV. This increase in exciton binding energy over that in the hydrated MOF suggests an increase in exciton localization (Fig. 2b) and is consistent with reduced screening effects without water (and somewhat smaller computed clamped-ion dielectric constant, *ϵ*_*∞*_ = 3.034). Tables 2 and 3 summarize these results.

**Table 2:** *G*_0_*W*_0_-BSE fundamental and optical gaps (lowest exciton) of water-bound and activated SU-101

Case	Fundamental gap [eV]^1^			Optical gap [eV]^1^		
	No ph.	0 K	300 K	No ph.	0 K	300 K
Bound water	3.02	2.85 ± 0.03	2.75 ± 0.03	2.93	2.60 ± 0.02	2.46 ± 0.06
Activated	2.80	2.51 ± 0.03	2.38 ± 0.02	2.36	2.14 ± 0.03	1.98 ± 0.04

The values are reported without (no ph.) and with the effect of phonons, computed at 0 K and 300 K.

^1^The errors are due to sampling statistics only.

**Table 3:** *G*_0_*W*_0_-BSE exciton binding energies (EBE) of water-bound and activated SU-101

Case	Exciton binding energy [meV]^1^		
	No ph.	0 K	300 K
Bound water	90	230 ± 23	292 ± 38
Activated	440	392 ± 15	403 ± 23

The values are reported without (no ph.) and with the effect of phonons, at 0 K and 300 K. Here, the exciton binding energy is simply the difference between the optical and fundamental gaps (see Table 2).

^1^The errors are due to sampling statistics only.

The increase in localization of the lowest exciton upon water removal can be seen in Fig. 2, where the isosurfaces represent electron probability density when the hole is fixed at the geometric center of the organic linker, positioned in the central region of a 2 × 2 × 6 unit cell. The choice of the hole position is justified as a high-probability one by the character of the valence bands, which are predominantly composed of C and O *p*-orbitals (see details in “Post-processing”). Fig. 2a shows that the lowest exciton of water-bound SU-101 (2.93 eV) is delocalized in all directions, both in the *ab* plane and along *c*. On the other hand, the lowest-lying exciton in activated SU-101, with an energy of 2.36 eV, is overall more localized, with stronger localization in the *ab* plane (Fig. 2b) The greater localization along *c* relative to the hydrated case can be explained by the axis elongation upon activation reported in Fig. 1c, which reduces coupling between nodes along the rod axis. The separation of the electron and hole across different MOF building blocks suggests significant charge-transfer character.

The polarization dependence of the computed absorption spectra reflects the spatial anisotropy of the exciton along the direction of the metal node and the perpendicular directions, as shown in Fig. S3. Water-bound SU-101 also exhibits polarization dependence in its computed absorption spectrum (Fig. S4), though to a lesser extent than the activated MOF. In both cases, there is increased absorption for light polarized along the metal nodes at lower energies compared to polarization along the *ab* directions. Therefore, our BSE results confirm that the rod-shaped metal node arrangement plays an important role in determining the excitonic properties of SU-101. Lastly, in the absence of phonons, we predict that the water removal will cause a significant shift in the onset of absorption (Figs. S5 and S6).

In summary, our GW-BSE results in the absence of phonons indicate significant differences in the degree of exciton delocalization upon water removal, more intense absorption for light polarized along the *c* axis, and a shift in the onset energy of optical absorption.

### Phonon effects on excitonic properties

The interaction between electrons and phonons plays a crucial role in determining key properties relevant to light-related applications, such as the electronic band gap, optical behavior, charge carrier mobility, rate of exciton dissociation, and the nature of excitons^27,36,48–50^. In halide perovskites, excitonic effects and EPC are known to impede carrier transport and facilitate detrimental electron-hole recombination^51^. In organic semiconductors, strong EPC has been associated with large nonradiative energy loss^28,52^, potentially providing pathways for charge recombination that impair photocatalytic efficiency. Moreover, EPC has been shown to induce exciton localization^27^, which can hinder the extraction of free charge carriers to participate in photo-redox reactions^53^. In MOFs, we expect EPC to be strong due to low-frequency optical phonons associated with the organic linkers.

We compute the effect of phonons for both activated and water-bound SU-101, using a finite displacement method^27,41^. Nonadiabatic effects are neglected here, an acceptable approximation for organic linkers and in the context of steady-state measurements^27,54^. As shown in Fig. S7, the harmonic approximation is justified by the good agreement between the harmonic and anharmonic potential energy surfaces for the optical modes with frequencies of 18.8 cm^−1^ and 3071.2 cm^−1^ (without spin-orbit coupling, SOC), and 18.8 cm^−1^ (with SOC). Such agreement can be attributed to the aromatic and polycyclic nature of the linkers in SU-101, when compared to linkers with softer degrees of freedom, such as succinic acid. Thus, we generate displaced configurations within the harmonic approximation and perform Monte Carlo sampling^55^ to compute the vibrational averages of the DFT-PBE gaps at 0 K and 300 K of both activated and water-bound SU-101 (Table S1). We evaluate phonon effects at these temperatures to disentangle the relative contributions of quantum nuclear motion and finite-temperature effects, particularly at room temperature, which most closely reflects typical experimental conditions.

To evaluate the effect of phonons on excitonic and optical properties, we perform GW-BSE calculations on 10 representative configurations sampled with DFT-PBE at 0 K and 300 K and for both water-bound and activated SU-101 (red marks in Figs. S8 and S9). We choose configurations that best capture the average renormalization of the PBE gap, as highlighted by the red marks in Fig. S8. This sampling approach has been utilized for molecular crystals in previous studies^27^. For activated SU-101, the zero-point renormalization of the GW quasiparticle gap is 310 ± 28 meV due to the effect of phonons. At 300 K, the gap is further reduced by an additional 120 meV, resulting in a total renormalization of 430 ± 25 meV. For the water-bound SU-101, we compute a zero-point renormalization of 176 ± 34 meV, and this effect is enhanced by 93 meV at 300 K, resulting in a total renormalization of 269 ± 34 meV. Altogether, the vibrational renormalization of SU-101’s DFT-PBE gap under different levels of hydration is dominated by zero-point effects. These values are summarized in Table 2, Tables S1 and S2.

**Fig. 3:**
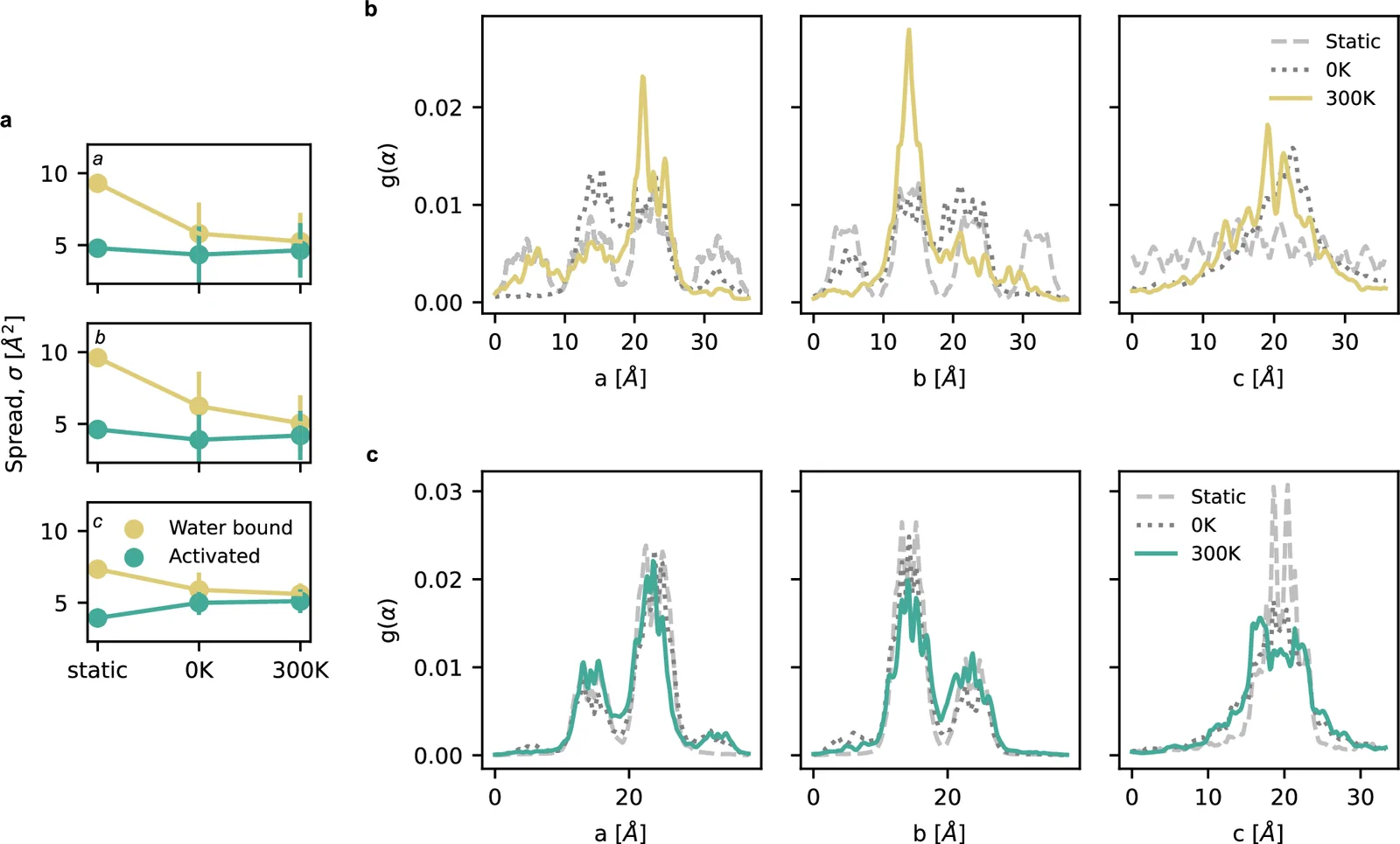
Impact of phonons on the first exciton of both systems. **a** Change in the exciton delocalization (directional spread, see details in the “Post-processing”) per axis for the water-bound and activated SU-101. This spread is given by the standard deviation, over the real space grid and in each direction of interest, of the planar averaged electron-hole correlation function. For **b** water-bound and **c** activated SU-101, we plot the planar averaged electron-hole correlation function *g*(*α*) (*α* is the direction of interest)^66^, which provides a profile of the probability of finding an electron given a hole fixed at the origin (see “Post-processing”, and SI Section 14). These quantities are plotted for the static case, and for the averaged 10 configurations at 0 K and 300 K. The range and origin in Å in the *x* axis of **b**, **c** for all lattice parameters correspond to those of the 2 × 2 × 6 supercells represented in Fig. 4a, b.

Our BSE results show that phonons play a pivotal role in whether excited states are localized or delocalized, consistent with prior findings in organic crystals^27^. For example, if we focus on water-bound SU-101, including phonons strongly increases exciton localization in all directions, as displayed in Fig. 3a, b. For the activated SU-101, the change in localization is much more modest, as shown in Fig. 3a, c. After water removal, we find that there is a small increase in exciton delocalization along the metal node in *c* upon inclusion of phonons, whereas in *a*, *b* directions the exciton localization is practically unchanged.

To quantitatively evaluate how phonons impact exciton localization on both water-bound and activated SU-101, we evaluate the direction-dependent spread of the exciton using electron-hole correlation functions, as described in the “Post-processing”. Larger spreads indicate greater exciton delocalization in real space. Figure 3a shows the computed spread in *a*, *b* and *c* directions for SU-101 for both water-bound and activated cases, without and with phonons at 0 K and 300 K.

Notably, for the activated SU-101, phonons slightly increase the spread in the *c* direction, along which the metal nodes are arranged. While phonon-induced exciton delocalization can sometimes manifest as an artifact of neglecting anharmonic phonons, as for pentacene^27^, the effect of anharmonicity is significantly less pronounced in SU-101 (see analysis of low- and high-frequency modes in Fig. S7). When considering all three directions simultaneously, the overall spread remains largely unchanged with phonons, consistent with the similar exciton binding energies (see Table 3).

Perhaps the most significant effect is the phonon-driven localization of excitons for the water-bound SU-101. This effect is similar to what was computed for molecular crystals^27^, and is different from what we calculated for the activated case. At 0 K, phonons increase exciton localization in all directions. At 300 K, the localization is greater and closely matches that of the activated MOF. The localization arises from dynamic disorder—phonon-driven symmetry breaking associated with atomic displacements away from their equilibrium positions—rather than from changes in the dielectric properties, which, in polar materials, can lead to exciton delocalization^36^.

To understand the origin of the stronger phonon-induced exciton localization in water-bound SU-101, we analyze the 20 largest atomic displacements in the Monte Carlo-sampled configurations at 0 K and 300 K (Fig. S10). In this analysis, larger displacements indicate a greater contribution of these atoms to the renormalization of the exciton energies. In the activated SU-101, C atoms involved in C=C bonds rank among the most displaced. In contrast, significant displacements of lactone O and water atoms are observed only in the water-bound structure. These observations suggest that hydration modifies the atomic degrees of freedom that couple to the exciton, leading to distinct exciton-phonon coupling mechanisms in the two cases.

**Fig. 4:**
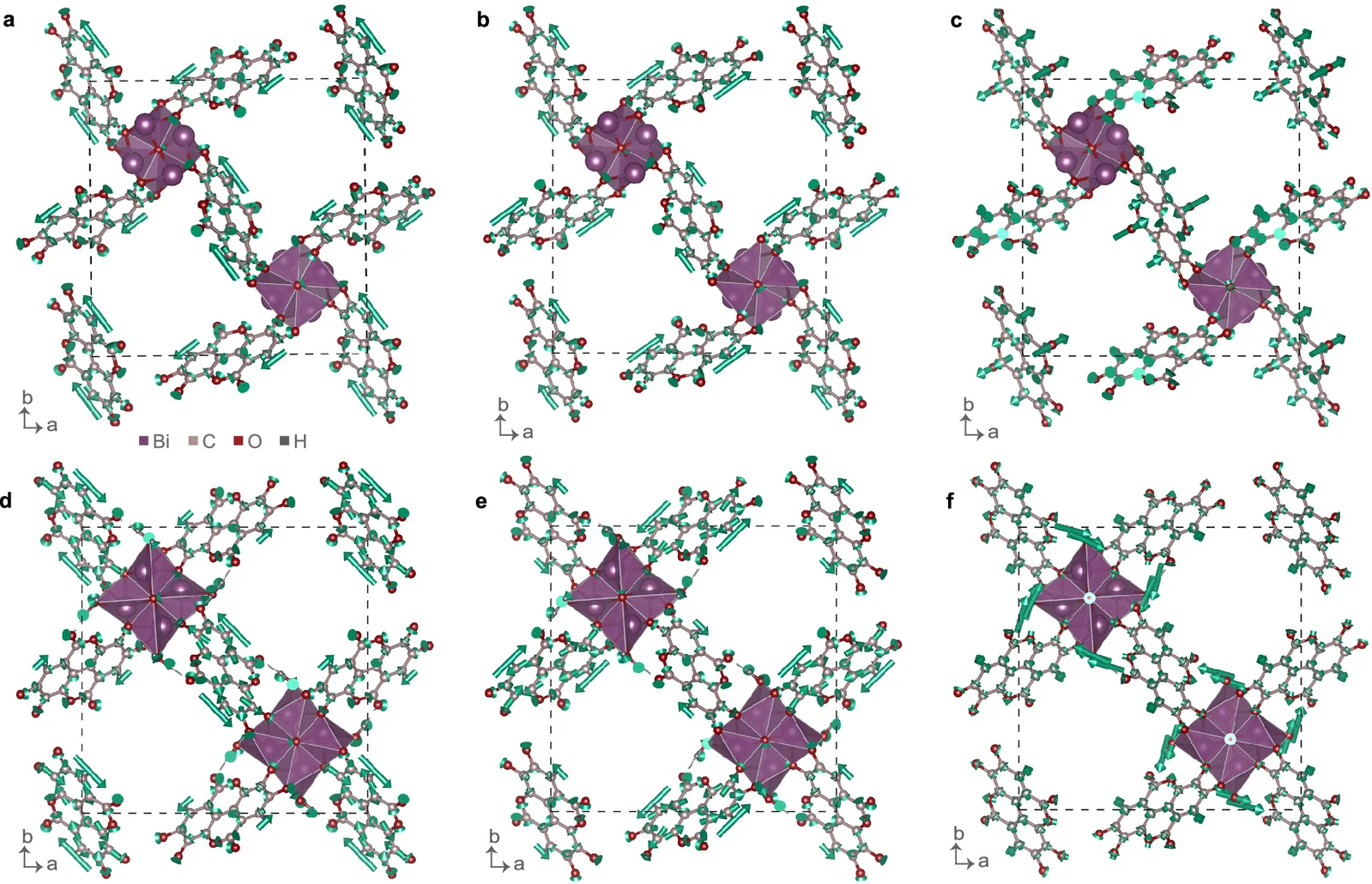
Relevant phonon modes to the exciton-phonon coupling in both systems. For the activated SU-101, modes with the **a** highest (1474.88 cm^−1^, Supplementary Movie 1) and **b** second highest contribution to the Kohn–Sham (KS) gap renormalization at 0 and 300 K (Supplementary Movie 2), and **c** a mode of particular relevance at 300 K (92.59 cm^−1^) (Supplementary Movie 3). For the water-bound SU-101, modes with the **d** highest (1484.43 cm^−1^, Supplementary Movie 4) and **e** second highest contribution to the Kohn–Sham (KS) gap renormalization at 0 and 300 K (Supplementary Movie 5), and **f** a mode of particular relevance at 300 K (202.06 cm^−1^, Supplementary Movie 6). Supplementary Movies 1–6 show the animated modes and are available in the Supplementary Information.

To clarify the mechanism behind the hydration-dependent EPC, we analyze the individual phonon mode contributions to the band gap renormalization in both systems (Supplementary Information Section 6.4). The phonon modes that contribute the most to the gap renormalization of activated SU-101 are the zero-point aromatic ring vibrations, such as the asymmetrical C=C stretching modes (~1450 cm^−1^, see Figs. 4a, b and  S11) at 0 K; and a few lower frequency modes excited at 300 K (~90 cm^−1^, see Fig. 4c), associated with a collective tilting of the ellagate linker and Bi-O stretching. For water-bound SU-101 (Figs. 4d, e and  S12), the important phonon modes at 0 K (~1480 cm^−1^) are very similar to those of activated SU-101. However, at 300 K (Fig. 4f), low-frequency modes associated with the rotation of the Bi-OH2 bond (~200 cm^−1^) also contribute for water-bound SU-101. These modes are not present in the activated MOF, for which a collective tilting of the linker is the major contributor at 300 K. Our findings demonstrate that different phonons impact excitonic properties at 300 K upon bound water removal, and explain the origin of the hydration-dependent phonon-induced exciton localization.

Strong phonon-driven exciton localization in water-bound SU-101 may influence photocatalysis in non-trivial ways. Photocatalysis reflects a balance of multiple processes across different timescales that may compete. Phonon effects, including those beyond the adiabatic limit, can be either detrimental or desired (e.g., ultrafast exciton dissociation^56^) for photocatalysis, and are oftentimes competing. Moreover, although exciton localization is often linked to non-radiative recombination, this pathway is not necessarily rate-limiting in carrier generation. Therefore, the overall impact of exciton-phonon coupling on photocatalysis remains system-dependent, and our results provide a first step toward its microscopic understanding.

In summary, the large differences in the spatial extent of the exciton in water-bound and activated SU-101 without phonons become minimal when considering phonons. This behavior is due to the differences in exciton-phonon coupling with and without bound water, especially at 300 K, where different low-frequency phonon modes become relevant to the overall phonon effects when bound water is removed. While this study is limited to a specific MOF, we expect that phonons and excitons play an important role for MOF photocatalysts in general as they do here when the low-energy excitations are dominated by flexible organic linkers.

### The interplay of bound solvent and exciton-phonon effects

Investigating the interplay between excitons and phonons is crucial to understand the potential differences in the optoelectronic properties of water-bound and activated SU-101. Our calculations suggest that phonons have different effects on the spatial localization of the excitons of water-bound and activated SU-101. With phonons, we predict that the charge-transfer character of excitons upon water removal should not vary significantly at 300 K given the similar behaviors in Fig. 3b, and that the exciton binding energies should show less pronounced differences.

**Fig. 5:**
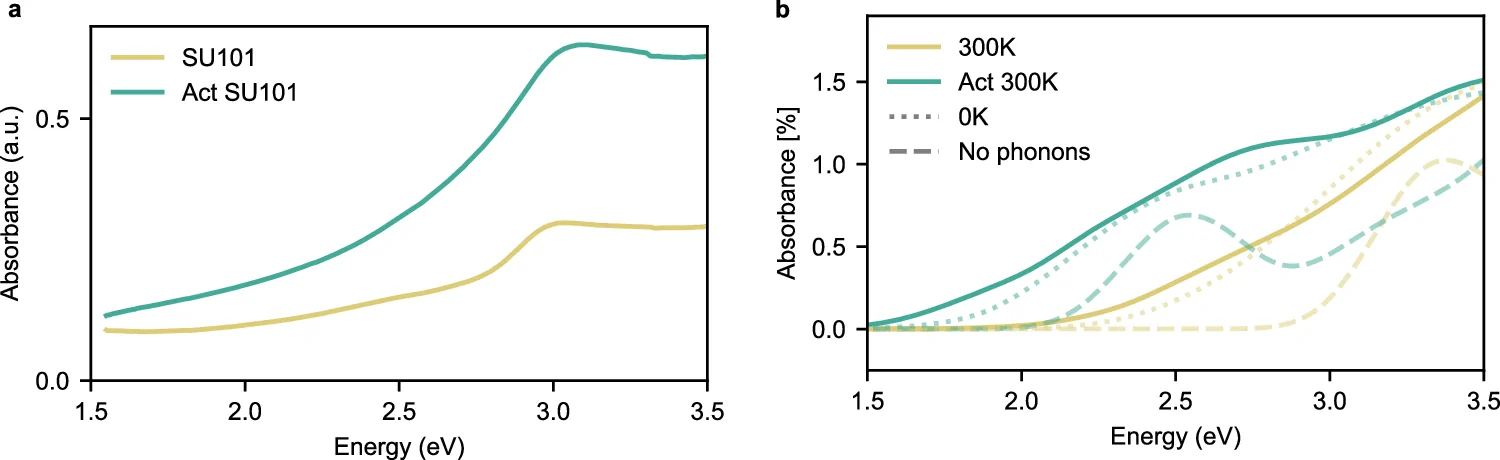
Experimental comparison of the absorption spectra of both systems. **a** UV-Vis absorption spectra of the as-made (yellow) and activated (blue) materials of SU-101 measured under the same conditions. **b** *G*_0_*W*_0_-BSE absorbance spectra of water-bound SU-101 and activated SU-101 (see “Post-processing”), where yellow and blue curves show results for the as-made and activated system, respectively, without phonons (long dashed lines), at 0K with zero-point motion (dotted lines), and at 300K (solid lines). Phonon effects are averaged over 10 displacements (see Supplementary Information Section 13).

Water removal from SU-101 reduces the computed optical gap (Table 2) and red-shifts the calculated absorption onset. Without considering phonons, this results in sharper peaks separated by ~1 eV, with little overlap in the range of 1.5 eV–3 eV (see dashed lines in Fig. 5b). Moreover, we note that the first absorption peak of water-bound SU-101 (in yellow) has a higher absorption intensity than that of activated SU-101 (in blue). However, the inclusion of phonons causes significant changes in the calculated BSE spectra. Firstly, phonons at 300 K slightly reduce the differences in the absorption onset of water-bound and activated SU-101 (see solid lines in Fig. 5b). Additionally, upon inclusion of phonons and specifically at 300 K, the intensity of the lowest energy peak is significantly reduced for water-bound SU-101. Therefore, the immediate consequence of considering phonons is that, in the 2 eV to 3 eV energy range, absorption in water-bound SU-101 at 300 K is half as intense as that of the activated SU-101.

To investigate whether these features can be seen experimentally, we measure both UV-Vis absorption and PL emission spectra of water-bound and activated SU-101 (Figs. 5a and  S13b). Both the as-made and activated SU-101 show a similar UV-Vis spectral character. We notice a slight decrease in the Tauc plot-derived optical gap upon water removal (Fig. S14, Supplementary Information Section 7 for more details), and a consistent redshift in the emission spectra (Fig. S13). These differences are not large, which is likely attributed to the difficulty in avoiding water exposure and potential disorder in the Bi coordination environment if not all water is removed. Still, the differences in measured absorption spectra follow the same trend as observed from our calculated results. Disorder can also explain the lower intensity and broader emission peak for the activated SU-101 (Fig. S13).

The most intriguing aspect is that our experiments show an almost two-fold increase in absorption at ~2.5 eV when bound water is removed. This is consistent with what we predict from our BSE results (Fig. 5b), where the computed spectra show a near two-fold increase in the range of ~2 eV to 3 eV upon water removal. Our BSE results suggest that the increase in absorption in this range stems from the redshift of the optical gap upon water removal (see Table 2), together with phonon effects that contribute to small but non-negligible oscillator strengths in a lower energy range for the water-bound SU-101. Without considering phonons, one would have expected the water-bound SU-101 to display a more intense first absorption peak than the activated SU-101. Therefore, inclusion of phonons in our calculations is crucial for understanding the observed experimental trend, where activated SU-101 shows more intense absorption.

Lastly, we have performed time-resolved PL measurements. The average PL lifetime increases from 2.496 ns in the water-bound material to 2.849 ns after activation, as shown in Fig. S15. This change suggests a minor modification of the overall excited-state decay rate upon dehydration. As such, these results are consistent with changes in the local coordination environment affecting exciton-phonon coupling and potential relaxation pathways.

## Discussion

One of the key advantages of MOFs for light-related applications is their tunable and modular nature, allowing one to choose visible-light-absorbing linkers while still observing a significant influence of metal centers on the optoelectronic and (photo)catalytic properties. Additionally, because of their complex modular nature, the effect of phonons on excitons can be quite large in MOFs when compared to classic inorganic semiconductors.

Here, we demonstrated how exciton-phonon coupling can significantly impact the optoelectronic properties of SU-101 at varying water loadings. While we predict that both water-bound and activated SU-101 have similar strongly bound excitons at 300 K, the way they couple to phonons is hydration-dependent, with stronger exciton localization due to phonons for water-bound SU-101. Although in-situ studies could capture real-time hydration effects, our first-principles analysis already reveals the main trends in exciton–phonon coupling and its hydration-dependency in SU-101. Furthermore, our results indicate that accounting for phonons and excitons is important for interpreting the changes in the measured UV-Vis absorption spectra upon bound water removal. Lastly, exciton-phonon coupling and its sensitivity to changes in the local chemical environment can influence multiple aspects of photocatalytic processes, including light absorption, and subsequent exciton dynamics and dissociation into free charge carriers. Therefore, a comprehensive view on exciton-phonon coupling in materials with strong excitonic effects is essential for interpreting experimental observations and guiding photocatalyst design.

In SU-101, the impact of bound solvent arises indirectly through structural modifications, where solvent-derived hydrogen bonds modulate metal-metal distances and consequently the electronic structure of this MOF. Other MOFs with bound solvents may exhibit similar behavior, provided that the solvent induces comparable changes in the atomic geometry. More generally, we expect strong excitonic effects and exciton-phonon coupling to be common features of MOFs, particularly those with soft degrees of freedom associated with organic linkers and large pores.

## Methods

### DFT calculations

Unless stated otherwise, we use QUANTUM ESPRESSO v.7.2^57^ for all DFT calculations of the structures and their Kohn-Sham electronic properties. We employ the semi-local PBE exchange-correlation functional^42^ with semi-empirical Grimme’s DFT-D3 dispersion correction^58^. A plane-wave energy cut-off of 96 Rydberg is utilized based on convergence tests, and Marzari-Vanderbilt-DeVita-Payne cold smearing^59^ is used for our calculations. We use a Monkhorst-Pack k-point grid of 1 × 1 × 3, where *c* is the direction of the smallest cell dimension. Pseudopotentials are chosen from the norm-conserving pseudopotential table (Pseudo Dojo^60,61^ v0.5) for the PBE functional and with standard accuracy. For SOC calculations, the full-relativistic version of the pseudopotentials (v0.4) is used instead. Variable-cell structure relaxation is performed until forces and total energies are converged within 1 × 10^−5^ a.u. and 1 × 10^−6^ a.u., respectively.

### Electron-phonon coupling

We compute EPC using DFT by means of the finite differences approach^41^, in a similar fashion to what was reported for molecular crystals^27^. The atoms in the system are explicitly displaced by 0.06 Bohr, and the electronic response to the displacements is evaluated by computing the change in eigenvalue with each displacement. We rely on the Born-Oppenheimer approximation and assume the nuclear motion to be harmonic. To account for the effect of a temperature *T* beyond zero-point effects, we compute the ensemble average according to Equation (1)^27^ with the partition function $Z=\int\,{{\rm{d}}}X\exp \left(-\beta {{\mathcal{H}}}\right)$ where *H* is the Hamiltonian of the system that, in this study, assumes a harmonic nuclear motion.

1 $${\langle {{\mathcal{O}}}(T)\rangle }_{{{\mathcal{H}}}}=\frac{1}{Z}\int\,{{\rm{d}}}X\,{{\mathcal{O}}}(X)\exp \left(-\beta {{\mathcal{H}}}\right)$$

We test for possible deviations from the harmonic approximation by performing explicit displacements along a low-frequency and a high-frequency phonon mode, computing the total energy, and comparing the actual potential energy surface behavior with that of a perfectly harmonic system with the same frequency through fitting. According to Fig. S7, even at larger displacements, the harmonic approximation seems to hold.

Through a Monte Carlo sampling of vibrational averages, 100 configurations are chosen according to the harmonic vibrational ensemble at each temperature *T* (0 K, 300 K). Tests are performed to ensure that the 100 configurations suffice for convergence (Fig. S8). The observable of interest, e.g., Kohn–Sham gap, is calculated at each displaced configuration, and we compute the average over all configurations. To evaluate excited-state and related properties of interest for photocatalysis, we select 10 configurations to proceed with that better capture the average effect of the overall sampling (red marks in Fig. S8).

We evaluate the contributions of individual phonon modes to the renormalization of the Kohn-Sham bandgap at 0 K, 100 K, and 300 K, following the harmonic quadratic formulation^26^. A clear advantage of this formulation is that it provides a mode-resolved picture of the band gap renormalization. This approach expands the electronic observable to second order in phonon displacements, allowing its temperature dependence to be expressed as the sum of the static (no-phonon) value and the contributions from each phonon mode. In short, the method consists of a quadratic expansion of the observable ${{\mathcal{A}}}({{\bf{u}}})$ with respect to the phonon amplitudes *u*_**q***ν*_, namely

2 $${{\mathcal{A}}}(T)\approx {{\mathcal{A}}}({{\bf{0}}})+\mathop{\sum}\limits_{{{\bf{q}}},\nu }\frac{1}{2{\omega }_{{{\bf{q}}}\nu }}\frac{{\partial }^{2}{{\mathcal{A}}}}{\partial {u}_{{{\bf{q}}}\nu }^{2}}\left[\frac{1}{2}+{n}_{{{\rm{B}}}}({\omega }_{{{\bf{q}}}\nu },T)\right]$$

The key quantity is the second derivative of the observable with respect to the displacement of each phonon mode. In practice, we calculate this derivative using a finite-difference approach, which requires evaluating the band gap at positive and negative displacements for each mode, as in Equation (3). This means performing two DFT calculations per phonon mode.

3 $$\frac{{\partial }^{2}{E}_{g}}{\partial {u}_{\nu }^{2}}\approx \frac{{E}_{g}(\delta {u}_{\nu })+{E}_{g}(-\delta {u}_{\nu })-2{E}_{{{\rm{static}}}}}{\delta {u}_{\nu }^{2}}$$

In Equation (2), ${{\mathcal{A}}}(T)$ is described in terms of ${{\mathcal{A}}}({{\bf{u}}})$ by taking the vibrational average, and further truncating the terms which are beyond quadratic. In this equation, the diagonal quadratic terms describe the electron-phonon interaction.

### Ab initio GW-BSE calculations

To compute the quasiparticle properties of SU-101, we employ the single-shot G_0_W_0_ approximation as implemented in the BerkeleyGW code^43–45^. After testing for convergence (Supplementary Information Section 11), we opt for a 2 × 2 × 6 k-point grid, 2500 bands, and a 15 Ry plane-wave cut-off to calculate the dielectric screening. A q-shift of 0.001 is used on the *x* axis. For the displaced configurations, we reduce the computational cost by assuming that the change in the dielectric properties is minimal from the static (no-phonon) case to the displaced cases (Supplementary Information Section 11.4). In organic systems, the majority of the phonons are non-polar^62^ and do not contribute to the static dielectric constant. Assuming a similar behavior for our material, we use the dielectric function from the static (no-phonon) structure (in Supplementary Information Section 11.4, we test this approximation for a couple of displaced structures to validate our approach). For exciton calculations, the electron-hole kernel is computed on a 2 × 2 × 6 grid using 20 valence and 20 conduction bands (or immediately lower or higher values when those are not allowed by symmetry). Then, we interpolate on a finer 4 × 4 × 12 grid using 10 valence and 10 conduction bands to perform the absorption calculation. On the finer grids, the k-points are shifted by [0.05, 0.13, 0.07] to avoid *Γ*-centering. We compute SOC at the PBE level, and treat it perturbatively at the GW level^63^. This approximation is acceptable as the PBE band structure of SU-101 presents only systematic shifts for the Bi states when including SOC, and does not present a qualitative change in behavior (see Fig. S23). The effect of SOC on the eigenvalues is captured from the PBE calculation by comparing matrix element overlaps, which allows us to identify corresponding states. The BSE is then computed using the quasiparticle energies corrected for SOC.

### Post-processing

For band alignment, we follow the procedure established in literature^13,64^ to compute the electronic chemical potential of reference as the region inside the pores where the variation of the potential is the lowest. We modify the available MacroDensity python package for QUANTUM ESPRESSO and utilize the total potential (${V}_{{{\rm{bare}}}}+{V}_{{{\rm{H}}}}+{V}_{{{\rm{xc}}}}$) for analysis. The as-obtained vacuum reference is also utilized for alignment in the quasiparticle picture, under the assumption that it is a good approximation for the band alignment at the GW level^65^.

Effective masses for top valence/bottom conduction bands were computed within the parabolic approximation by taking the second derivative with respect to k. For the quasiparticle calculation, an interpolation to the fine 4 × 4 × 12 k-grid is performed to obtain a fine sampling of points around *Γ*.

We visualize the exciton wavefunctions by placing the hole in the center of a 2 × 2 × 6 unit cell. To visually assess the trend in spatial localization when considering EPC, we plot the exciton coefficients in the different directions (*k*_*x*_, *k*_*y*_, *k*_*z*_) for undisplaced and displaced configurations, as previously reported for molecular crystals^27^. Then, to compute the direction-dependent spread for each case, we followed the steps: 1. Using the plotxct.x functionality, we plot the electron-hole wavefunctions (see Equation (4)) with the hole fixed at 20 different linker-based C and O atoms close to the geometric center of the unit cell, for the static (undisplaced) configurations. Our focus on linker atoms as potential hole positions is due to the fact that the linker contribution to the valence states is predominant (C(p) and O(p) orbitals in Fig. S25), and justified by the high hole probabilities computed using the electron-hole correlation function *g(r)* (Equation (S5), see SI Section 14)^66^. With this step, we established the O atom close to the center of the unit cell as a high-probability site. From this stage onward, we fix the hole at this atom. 2. Then, we compute the planar averaged electron-hole correlation function, which provides a profile of the probability of finding an electron given a fixed hole position (see SI Section 14 and Equation (S5)). These quantities are plotted in Figs. 2 and 3b, c, and they represent the probability density in each direction (for Figs. 2 and 3b, c, the hole position is fixed at the chosen O). More details are available in the Supplementary Information Section 14. 3. Finally, we compute the standard deviation of the planar averaged electron-hole correlation function in each direction and refer to this value as the direction-dependent spread, where higher values indicate a higher degree of delocalization in that direction. In the z direction, for example, the spread would follow Equation (5).

4 $$\Psi ({r}_{{{\rm{e}}}},{r}_{{{\rm{h}}}})=\mathop{\sum}\limits_{k,c,v}{A}_{vck}^{S}\,{\psi }_{k,c}({r}_{{{\rm{e}}}}){\psi }_{k,v}^{*}({r}_{{{\rm{h}}}})\qquad$$

5 $${\sigma }_{z}=\sqrt{\int\,{{\rm{d}}}{r}_{\parallel }\,{\left({r}_{\parallel }-\langle {r}_{\parallel }\rangle \right)}^{2}\,g({r}_{\parallel })},\qquad \langle {r}_{\parallel }\rangle=\int\,{{\rm{d}}}{r}_{\parallel }\,{r}_{\parallel }\,g({r}_{\parallel }),$$

Lastly, for a better comparison with experimental UV-Vis spectra, we compute the absorption coefficient and absorbance value from the *G*_0_*W*_0_-BSE dielectric function. Following previous studies^67,68^, the absorption coefficient *α*(*ω*) is given by

6 $${\alpha }_{{{\rm{3D}}}}(\omega )=\frac{2\omega }{c}\sqrt{\frac{1}{2}\left[-{\epsilon }_{1}(\omega )+\sqrt{{\epsilon }_{1}{(\omega )}^{2}+{\epsilon }_{2}{(\omega )}^{2}}\right]}$$

where *ϵ*_1_(*ω*) and *ϵ*_2_(*ω*) are the real and imaginary part of the dielectric function, respectively.

Then, the absorbance is computed as

7 $${{\rm{Abs}}}(\omega )=1-\exp \left[-\alpha (\omega ){L}_{z}\right]$$

where *L*_*z*_ is taken as the length of the cell along the *z* direction.

### Experimental methods

#### SU-101 synthesis

The synthesis of SU-101 was reproduced from a reported procedure^37^. Briefly, ellagic acid (0.5 mmol, 0.15 g) and bismuth acetate (1 mmol, 0.38 g) were added to a water and acetic acid mixture (30 mL, 6 vol.% glacial acetic acid) in a round-bottom flask. The suspension was then stirred at room temperature for 48 hours. After the reaction, the MOF was recovered with a yield of 75% by centrifugation and washed with water. The sample was left to dry overnight in an oven at 60 °C.

#### Powder X-ray diffraction (PXRD)

PXRD patterns were collected in transmission mode with a Bruker D8 Discover system with a Cu K*α* source (1.54056 Å) at 40 kV and 40 mA. The scanning range was 2–40^∘^ (2*θ*) at a scanning rate of 4 seconds per step and a 2*θ* step of 0.02°. The activated sample was degassed ex-situ in a 0.5 mm capillary under dynamic vacuum in a sand bath at 150 °C overnight. Simulated PXRD patterns were generated from the corresponding crystal structures using Mercury 3.0. Indexing and Le Bail fits were performed using the TOPAS-academic *v6* software package^69^.

#### Nitrogen (N_2_) adsorption isotherms at 77 K

Isotherms were performed with a BELSORP Mini (BEL Japan, Inc.). Before measurements, the samples were activated under various activation conditions in a dynamic vacuum using a Belprep II (BEL Japan, Inc.) activation station. A part of the N_2_ adsorption isotherm in the *p*/*p*_0_ range 0.06–0.25 was fitted to the Brunauer-Emmett-Teller (BET) equation to estimate the surface areas of the samples. The pore volumes were also determined at *p*/*p*_0_ = 0.5.

#### Fourier transform infrared spectroscopy (FT-IR)

FT-IR measurements were performed with a Bruker ALPHA FT-IR spectrometer in transmittance mode from 400 to 4000 cm^−1^. The measurements were performed in a glove box to avoid exposing the samples to air.

#### Thermogravimetric analysis (TGA)

The PerkinElmer TG instrument was used for TGA measurement. The measurement was performed under airflow from room temperature up to 700 °C, starting at 30 °C, with a heating rate of 5 K/min.

#### Ultraviolet-visible

UV-Vis absorption spectra were obtained using a PerkinElmer Lambda 950 S UV/Vis/NIR Spectrometer equipped with an integrating sphere. The samples were suspended in cyclohexane (~1.24 μmol in 3 mL) in a quartz air-tight cuvette. This concentration was chosen to ensure sufficient signal intensity while maintaining adequate light transmission through the suspension. We note that the spectral features are not sensitive to variations in concentration within this regime; the reported value is provided for completeness and reproducibility.

#### Steady-state PL

PL spectra were recorded with a Horiba Jobin Vyon Fluorolog-3 instrument. For PL measurements, the excitation wavelength was 330 nm. Both measurements were performed in MOF suspensions of cyclohexane (~1.24 μmol in 3 ml) in an airtight quartz cuvette.

#### Time-resolved fluorescence lifetimes (TRPLs)

TRPLs of SU-101 were measured on a Horiba Jobin Yvon Fluorolog-3 equipped with a photomultiplier tube. Samples of as-synthesized and activated MOF were homogeneously suspended in degassed cyclohexane (1.25 μmol in 3 mL) in air-tight quartz cuvettes, and excited using a Horiba nanoLED-370 with a 369 nm excitation wavelength, 1.3 ns pulse duration, and a repetition rate of 100 kHz.

## Supplementary information

## Data Availability

All data generated in this study have been deposited in the Zenodo database under accession code https://doi.org/10.5281/zenodo.18415008. All analyses are provided in the Supplementary Information file. Relevant phonon modes for the exciton–phonon coupling explored in this study are provided in the Supplementary Information.

## References

[1] Yaghi, O. M. et al. Reticular synthesis and the design of new materials. *Nature* **423**, 705–714 (2003). 12802325 10.1038/nature01650

[2] O’Keeffe, M. & Yaghi, O. M. Deconstructing the crystal structures of metal–organic frameworks and related materials into their underlying nets. *Chem. Rev.* **112**, 675–702 (2011). 21916513 10.1021/cr200205j

[3] Charalambous, C. et al. A holistic platform for accelerating sorbent-based carbon capture. *Nature* **632**, 89–94 (2024). 39020168 10.1038/s41586-024-07683-8 PMC11291289

[4] P. Domingues, N. et al. Unraveling metal effects on co2 uptake in pyrene-based metal-organic frameworks. *Nat. Commun.* **16**, 10.1038/s41467-025-56296-w (2025). PMC11814143 39934127

[5] Furukawa, H., Cordova, K. E., O’Keeffe, M. & Yaghi, O. M. The chemistry and applications of metal-organic frameworks. *Science* **341**, 10.1126/science.1230444 (2013). 23990564

[6] Dhakshinamoorthy, A., Asiri, A. M. & García, H. Metal–organic framework (MOF) compounds: Photocatalysts for redox reactions and solar fuel production. *Angew. Chem. Int. Ed.* **55**, 5414–5445 (2016). 10.1002/anie.201505581 26970539

[7] Ortega-Guerrero, A., Fumanal, M., Capano, G., Tavernelli, I. & Smit, B. Insights into the electronic properties and charge transfer mechanism of a porphyrin ruthenium-based metal–organic framework. *Chem. Mater.* **32**, 4194–4204 (2020).

[8] Fumanal, M., Ortega-Guerrero, A., Jablonka, K. M., Smit, B. & Tavernelli, I. Charge separation and charge carrier mobility in photocatalytic metal-organic frameworks. *Adv. Funct. Mater.* **30**, 2003792 (2020).

[9] Liu, H., Xu, C., Li, D. & Jiang, H. Photocatalytic hydrogen production coupled with selective benzylamine oxidation over mof composites. *Angew. Chem.* **130**, 5477–5481 (2018). 10.1002/anie.201800320 29508919

[10] Zhang, Y. et al. Tunable chiral metal organic frameworks toward visible light–driven asymmetric catalysis. *Sci. Adv.* **3**, 10.1126/sciadv.1701162 (2017). PMC5562422 28835929

[11] Stanley, P. M., Ramm, V., Fischer, R. A. & Warnan, J. Analysis of metal–organic framework-based photosynthetic co2 reduction. *Nat. Synth.* **3**, 307–318 (2024).

[12] Zhang, M. et al. Two pure mof-photocatalysts readily prepared for the degradation of methylene blue dye under visible light. *Dalton Trans.* **47**, 4251–4258 (2018). 29485653 10.1039/c8dt00156a

[13] Fumanal, M., Capano, G., Barthel, S., Smit, B. & Tavernelli, I. Energy-based descriptors for photo-catalytically active metal-organic framework discovery. *J. Mater. Chem. A* **8**, 4473–4482 (2020).

[14] Alzard, R. H., Siddig, L. A., S Abdelhamid, A. & Alzamly, A. Visible-light-driven photocatalytic coupling of neat benzylamine over a bi-ellagate metal–organic framework. *ACS Omega* **7**, 36689–36696 (2022). 36278051 10.1021/acsomega.2c04934 PMC9583343

[15] Sanati, S., Varma, R. S., Liu, M. & Abazari, R. Non-noble metal–organic frameworks in electrocatalytic urea-assisted hydrogen production and energy-saving regeneration. *Energy Environ. Sci.* **18**, 7733–7755 (2025).

[16] Rieth, A. J., Wright, A. M. & Dincă, M. Kinetic stability of metal–organic frameworks for corrosive and coordinating gas capture. *Nat. Rev. Mater.* **4**, 708–725 (2019).

[17] Kökçam-Demir, U. et al. Coordinatively unsaturated metal sites (open metal sites) in metal–organic frameworks: design and applications. *Chem. Soc. Rev.* **49**, 2751–2798 (2020). 32236174 10.1039/c9cs00609e

[18] Yadav, A. et al. Open metal site (oms)-inspired investigation of adsorption and catalytic functions in a porous metal–organic framework (mof). *Dalton Trans.* **51**, 15496–15506 (2022). 36164811 10.1039/d2dt02098j

[19] Hu, Y. et al. A dual-purpose ce(iii)–organic framework with amine groups and open metal sites: third-order nonlinear optical activity and catalytic co2 fixation. *ACS Appl. Mater. Interfaces* **15**, 37300–37311 (2023). 37497576 10.1021/acsami.3c04506

[20] Shustova, N. B., Cozzolino, A. F., Reineke, S., Baldo, M. & Dincă, M. Selective turn-on ammonia sensing enabled by high-temperature fluorescence in metal–organic frameworks with open metal sites. *J. Am. Chem. Soc.* **135**, 13326–13329 (2013). 23981174 10.1021/ja407778a

[21] Chen, B. et al. Luminescent open metal sites within a metal–organic framework for sensing small molecules. *Adv. Mater.* **19**, 1693–1696 (2007).

[22] Liang, W. Y. Excitons. *Phys. Educ.* **5**, 226–228 (1970).

[23] van der Heide, T., Hourahine, B., Aradi, B., Frauenheim, T. & Niehaus, T. A. Phonon-induced band gap renormalization by dielectric dependent global hybrid density functional tight binding. *Phys. Rev. B* **109**, 10.1103/PhysRevB.109.245103 (2024).

[24] Rangel, T. et al. Structural and excited-state properties of oligoacene crystals from first principles. *Phys. Rev. B* **93**, 10.1103/PhysRevB.93.115206 (2016).

[25] Brown-Altvater, F. et al. Band gap renormalization, carrier mobilities, and the electron-phonon self-energy in crystalline naphthalene. *Phys. Rev. B* **101**, 10.1103/PhysRevB.101.165102 (2020).

[26] Alvertis, A. M. & Engel, E. A. Importance of vibrational anharmonicity for electron-phonon coupling in molecular crystals. *Phys. Rev. B* **105**, 10.1103/PhysRevB.105.L180301 (2022).

[27] Alvertis, A. M., Haber, J. B., Engel, E. A., Sharifzadeh, S. & Neaton, J. B. Phonon-induced localization of excitons in molecular crystals from first principles. *Phys. Rev. Lett.* **130**, 10.1103/PhysRevLett.130.086401 (2023). 36898125

[28] Ghosh, P. et al. Decoupling excitons from high-frequency vibrations in organic molecules. *Nature* **629**, 355–362 (2024). 38720042 10.1038/s41586-024-07246-x PMC11078737

[29] Kshirsagar, A. R., Blase, X., Attaccalite, C. & Poloni, R. Strongly bound excitons in metal–organic framework mof-5: a many-body perturbation theory study. * J. Phys. Chem. Lett.* **12**, 4045–4051 (2021). 33881873 10.1021/acs.jpclett.1c00543

[30] Lee, J.-H., Lee, S.-H. & Son, Y.-W. Benchmarking first-principles approaches for the band gap prediction of nanoporous materials. *J. Chem. Theory Comput.* **21**, 6922–6932 (2025). 40583334 10.1021/acs.jctc.5c00818

[31] Chen, D. et al. Ultrastrong electron-phonon coupling in uranium-organic frameworks leading to inverse luminescence temperature dependence. *Angew. Chem. Int. Ed.* **63**, 10.1002/anie.202318559 (2024). 38153004

[32] Ma, W., Song, X., Yin, J. & Fei, H. Intrinsic self-trapped broadband emission from zinc halide-based metal–organic frameworks. *Chem. Commun.* **57**, 1396–1399 (2021). 10.1039/d0cc07320b 33438705

[33] Alvertis, A. M., Williams-Young, D. B., Bruneval, F. & Neaton, J. B. Influence of electronic correlations on electron–phonon interactions of molecular systems with the *GW* and coupled cluster methods. *J. Chem. Theory Comput.* **20**, 6175–6183 (2024). 38954597 10.1021/acs.jctc.4c00327

[34] Feng, S. et al. Tunable self-trapped excitons in 2d layered rubrene. *Appl. Phys. Lett.* **118**, 10.1063/5.0049942 (2021).

[35] Filip, M. R., Haber, J. B. & Neaton, J. B. Phonon screening of excitons in semiconductors: Halide perovskites and beyond. *Phys. Rev. Lett.* **127**, 10.1103/PhysRevLett.127.067401 (2021). 34420331

[36] Alvertis, A. M. et al. Phonon screening and dissociation of excitons at finite temperatures from first principles. *Proc. Natl. Acad. Sci.* **121**, 10.1073/pnas.2403434121 (2024). PMC11287246 39024110

[37] Grape, E. S. et al. A robust and biocompatible bismuth ellagate MOF synthesized under green ambient conditions. *J. Am. Chem. Soc.* **142**, 16795–16804 (2020). 32894014 10.1021/jacs.0c07525 PMC7586326

[38] Chacón-García, A. J. et al. Su-101 for the removal of pharmaceutical active compounds by the combination of adsorption/photocatalytic processes. *Sci. Rep.* **14**, 10.1038/s41598-024-58014-w (2024). PMC10991395 38570568

[39] Obeso, J. L. et al. Lewis acid-catalyzed ring-opening alcoholysis of cyclohexene oxide: the role of open metal sites in the bi(iii)-based metal-organic framework su-101. *ChemCatChem* **15**, 10.1002/cctc.202300471 (2023).

[40] Zhang, Q. et al. A biocompatible bismuth based metal-organic framework as efficient light-sensitive drug carrier. *J. Colloid Interface Sci.* **617**, 578–584 (2022). 35303641 10.1016/j.jcis.2022.01.188

[41] Monserrat, B. Electron–phonon coupling from finite differences. *J. Phys. Condens. Matter* **30**, 083001 (2018). 29328057 10.1088/1361-648X/aaa737

[42] Perdew, J. P., Burke, K. & Ernzerhof, M. Generalized gradient approximation made simple. *Phys. Rev. Lett.* **77**, 3865–3868 (1996). 10062328 10.1103/PhysRevLett.77.3865

[43] Deslippe, J. et al. Berkeleygw: a massively parallel computer package for the calculation of the quasiparticle and optical properties of materials and nanostructures. *Comput. Phys. Commun.* **183**, 1269–1289 (2012).

[44] Hybertsen, M. S. & Louie, S. G. Electron correlation in semiconductors and insulators: band gaps and quasiparticle energies. *Phys. Rev. B* **34**, 5390–5413 (1986). 10.1103/physrevb.34.5390 9940372

[45] Rohlfing, M. & Louie, S. G. Electron-hole excitations and optical spectra from first principles. *Phys. Rev. B* **62**, 4927–4944 (2000).

[46] Mourino, B., Jablonka, K. M., Ortega-Guerrero, A. & Smit, B. In search of covalent organic framework photocatalysts: a dft-based screening approach. *Adv. Funct. Mater.* **n/a**, 2301594 (2023).

[47] Warmbier, R., Quandt, A. & Seifert, G. Dielectric properties of selected metal–organic frameworks. * J. Phys. Chem. C.* **118**, 11799–11805 (2014).

[48] Giustino, F., Louie, S. G. & Cohen, M. L. Electron-phonon renormalization of the direct band gap of diamond. *Phys. Rev. Lett.* **105**, 10.1103/PhysRevLett.105.265501 (2010). 21231677

[49] Lugovskoi, A., Katsnelson, M. & Rudenko, A. Strong electron-phonon coupling and its influence on the transport and optical properties of hole-doped single-layer inse. *Phys. Rev. Lett*. **123**, 10.1103/PhysRevLett.123.176401 (2019). 31702262

[50] Cardona, M. Renormalization of the optical response of semiconductors by electron-phonon interaction. *Phys. Status Solidi* **188**, 1209–1232 (2001).

[51] Kentsch, R. et al. Exciton dynamics and electron–phonon coupling affect the photovoltaic performance of the cs2agbibr6 double perovskite. * J. Phys. Chem. C.* **122**, 25940–25947 (2018).

[52] Jiang, Y. et al. Suppressing electron-phonon coupling in organic photovoltaics for high-efficiency power conversion. *Nat. Commun.* **14**, 10.1038/s41467-023-40806-9 (2023). PMC10442373 37604923

[53] Zhang, Z. et al. Delocalizing excitation for highly-active organic photovoltaic catalysts. *Angew. Chem. Int. Ed*. 10.1002/ange.202402343 (2024). 38639055

[54] Miglio, A. et al. Predominance of non-adiabatic effects in zero-point renormalization of the electronic band gap. *npj Comput. Mater.* **6**, 10.1038/s41524-020-00434-z (2020).

[55] Monserrat, B. Vibrational averages along thermal lines. *Phys. Rev. B* **93**, 10.1103/PhysRevB.93.014302 (2016).

[56] Gant, S. E. et al. Ultrafast spontaneous exciton dissociation via phonon emission in BiVO 4. *Phys. Rev. Res.* **8**, 10.1103/xm7z-v1ks (2026).

[57] Giannozzi, P. et al. QUANTUM ESPRESSO: a modular and open-source software project for quantum simulations of materials. *J. Phys. Condens. Matter* **21**, 395502 (2009). 21832390 10.1088/0953-8984/21/39/395502

[58] Grimme, S., Antony, J., Ehrlich, S. & Krieg, H. A consistent and accurateab initioparametrization of density functional dispersion correction (dft-d) for the 94 elements h-pu. *J. Chem. Phys.* **132**, 10.1063/1.3382344 (2010). 20423165

[59] Marzari, N., Vanderbilt, D., De Vita, A. & Payne, M. C. Thermal contraction and disordering of the al(110) surface. *Phys. Rev. Lett.* **82**, 3296–3299 (1999).

[60] van Setten, M. et al. The pseudodojo: training and grading a 85 element optimized norm-conserving pseudopotential table. *Comput. Phys. Commun.* **226**, 39–54 (2018).

[61] Hamann, D. R. Optimized norm-conserving vanderbilt pseudopotentials. *Phys. Rev. B* **88**, 10.1103/PhysRevB.88.085117 (2013).

[62] Fennie, C. J. & Rabe, K. M. Structural and dielectric properties of Sr_2_TiO_4_ from first principles. *Phys. Rev. B* **68**, 10.1103/PhysRevB.68.184111 (2003).

[63] Qiu, D. Y., da Jornada, F. H. & Louie, S. G. Optical spectrum of mos_2_: many-body effects and diversity of exciton states. *Phys. Rev. Lett.* **111**, 10.1103/PhysRevLett.111.216805 (2013). 24313514

[64] Butler, K. T., Hendon, C. H. & Walsh, A. Electronic chemical potentials of porous metal–organic frameworks. *J. Am. Chem. Soc.* **136**, 2703–2706 (2014). 24447027 10.1021/ja4110073 PMC3946036

[65] Tamblyn, I., Darancet, P., Quek, S. Y., Bonev, S. A. & Neaton, J. B. Electronic energy level alignment at metal-molecule interfaces with a gw approach. *Phys. Rev. B* **84**, 10.1103/PhysRevB.84.201402 (2011).

[66] Sharifzadeh, S., Darancet, P., Kronik, L. & Neaton, J. B. Low-energy charge-transfer excitons in organic solids from first-principles: the case of pentacene. * J. Phys. Chem. Lett.* **4**, 2197–2201 (2013).

[67] Qiu, D. Y., da Jornada, F. H. & Louie, S. G. Environmental screening effects in 2d materials: renormalization of the bandgap, electronic structure, and optical spectra of few-layer black phosphorus. *Nano Lett.* **17**, 4706–4712 (2017). 28677398 10.1021/acs.nanolett.7b01365

[68] Champagne, A., Camarasa-Gómez, M., Ricci, F., Kronik, L. & Neaton, J. B. Strongly bound excitons and anisotropic linear absorption in monolayer graphullerene. *Nano Lett.* **24**, 7033–7039 (2024). 38805193 10.1021/acs.nanolett.4c01497

[69] Coelho, A. A. Topas and topas-academic: an optimization program integrating computer algebra and crystallographic objects written in C++. *J. Appl. Crystallogr.* **51**, 210–218 (2018).

