# Supplementary material for: Hydration-dependent exciton-phonon coupling in a metal-organic framework photocatalyst
Source: Nat Commun. 2026 Aug 5;17:9423. doi: 10.1038/s41467-026-76097-z (PMC13538642; doi:10.1038/s41467-026-76097-z)
Supplement: Supplementary file 1 [file 41467_2026_76097_MOESM1_ESM.pdf]

Supplementary Information: Hydration-dependent exciton-phonon coupling in a metal-organic framework photocatalyst

Beatriz Mouriño [1,2], Antonios Alvertis [3,4], Alex Smith [2,5,†], Nency P. Domingues [1], Fatmah Mish Ebrahim [1], Aurélie Champagne [2,6], Jeffrey Neaton [2,5,7,*], and Berend Smit [1,*]

[1] *Laboratory of Molecular Simulation (LSMO), Institut des Sciences et Ingénierie Chimiques, Valais Ecole Polytechnique Fédérale de Lausanne (EPFL), Rue de l'Industrie 17, CH-1951 Sion, Valais, Switzerland*

[2] *Materials Sciences Division, Lawrence Berkeley National Laboratory, Berkeley, CA 94720 USA*

[3] *Department of Physics, The University of Texas at Austin, Austin, TX 78712 USA*

[4] *Oden Institute for Computational Engineering and Sciences, The University of Texas at Austin, Austin, TX 78712 USA*

[5] *Department of Physics, University of California, Berkeley, Berkeley, CA 94720 USA*

[6] *Université de Bordeaux, CNRS, Bordeaux INP, ICMCB, UMR 5026, Pessac, F-33600, France*

[7] *Kavli Energy Nanosciences Institute at Berkeley, Berkeley, CA 94720 USA*

[*] *Corresponding email: berend.smit@epfl.ch, jbneaton@lbl.gov*

[†] Present address: Department of Chemistry, Columbia University, New York, New York 10027, United States

Contents

1 Extended background on SU-101 **S4**

2 Structural properties **S4**

2.1 Synthesized SU-101 . . . . S4

2.2 Band structures (no phonons) S6

3 DFT descriptors for photocatalysis **S7**

4 Anisotropy in absorption spectra **S8**

5 Absorption spectra without phonons **S9**

6 Phonons **S10**

6.1 Anharmonicity tests S10

6.2 Monte-Carlo sampling of vibrational averages S11

6.3 Atomic contribution S12

6.4 Mode contribution S14

6.5 Phonon-driven renormalization of band gaps S16

7 Optical Properties Measurements **S17**

8 Absorption spectra with phonons **S22**

9 Exciton coefficients **S24**

10 Spin-orbit coupling **S26**

10.1 Potential energy surface S26

10.2 Band structures S27

11 GW-BSE convergence tests **S30**

11.1 GW S30

11.2 Perturbative approach for including SOC S30

11.3 BSE S32

11.4 Displaced configurations S33

12 Band alignment **S34**

13 Averaged predicted optoelectronic properties **S35**

13.1 Absorption S35

14 Planar-averaged electron-hole correlation function **S37**

14.1 Planar-averaged electron-hole correlation function at 0 K and 300 K S38

15 Experimental Discussion **S40**

1 Extended background on SU-101

Bismuth-ellagate (SU-101) was first synthesized to target biocompatibility, which explains the choice of the nontoxic metal Bi^{3+} and the bioactive organic linker, ellagic acid [1]. SU-101 has tetragonal symmetry and an **svd** net topology [1], with metal nodes arranging in "channels" in a rod-like shape, *i.e.* Bi-oxo clusters connected along a single direction. In addition to low toxicity, low production cost, and thermal and chemical stability, the chosen building blocks also offer interesting properties for light-related applications. Bi-based materials, such as bismuth vanadate, are largely studied as photocatalysts [2–4]. While Bi^{3+} has a filled d-shell that is less accessible compared to transition metals, the photocatalytic activity and optoelectronic properties of Bi-containing materials can be modulated by tuning their coordination environments [5–8].

In SU-101, the Bi open metal sites (OMS) are present after removal of coordinated water (activation), and can act as Lewis acid electron acceptor sites [5]. Ellagic acid, in contrast, has an electron-rich π-conjugated system [9] and offers a platform for hydrogen bonding [10], further enhancing the material's catalytic potential. Recent studies have demonstrated SU-101's effectiveness as a photocatalyst for the coupling of benzylamine and the removal of pharmaceutical active compounds [11, 12]. Both studies indicate that SU-101 absorbs light in the visible range, a critical feature for photocatalysts.

2 Structural properties

2.1 Synthesized SU-101

The success of the synthesis is evaluated by powder X-ray diffraction (PXRD). The obtained PXRD pattern of the as-made material is in full agreement with the single-crystal derived XRD from the original reported crystallographic information file (CIFs). Fitting the PXRD data of the as-made SU-101 according to the Le Bail method yields refined cell parameters a

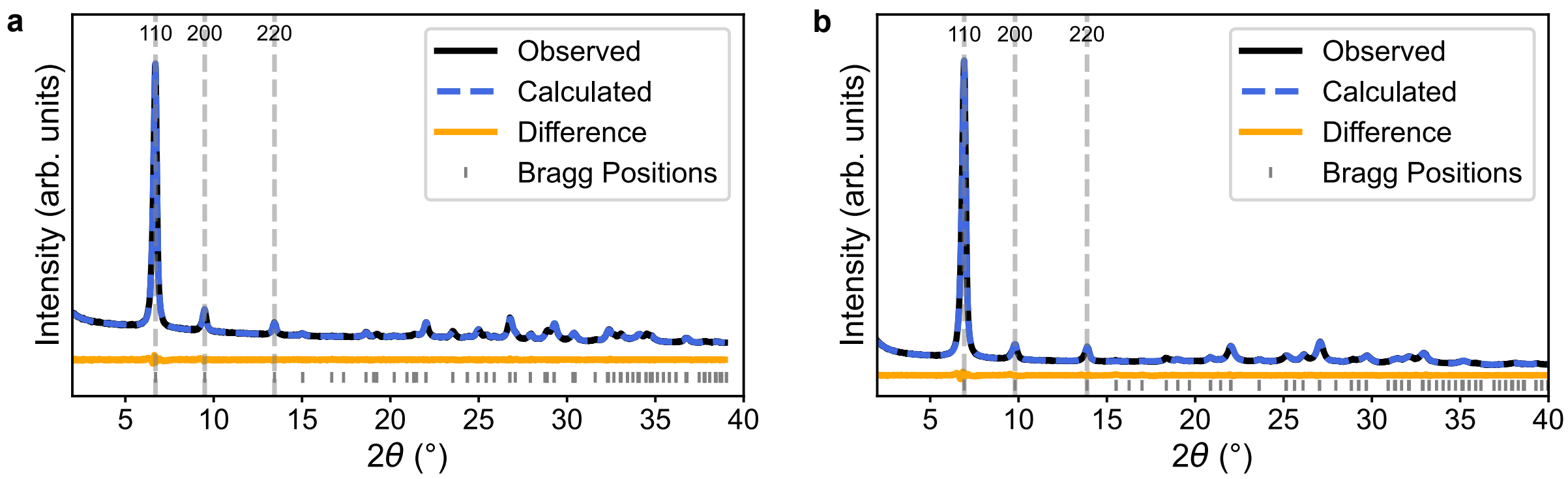

Figure S1: X-ray powder diffraction profile for the Le Bail fits of **a.** the as-made material. The unit cell parameters obtained are: $a = b = 18.61(7)$, $c = 5.54(1)$, $P4_2/n$ ($R_{wp} = 2.18$ %). **b.** the activated material (*ex-situ* activation at 150 °C under dynamic vacuum), with $a = b = 18.03(7)$, $c = 5.71(4)$, $P4_2/n$ ($R_{wp} = 2.53$ %,). Reflection positions are also marked.

$= b = 18.61(7)$ Å, $c = 5.54(1)$ Åwith a good fit indicator ($R_{wp} = 2.18$ %) consistent with those obtained from the 3D electron diffraction (3DED) structure determination provided in the original publication ($a = b = 18.44$ Å, $c = 5.82$ Å) [1]. The stability of the structure is also evaluated after activation at 150 °C for 12 hours, showing still a crystalline powder. Upon removal of the coordinated water, the Le Bail fit of the activated material yields slightly different unit cell parameters $a = b = 18.03(7)$ Å, $c = 5.71(4)$ Å($R_{wp} = 2.53$ %) compared to the as-made. We observe a shrinking of a and b and expansion of the cell along c, which is in agreement with the predictions from the optimized CIFs.

The assignment of the lattice parameter changes (i.e., contraction in a and b, and expansion along c) is not based on the shift of a single diffraction peak, but rather on full-pattern analysis via Le Bail refinement of the PXRD data. The first low-angle peak in Figure 1a corresponds to the (110) reflection. Its shift to higher 2θ upon activation indicates a reduction in the corresponding d-spacing, and reflects a contraction in the ab plane. As reported in Figure S1, the refined unit cell parameters show a decrease in a and b and a concomitant increase in c upon activation. The (110) reflection alone does not provide direct information about changes along the c-axis.

2.2 Band structures (no phonons)

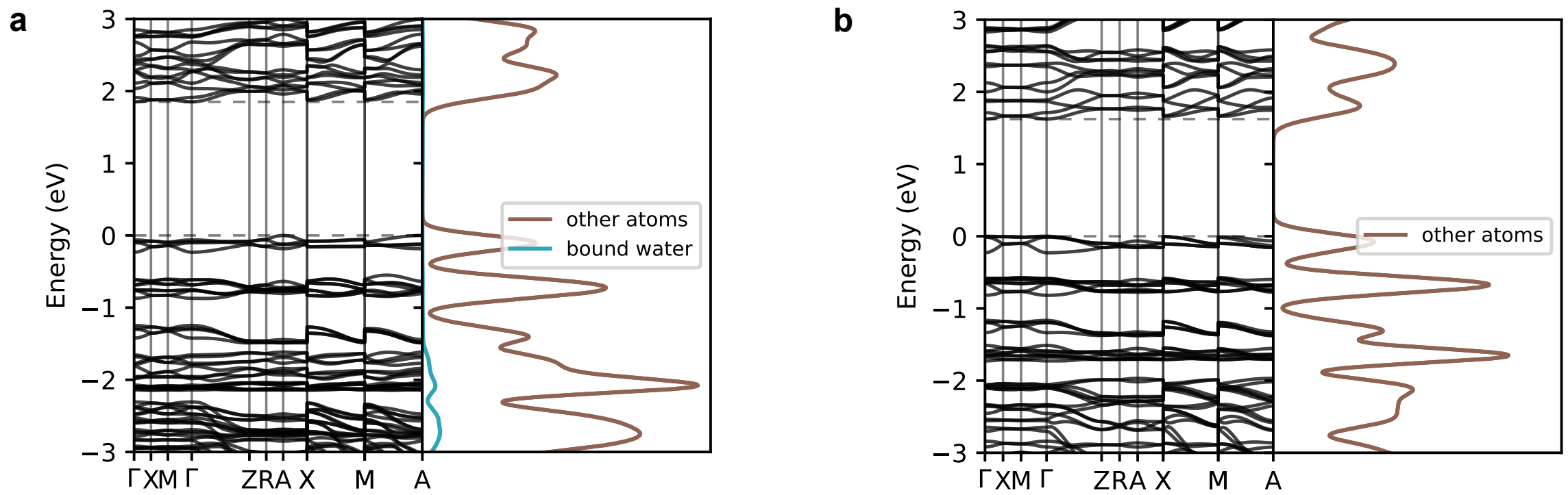

Figure S2: PBE band structure (with SOC) for a) bare, b) water bound SU-101. The projected DOS of the bound water Os are indicated in blue.

3 DFT descriptors for photocatalysis

Given the interest in SU-101 for different photocatalytic applications [11–13], we also employed a DFT-based workflow [14] to compute photocatalytic descriptors. We preliminarly assessed the SU-101 MOF for photocatalysis via a dedicated AiiDA workflow [14–16] that utilizes the CP2K [17] DFT code and post-processing python packages [18–20] to compute band gap, band edge alignment, electron and hole effective masses and averaged spatial overlap between frontier orbitals of electron and hole injection (charged doublets). The most intriguing finding was that, upon water removal, the charge separation descriptor (Λ, defined as the averaged spatial overlap of charged doublets [21]) changes. For different water loadings, we computed the band gap, band alignment [19, 22], and a metric for charge separation (Λ) [21]. Λ is defined as the averaged spatial overlap of charged doublets (electron and hole injection). Lower values of Λ indicate more spatially separated charges. We notice a shift of the band alignment to more positive values relative to vacuum as water loading increases, as shown in Figure S34d. The computed charge separation descriptors (Λ) were 0.45 for the activated MOF, and 0.70 for the bound-water (50%) case. At the DFT level, the removal of bound water seems to strongly affect Λ (reduction from 0.7 to 0.45), indicating that it could lead to more separated excited electron and hole.

4 Anisotropy in absorption spectra

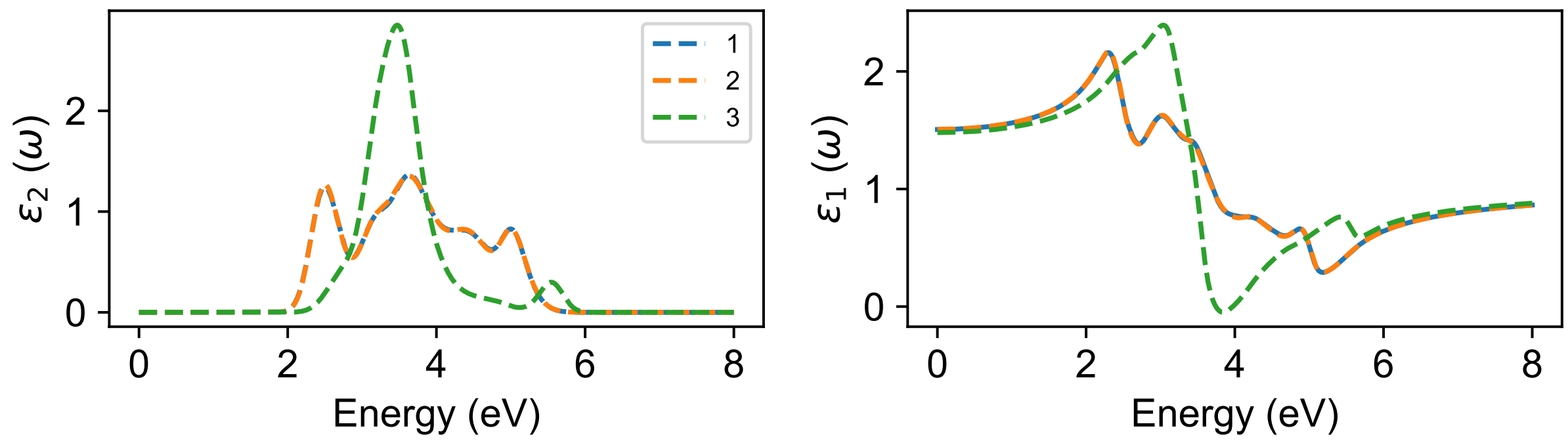

Figure S3: Absorption spectra for activated SU-101 (no phonons) computed with G_0W_0-BSE using the momentum operator, with polarization along different crystallographic directions. Direction 3 ([001]) is the direction of the rod-shaped metal node, and 1 and 2 comprise the perpendicular plane ([100] and [010], respectively).

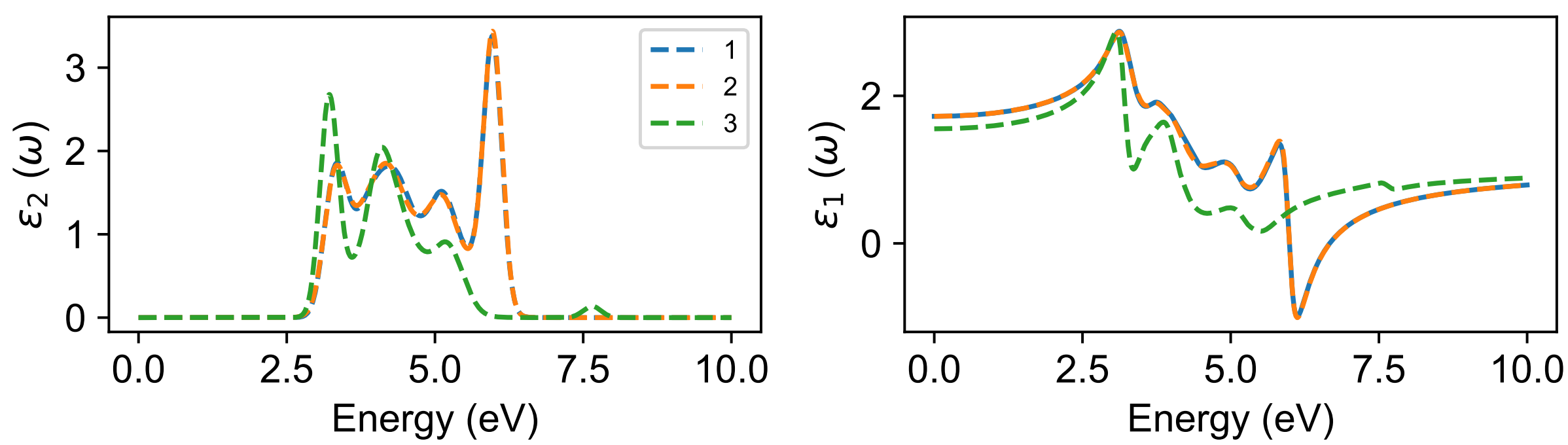

Figure S4: Absorption spectra for SU-101 (no phonons) with coordinated water (as-made) computed with G_0W_0-BSE using the momentum operator, with polarization along different crystallographic directions. Direction 3 ([001]) is the direction of the rod-shaped metal node, and 1 and 2 comprise the perpendicular plane ([100] and [010], respectively).

5 Absorption spectra without phonons

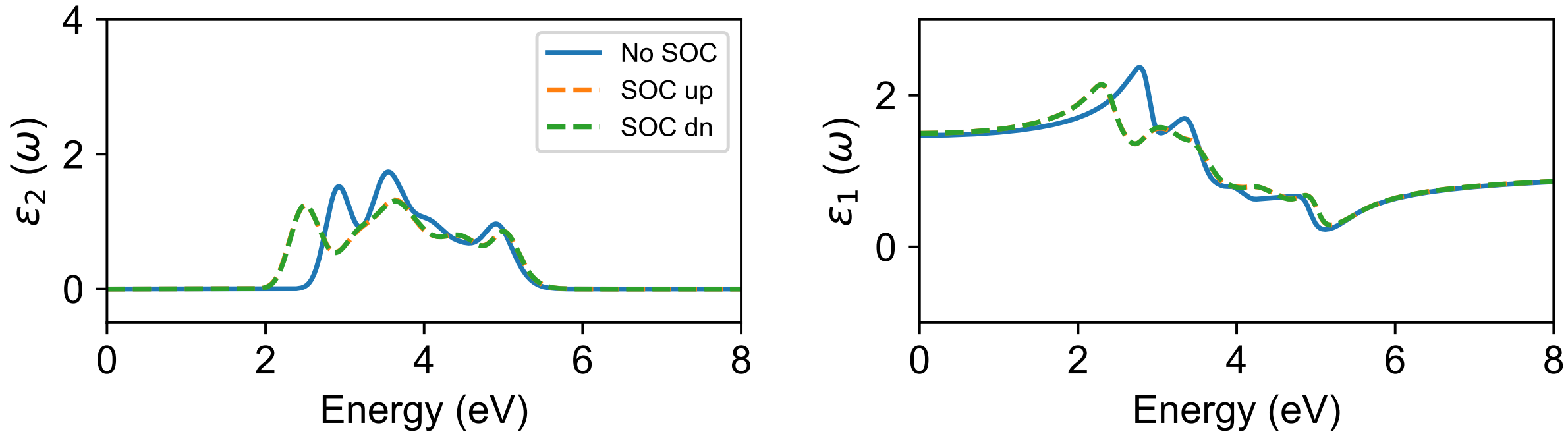

Figure S5: Computed absorption spectra using G_0W_0-BSE for the activated SU-101 (no phonons).

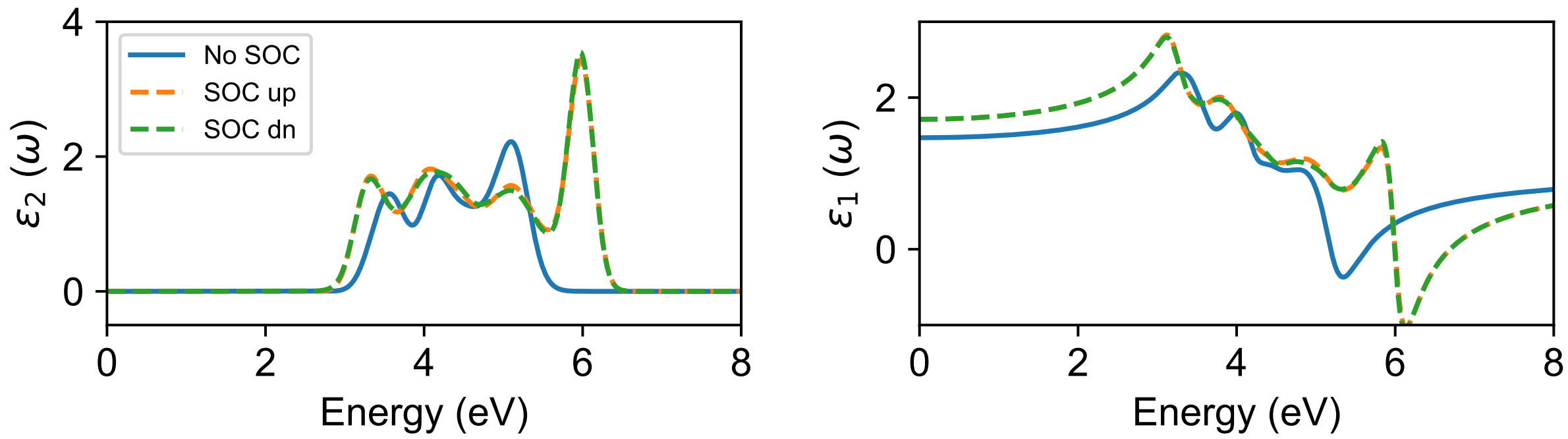

Figure S6: Computed absorption spectra using G_0W_0-BSE for SU-101 (no phonons) with coordinated water (as-made).

6 Phonons

6.1 Anharmonicity tests

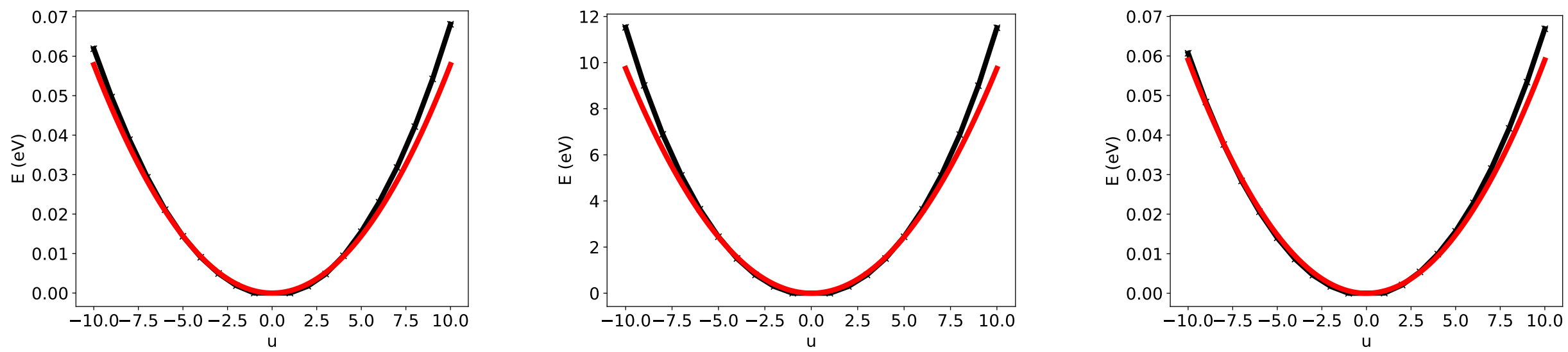

Figure S7: Comparing harmonic (red) and anharmonic (black) potential energy surfaces for a. a low-frequency ($\approx 5\,\mathrm{cm}^{-1}$, no SOC), b. a high-frequency ($\approx 1340\,\mathrm{cm}^{-1}$, no SOC), c. and a low-frequency ($\approx 19\,\mathrm{cm}^{-1}$, with SOC) phonon mode. The harmonic PES is fitted assuming perfect harmonic behavior given the phonon frequency. The phonon displacement u is given in units of the zero-point width $\frac{1}{\sqrt{2\omega}}$

6.2 Monte-Carlo sampling of vibrational averages

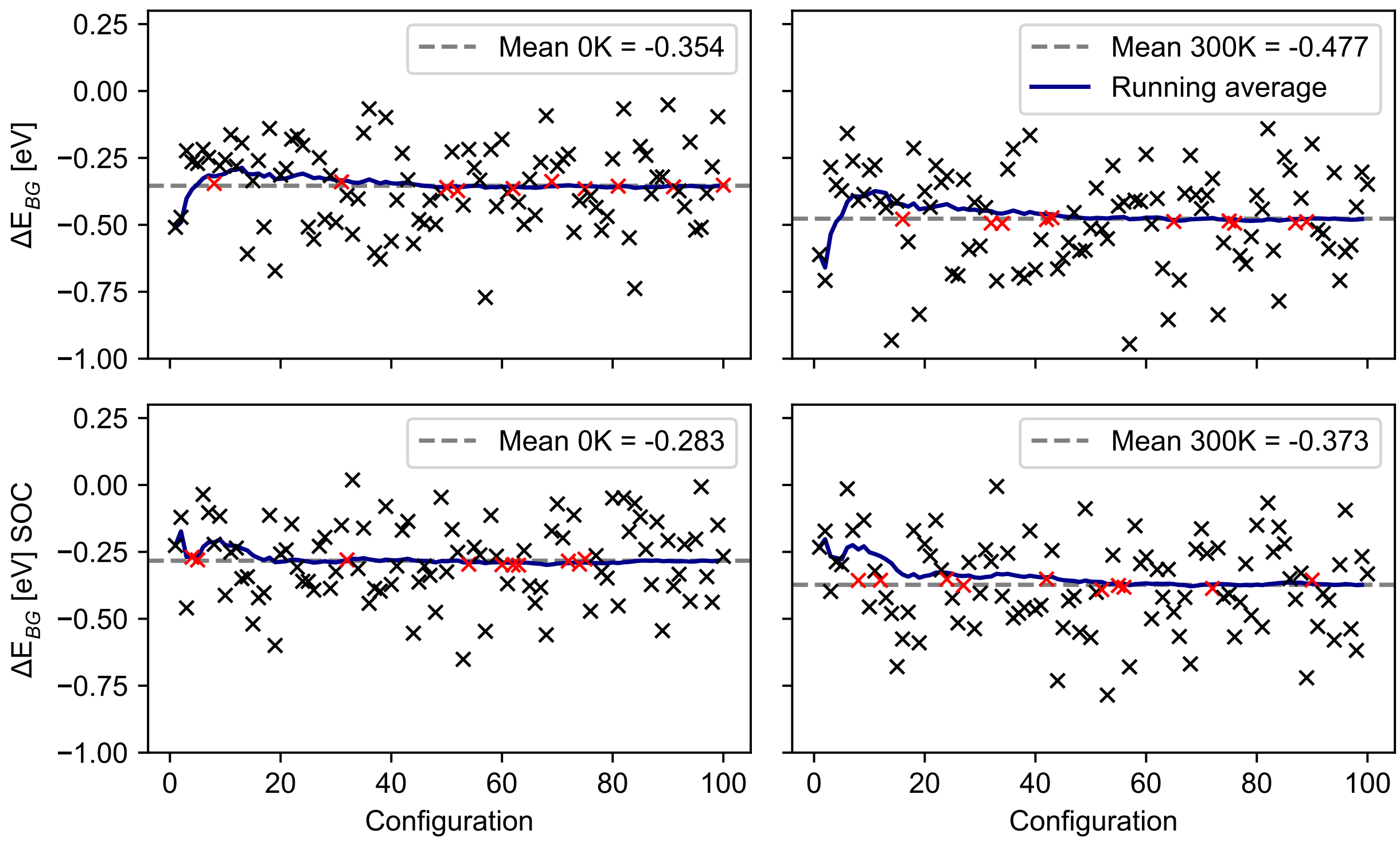

Figure S8: Convergence of the PBE gap renormalization of activated SU-101, with and without spin-orbit coupling, for 100 configurations at 0 K and 300 K. The dashed line is the mean, and the blue line is the running average. Red marks indicate 10 configurations that capture the mean effect at the evaluated temperature.

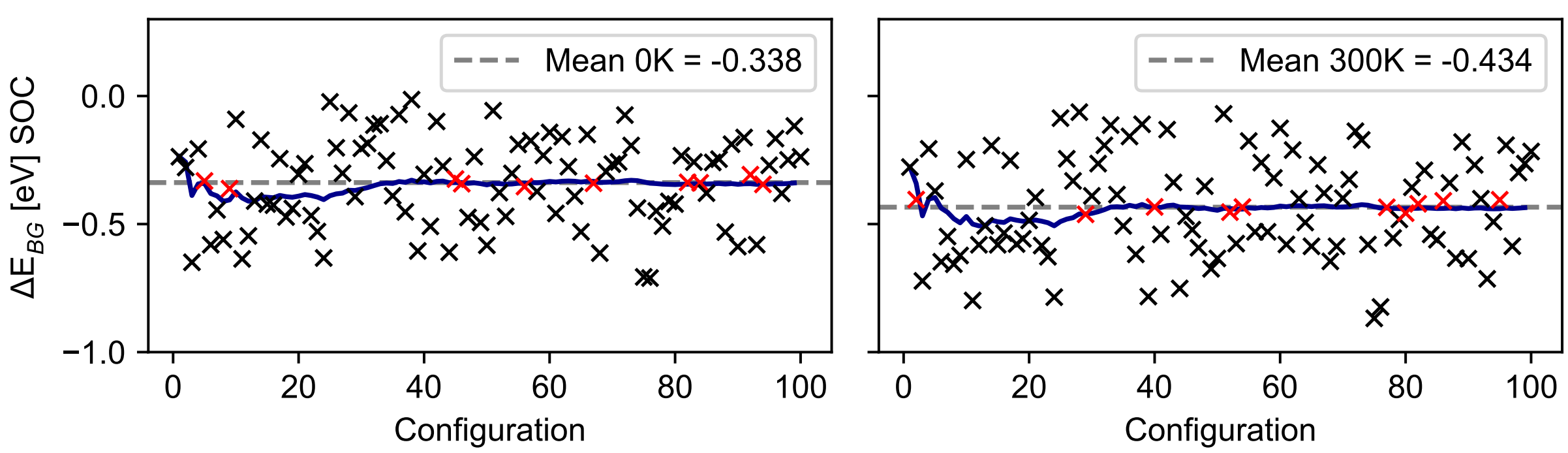

Figure S9: Convergence of the PBE gap renormalization of water-bound SU-101 with spin-orbit coupling, for 100 configurations at 0 K and 300 K.

6.3 Atomic contribution

To gain insight into the atomic origin of the temperature-dependent effects, we analyzed representative Monte Carlo (MC) configurations generated from phonon sampling. In these configurations, atomic displacements arise from linear combinations of phonon modes. For each system (activated and hydrated) and temperature (0 K and 300 K), we sampled 100 MC configurations. From these, 10 configurations were selected as representative for the GW/BSE calculations, based on their proximity to the average phonon-induced band gap renormalization. These configurations therefore capture the typical structural distortions associated with the thermal average. Thus, we analyze the atomic contribution based on these 10 configurations for each temperature and case.

For each configuration, atomic displacements were computed with respect to the corresponding undisplaced structure using periodic boundary conditions. The magnitude of the displacement was evaluated for every atom, and averaged over the 10 representative configurations. This analysis yielded per-atom measures of (i) mean displacement at 0 K, (ii) mean displacement at 300 K, and (iii) the change in displacement between 0 K and 300 K.

To compare the activated and hydrated systems, a one-to-one correspondence between the atoms of activated and hydrated systems was established using element matching and minimum-distance criteria in fractional coordinates. The additional atoms in the hydrated structure (coordinated water molecules) were identified as unmatched species.

From this analysis, the atoms with the largest displacements at each temperature were identified. By comparing the corresponding atoms across the two systems, it was possible to distinguish atoms that are commonly important in both structures from those whose impact on electron-phonon coupling is specific to either the activated or hydrated case. This enables direct identification of the atoms (e.g., metals, lactone oxygens, coordinated water) that dominate the gap renormalization and changes in excitonic properties.

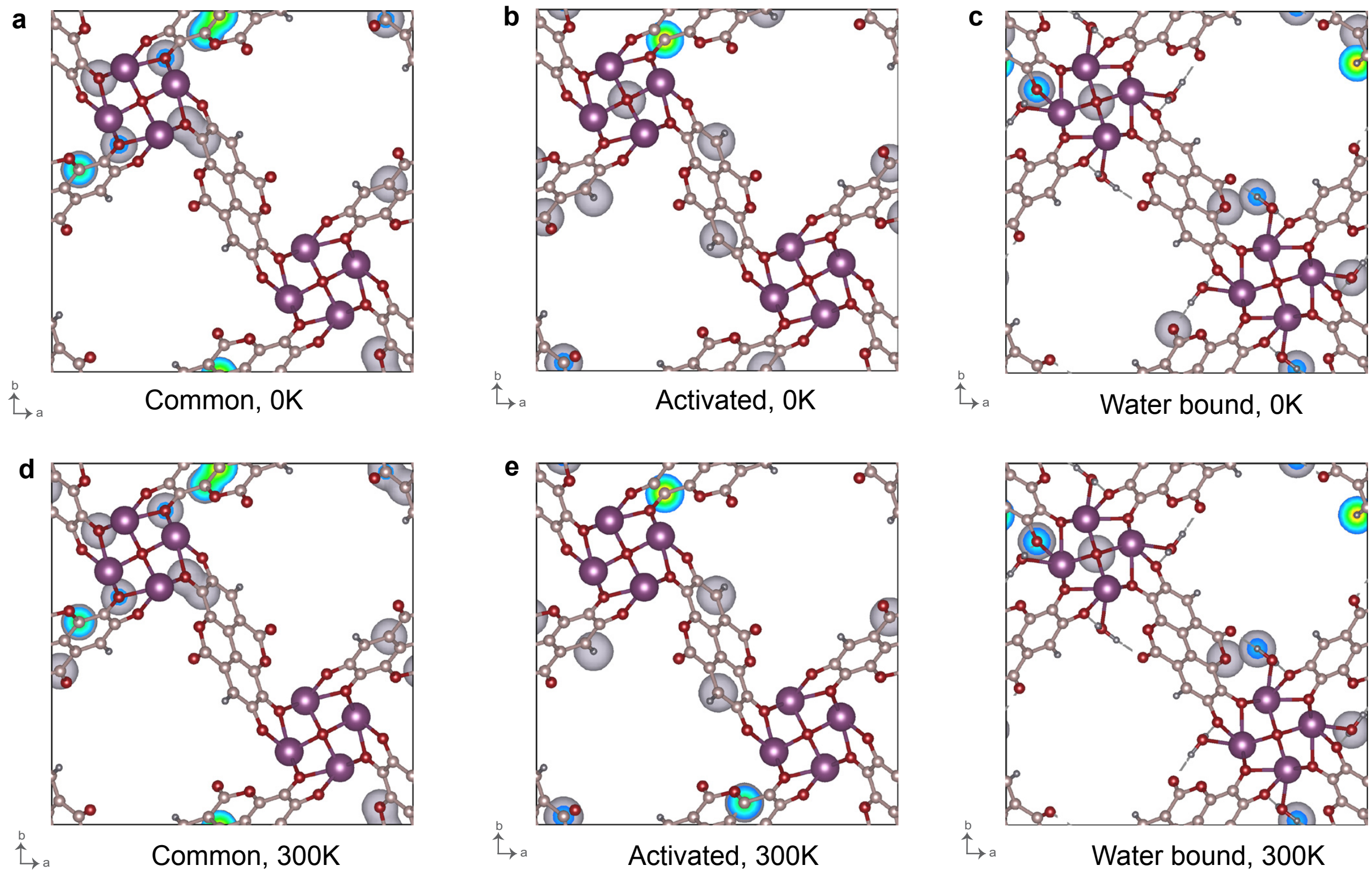

Figure S10: Analysis of the 20 specific atoms with maximum displacement for the 10 MC-sampled configurations (at each temperature and for each case) corresponding to the linear combination of phonon modes that most resemble the average phonon effect on the electronic band gap. At 0 K, a. shows the relevant atoms with highest displacement that are common among activated and water bound SU-101; b. shows the relevant atoms unique to the activated case; and c. shows the relevant atoms unique to the water bound case. The same analysis at 300 K is shown in d. (common atoms), e. (unique to activated case), and f. (unique to water bound case). Relevant atoms are marked with a surrounding gray shaded sphere.

6.4 Mode contribution

The contribution of each phonon mode to the renormalization of the PBE gap is computed following the harmonic quadratic formulation, as described in Methods Section 4.2. Let us first analyze the mode-resolved phonon effects of the activated SU-101. At 0 K, the largest contributions to the gap renormalization are associated with aromatic ring vibrations, such as asymmetrical C=C stretching modes in the range of 1447-1450 cm^{-1}, as shown in Figure 4a-b, Figure S11. This suggests that, at low temperatures, the electronic properties of activated SU-101 are predominantly renormalized by the zero-point vibrational coupling with the organic linker. Lower frequency modes, such as those at 86 cm^{-1} (Figure 4c) associated with a collective tilting of the ellagate linker and Bi-O stretching, become activated with temperature and are more important to the gap renormalization at 300 K. An animated mode visualization is available in the Supplementary Movies 1-6 for each mode in Figure 4.

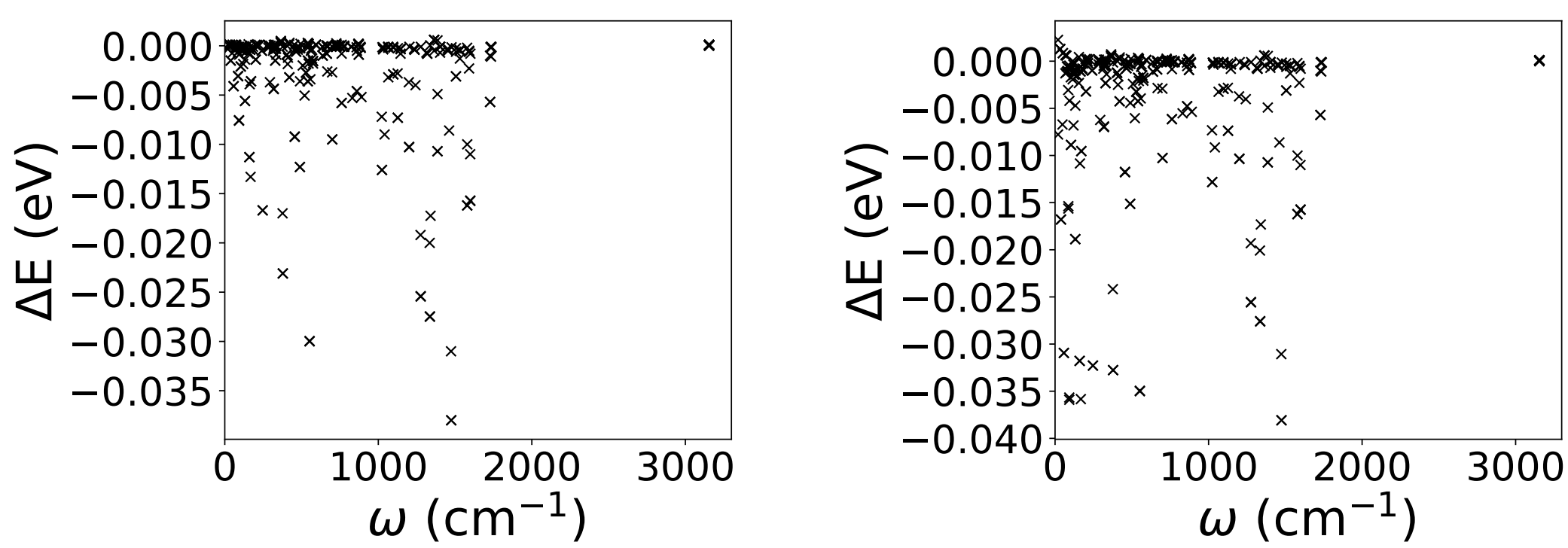

Figure S11: Mode-resolved PBE gap renormalization for activated SU-101 at a) 0K and b) 300 K.

Consistently, previous studies on a series of MOFs report that the high-frequency region of the phonon density of states is dominated by linker-based vibrations [23]. We expect these high-frequency modes associated with ellagic acid π-system to contribute to strong exciton-phonon coupling. Exciton-phonon coupling is a potential mechanism behind the recently reported drug release upon light absorption [13], resulting from the breaking of hydrogen bonds between the drug and the carbonyl oxygens. The coupling of excitons to

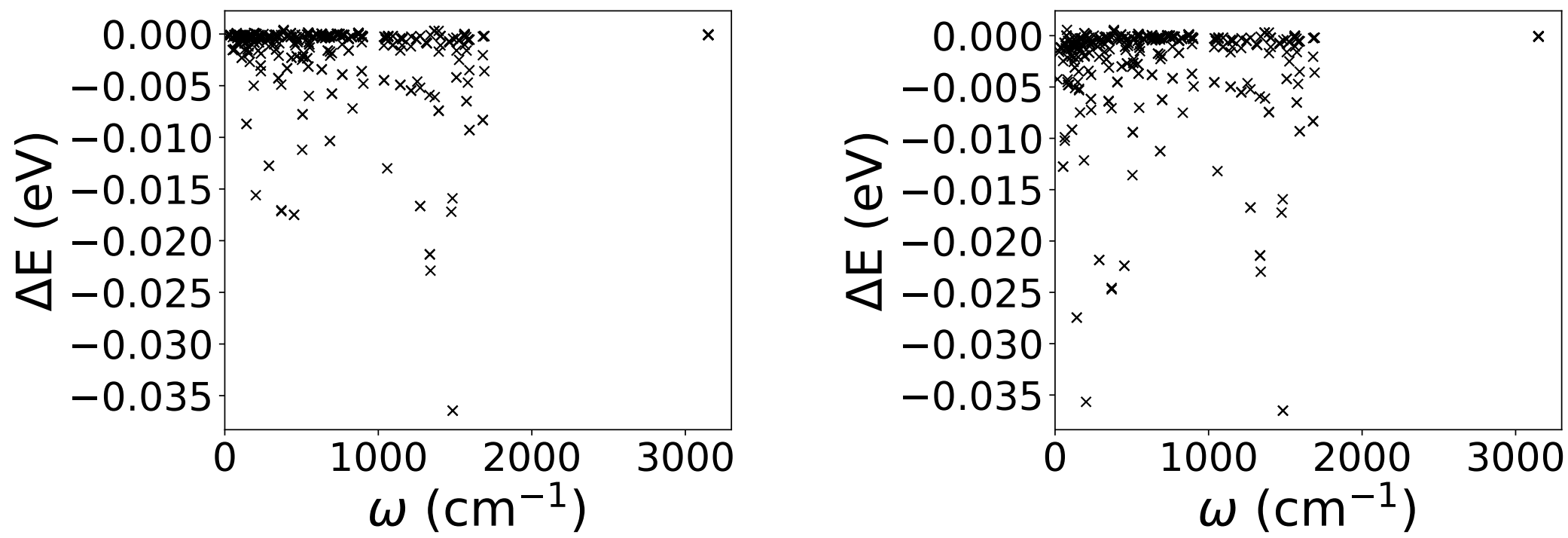

Figure S12: Mode-resolved PBE gap renormalization for water-bound SU-101 at a) 0K and b) 300 K.

high-frequency modes can be detrimental to the utilization of charge carriers for surface reactions, due to rapid non-radiative decay dynamics [24].

For the water-bound SU-101, the most relevant phonon modes at 0 K are similar to those of activated SU-101 and consist of asymmetric C=C stretching. The main differences between the two systems appear at 300 K. For water-bound SU-101, low-frequency modes associated with the rotation of the Bi-OH2 bond become important. For activated SU-101, these modes are not present, and instead the collective tilting of the ellagate linkers gains importance. This difference between important low-frequency modes at 300 K explains the mechanism behind the hydration-dependent electron-phonon coupling, and likely extend to the excitonic picture.

6.5 Phonon-driven renormalization of band gaps

The results show that the DFT-PBE band gaps of both activated and water-bound SU-101 have a large zero-point (0 K) renormalization of 280 ± 14 meV and 340 ± 17 meV, respectively. The gap is further renormalized at 300 K by 10 meV for activated and 94 meV for water-bound cases, suggesting that high-frequency phonons dominate the zero-point contribution. The large zero-point renormalization values for SU-101 is much greater than seen in typical inorganic semiconductors, which usually vary between a few meV to 100 meV [25]. The large zero-point contribution aligns with values found in other carbon-based materials like diamond and molecular crystals [25], and is expected, given that the organic components of the MOF are more likely to contribute to the high-frequency vibration modes associated with the organic linkers in SU-101.

Table S1: PBE gaps with and without the effect of phonons at 0 K and 300 K

	**Bound water**	**Activated**
$E_{KS,PBE}$ (no phonons) [eV]	1.91	1.62
$E_{KS,PBE}$ (0 K) [eV]	1.57 ± 0.02	1.34 ± 0.01
$E_{KS,PBE}$ (300 K) [eV]	1.48 ± 0.02	1.25 ± 0.02

Table S2: Renormalization of PBE, G_0W_0@PBE, and optical gaps, at 0 K and 300 K

	**Bound water**	**Activated**
$E_{KS,PBE}$ (0 K) [meV]	340 ± 17	280 ± 14
$E_{KS,PBE}$ (300 K) [meV]	434 ± 19	370 ± 16
$E_{G_0W_0@PBE}$ (0 K) [meV]	176 ± 34	310 ± 28
$E_{G_0W_0@PBE}$ (300 K) [meV]	269 ± 34	430 ± 25
E_{opt} (0 K) [meV]	270 ± 24	240 ± 30
E_{opt} (300 K) [meV]	410 ± 57	380 ± 41

7 Optical Properties Measurements

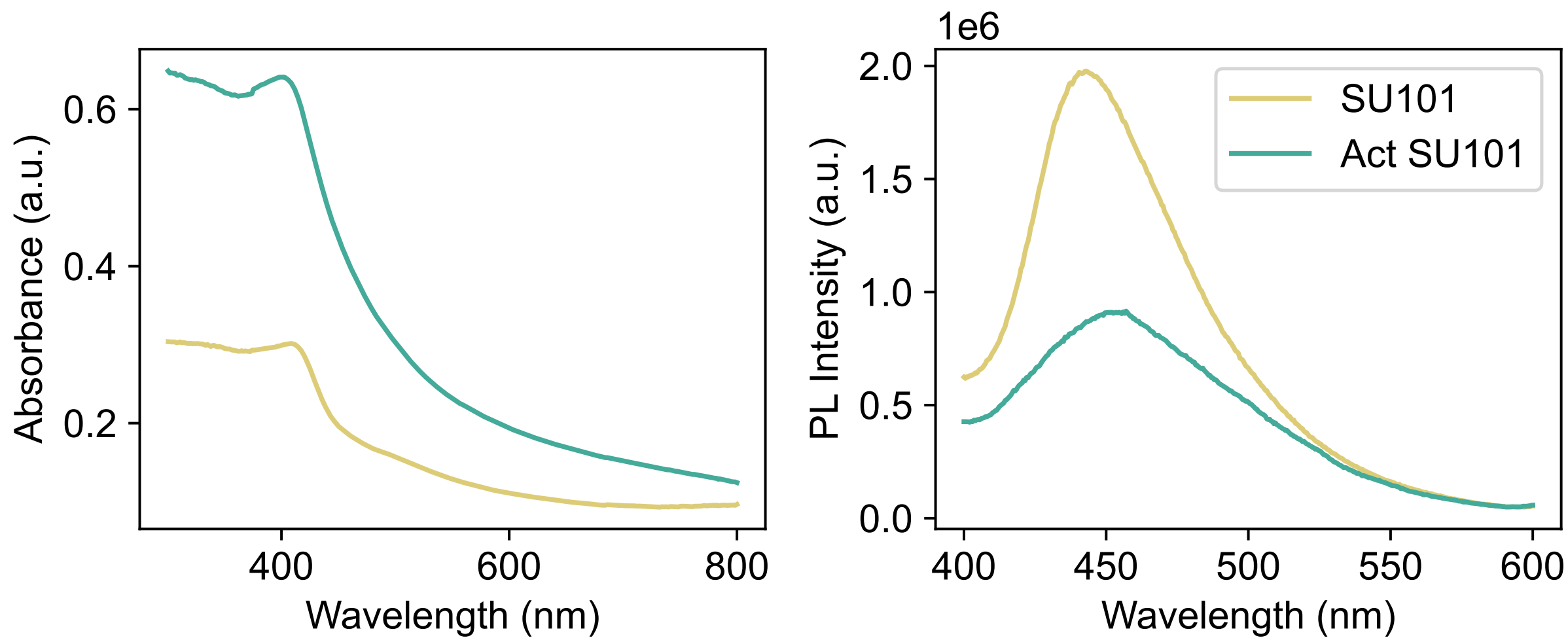

Figure S13: **Optical properties. a)** Ultraviolet-visible (UV-Vis) absorption spectra; and **b)** Photoluminescence (PL) emission spectra of the as-made and activated materials of SU-101.

Experimentally, we studied the optical properties of this material before and after activation. Both the as-made and activated SU-101 show similar ultraviolet-visible (UV-Vis) absorption spectra, with a sharp increase in absorbance between $\sim$ 2.25 - 2.75 eV. The activated material shows significantly higher absorbance compared to the as-synthesized material (Figure S13 a). The open-metal sites (OMS) created upon activation, due to the removal of coordinated H_2O molecules from the Bi metal center, seem to enhance the absorbance of the material. As described in the original publication, the ellagate ligands stack along the c-axis, showing π-π stacking interactions [1]. Activation leads to a contraction of lattice cell parameters a and b, accompanied by an expansion along the c-axis. This structural change, together with the changes in the H bonding network, may improve the π-π stacking interactions. This, in combination with the creation of OMS, could explain the improved absorbance.

We have experimentally probed the effect of activation (dehydration) on the optical gap of SU-101 using UV-vis diffuse reflectance spectroscopy and Tauc analysis (Figure S14). Both our G_0W_0-BSE and experimental results show a consistent decrease in the apparent

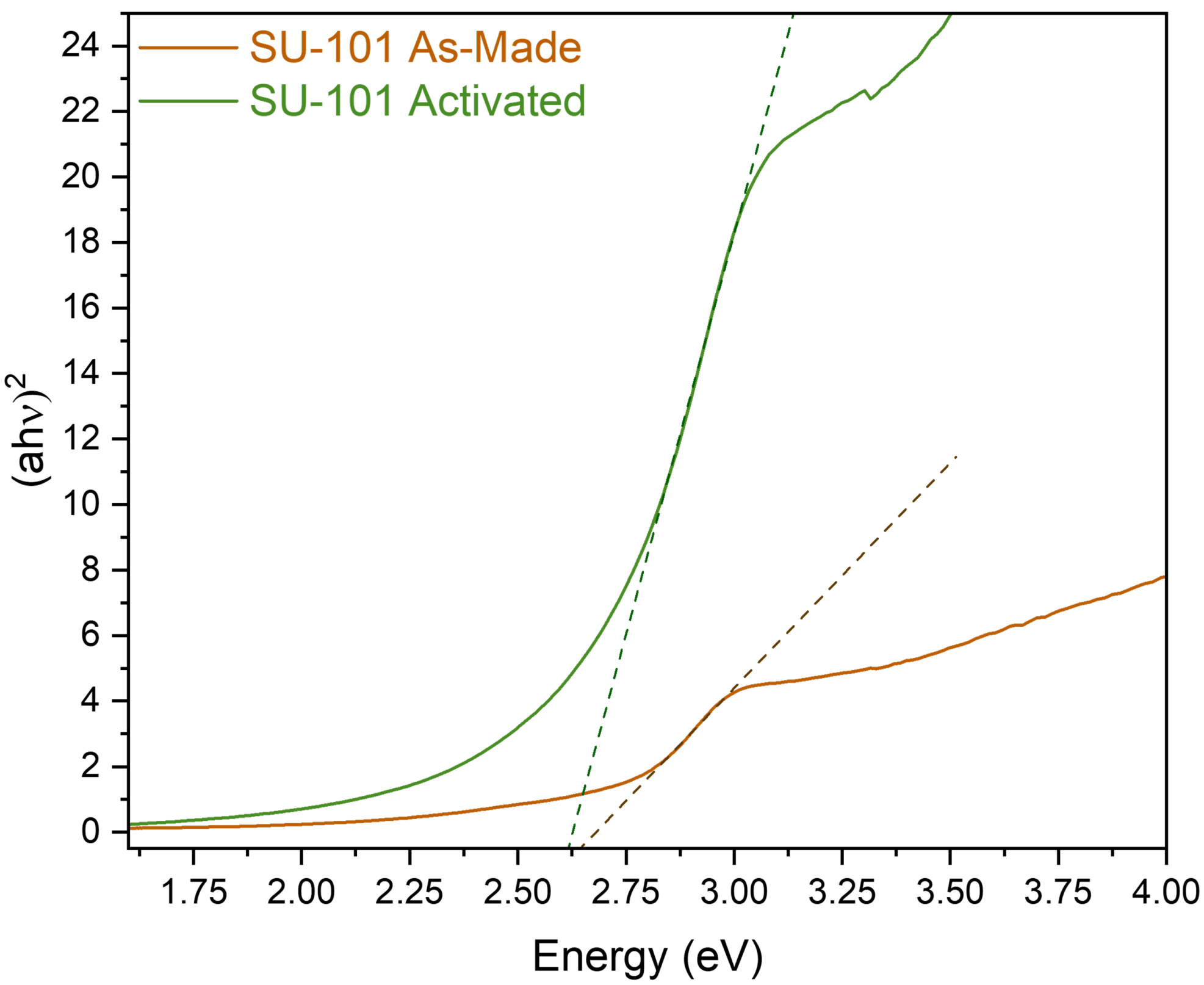

Figure S14: **Calculated Tauc plot** considering a direct transition. The calculated band gaps are 2.68 eV (as-made) and 2.63 eV (activated at 150°C for 12h after solvent exchange).

optical gap upon activation compared to the as-made material. This trend is reproducible across independently prepared samples and is in qualitative agreement with the theoretical prediction that dehydration reduces the gap.

The photoluminescence (PL) emission spectra show an inverse trend, with weaker emission from the activated material relative to its as-synthesized counterpart (Figure S13 b). Additionally, the emission of the activated material exhibits broadening and a redshift. These features may be ascribed to disorder at the Bi sites, with some residual H_2O molecules possibly remaining at certain sites, as well as a distribution of overlapping electronic states. The slight red shift in the emission of the activated structure with respect to the as-synthesized form is consistent with the reduced optical band gap calculated from the Tauc plot considering

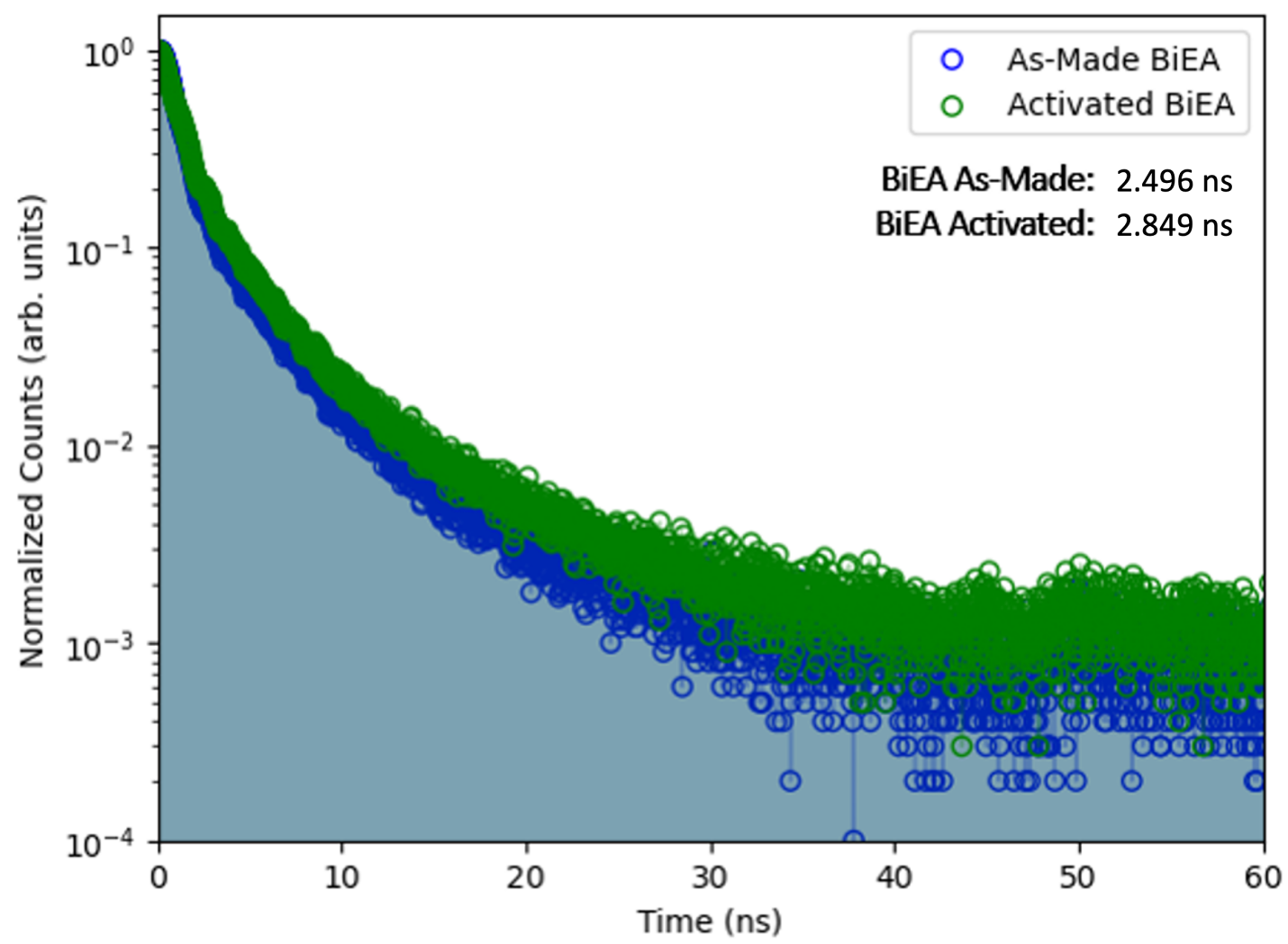

Figure S15: Transient decay of photoluminescence from as-synthesized (blue) and activated (green) SU-101.

a direct transition (Figure S14). These results are also in agreement with the results from G_0W_0-BSE calculations. Similar bathochromic shifts have also been found in other MOFs due to improved π-π intermolecular interaction between the ligands [26].

Additionally, we have performed time-resolved photoluminescence measurements. The average PL lifetime increases from 2.496 ns in the water-bound material to 2.849 ns after activation, as shown in Figure S15.

We performed additional UV-vis and photoluminescence measurements in the solid state for SU-101, including (i) the as-synthesised material, (ii) samples activated at 150 °C, and (iii) samples activated at 200 °C (Figure S16,Figure S17). These activated samples were prepared without prior solvent exchange. We observe that the photoluminescence spectra in the solid state exhibit more structured features compared to the smoother emission profiles obtained in suspension. The difference is consistent with increased spectral broadening in suspension, where dynamic disorder and solvent interactions lead to the merging of individual transitions to form a continuous band. Importantly, the overall trends upon activation remain consistent between the two measurement approaches, supporting that the observed changes in optical

properties are intrinsic to the material and not driven by solvent effects.

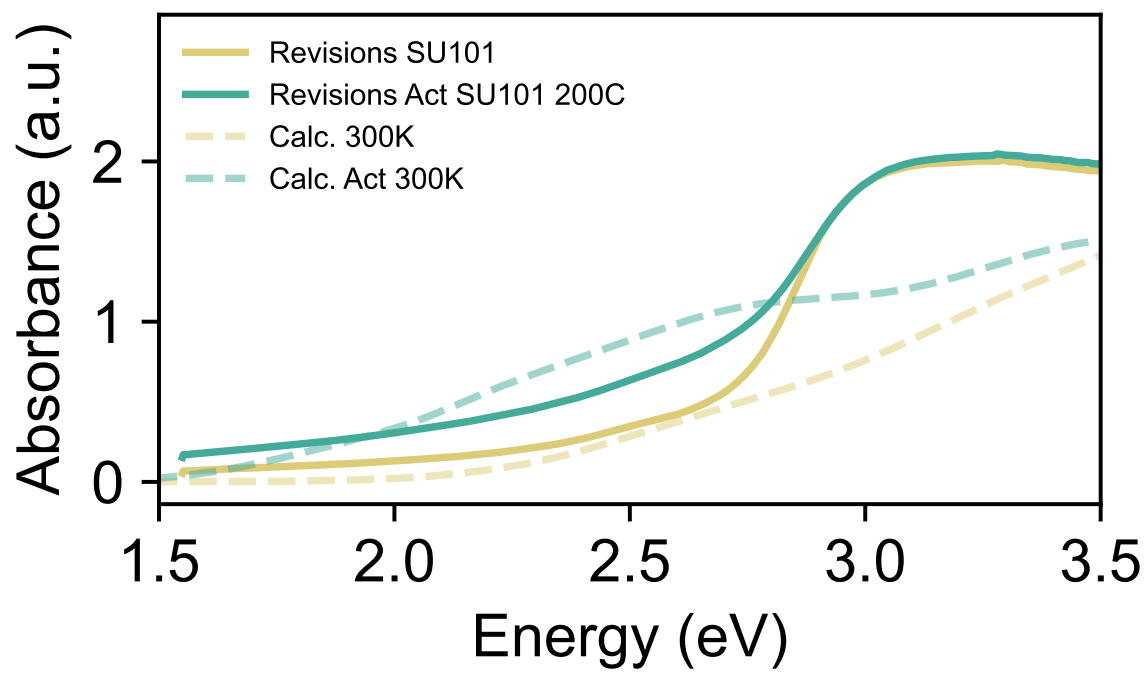

Figure S16: Solid-state ultraviolet-visible (UV-Vis) absorption spectra for activated and water-bound SU-101 and comparison with theoretical results including phonons at 300 K.

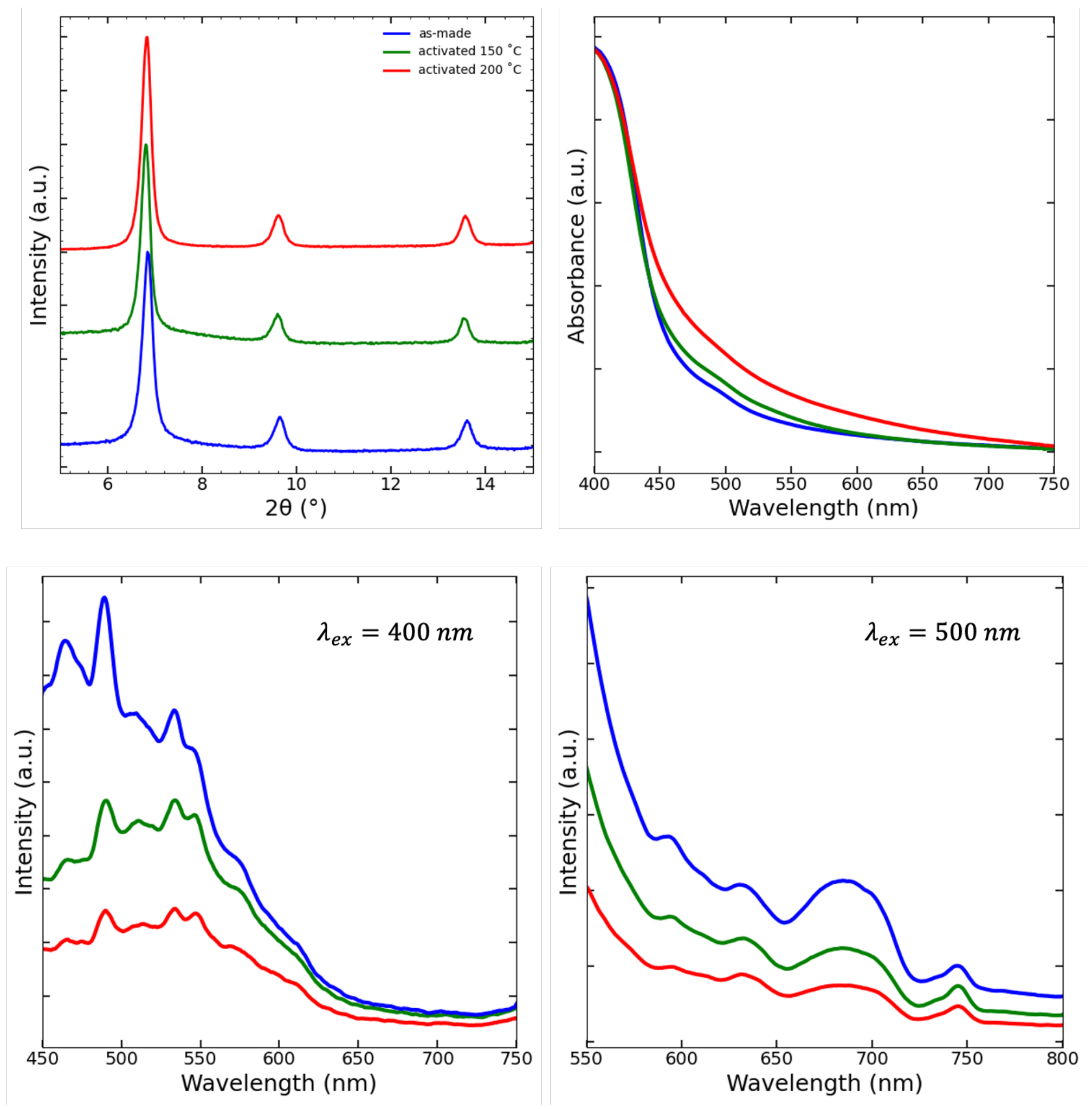

Figure S17: PXRD (top left), UV-Vis absorption (top right), and photoluminescence (bottom panel) data collected from SU-101 samples in the solid state. Blue traces represent as-synthesized SU-101, and green and red traces represent samples activated at 150 °C and 200 °C, respectively.

8 Absorption spectra with phonons

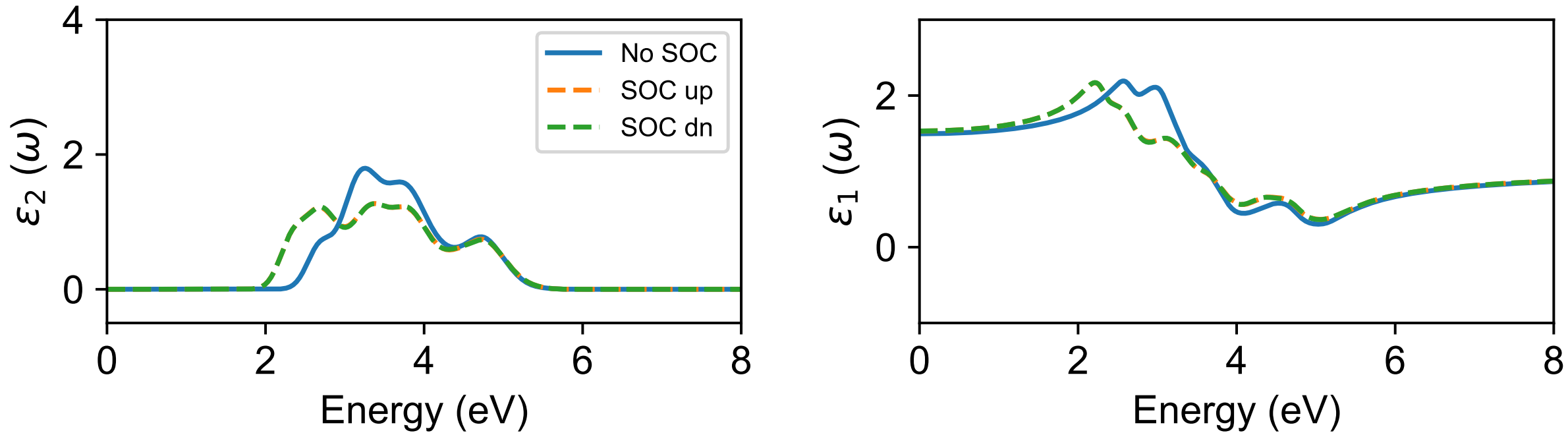

Figure S18: Computed G_0W_0-BSE absorption spectra for the activated SU-101 at 0 K for a representative configuration.

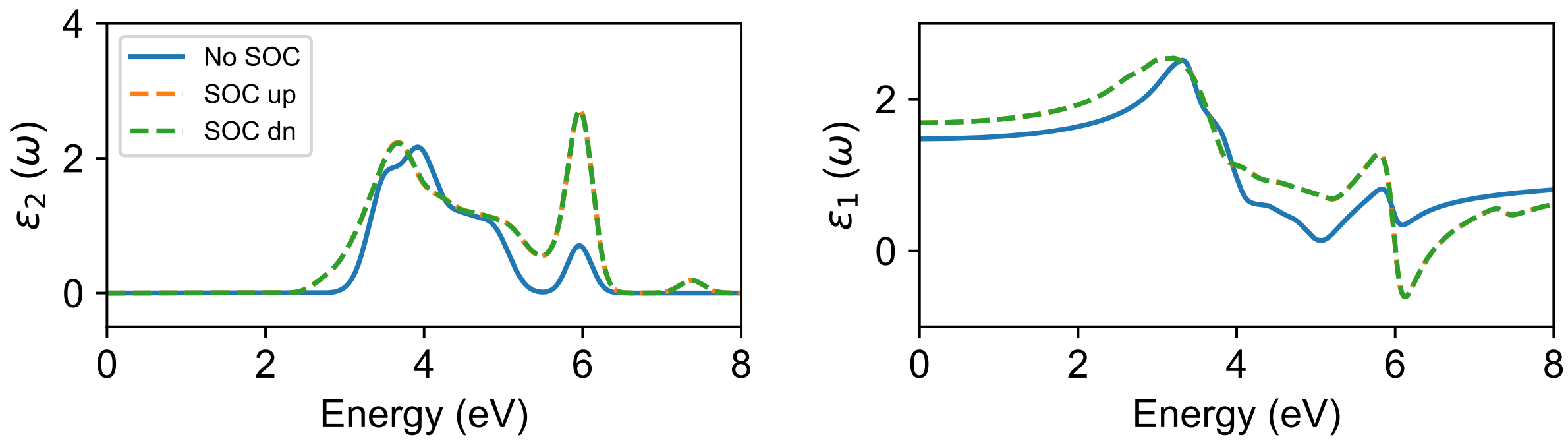

Figure S19: Computed G_0W_0-BSE absorption spectra for SU-101 at 0 K for a representative configuration with coordinated water (as-made).

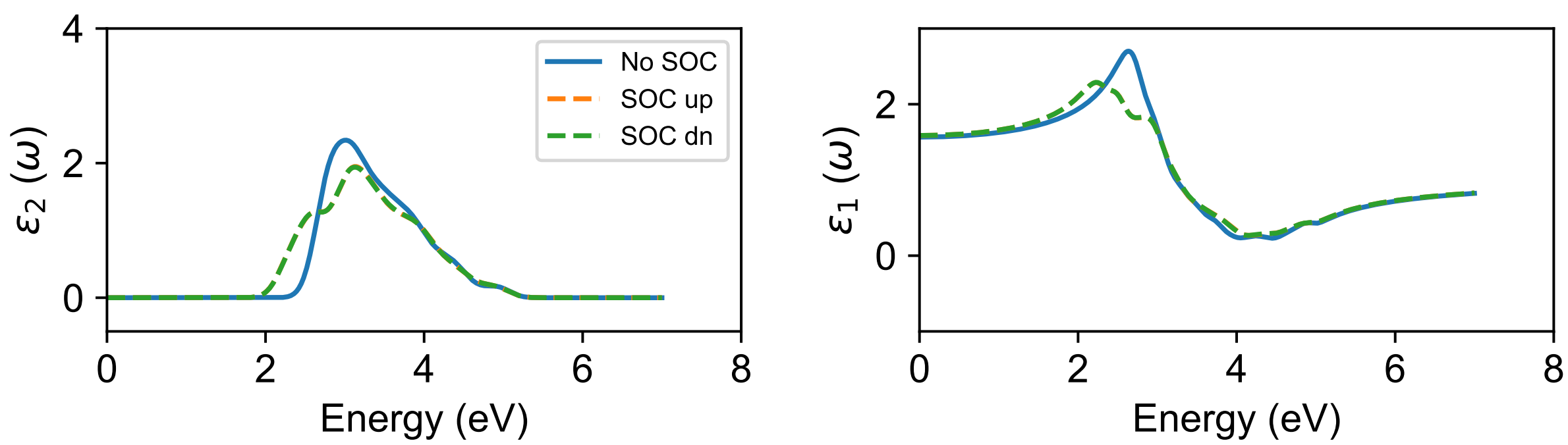

Figure S20: Computed G_0W_0-BSE absorption spectra for the activated SU-101 at 300 K for a representative configuration.

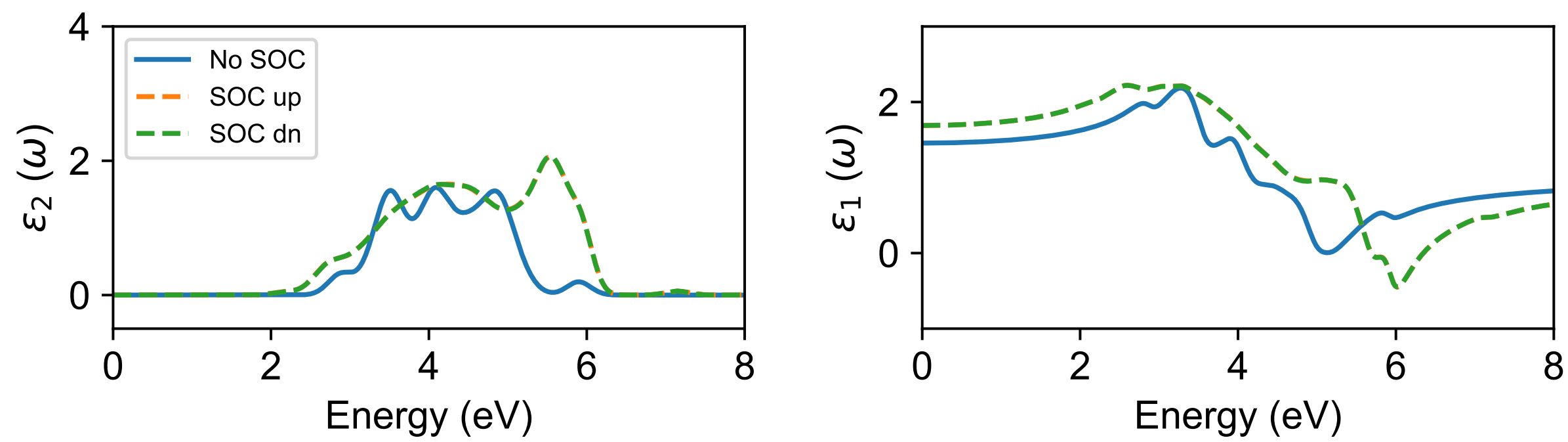

Figure S21: Computed G_0W_0-BSE absorption spectra for SU-101 at 300 K for a representative configuration with coordinated water (as-made).

9 Exciton coefficients

Exciton coefficients in k-space are obtained by solving the BSE within the Tamm-Dancoff approximation, as implemented in BerkeleyGW [27–29].

$$|S\rangle = \sum_{vck} A^S_{vck} |vck\rangle \tag{S1}$$

Equation (S1) describes the expression of each excitonic state $|S\rangle$ as a linear combination of electron-hole transitions $|vck\rangle$, where A^S_{vck} are the exciton expansion coefficients that describe the electron-hole pair amplitude of the exciton wavefunction in reciprocal space [30, 31].

By examining A^S_{vck}, it is possible to analyze the exciton character in reciprocal space and how the Bloch states $|n\mathbf{k}\rangle$ contribute to a given exciton [31].

Figure S22 shows the exciton amplitude squared, $\Sigma|A(k)|^2$, in k-space for the first exciton of different configurations.

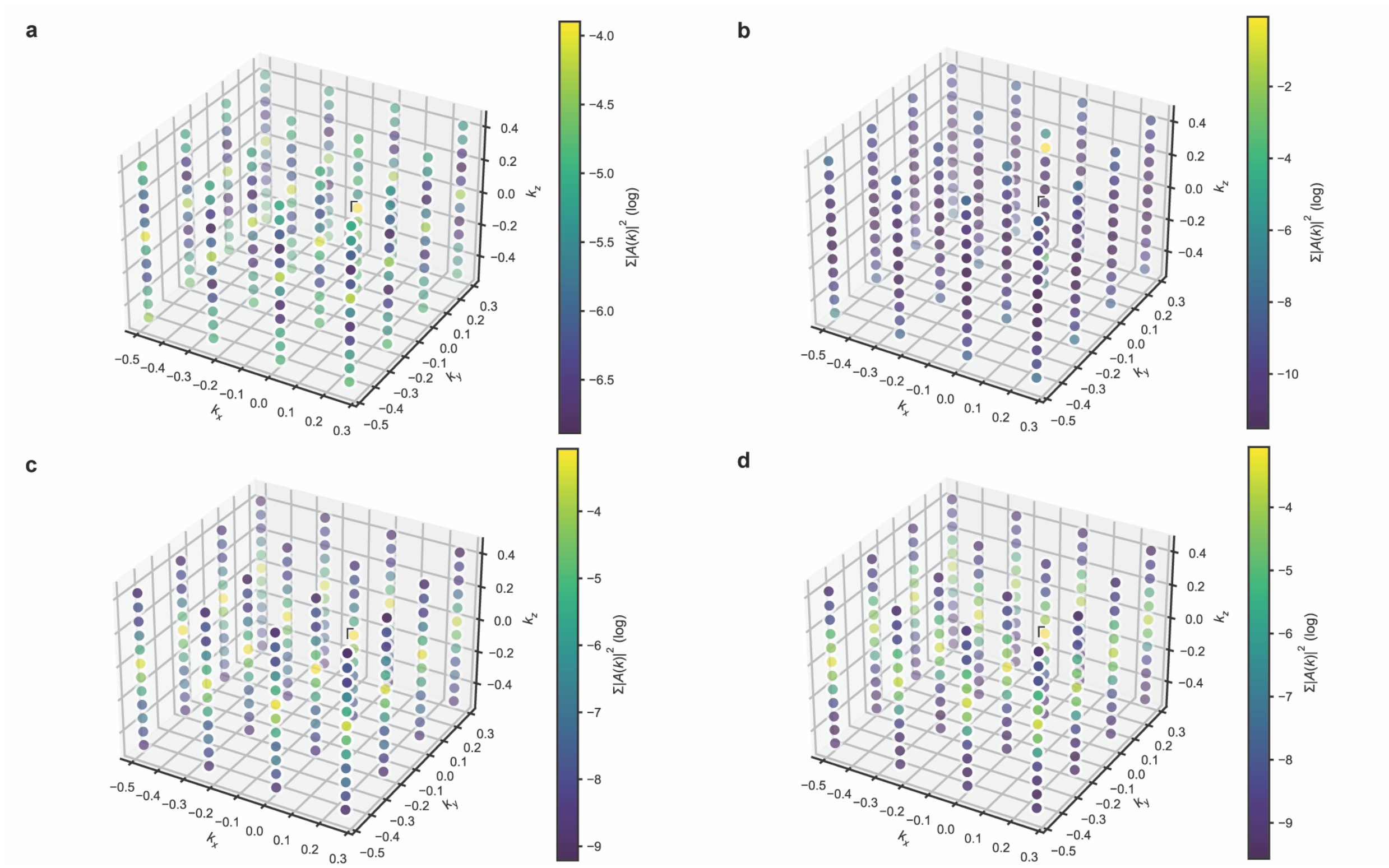

Figure S22: First exciton amplitude squared in k-space for a. static configuration (bare), b. water coordinated, c. one displaced configuration for activated SU-101 at 0 K, d. one displaced configuration for activated SU-101 at 300 K.

10 Spin-orbit coupling

Given the presence of a heavy metal in SU-101, we first investigated whether the inclusion of spin-orbit coupling (SOC) affects its electronic properties, especially regarding the valence and conduction bands. As shown in Figure S23, in the case of the activated MOF, the inclusion of SOC leaves the valence band mostly unchanged but lowers the energy of the Bi unoccupied states. Therefore, the character of the conduction band is changed from predominantly linker-based to metal-based. This change in character results primarily from a systematic shift of the unoccupied Bi states and a reordering of the bands. Consequently, the GGA Kohn-Sham electronic gap is reduced by 660 meV, and the nature of the band gap changes from indirect to direct at the Γ-point with the inclusion of SOC. Moreover, SOC alters the structural relaxation, leading to a different local minimum structure, as shown in Figure S24 and discussed in Section 10.1. Given the significant quantitative and qualitative differences in the electronic properties introduced by SOC, we included it to evaluate the effects of solvation, phonons, and excitons.

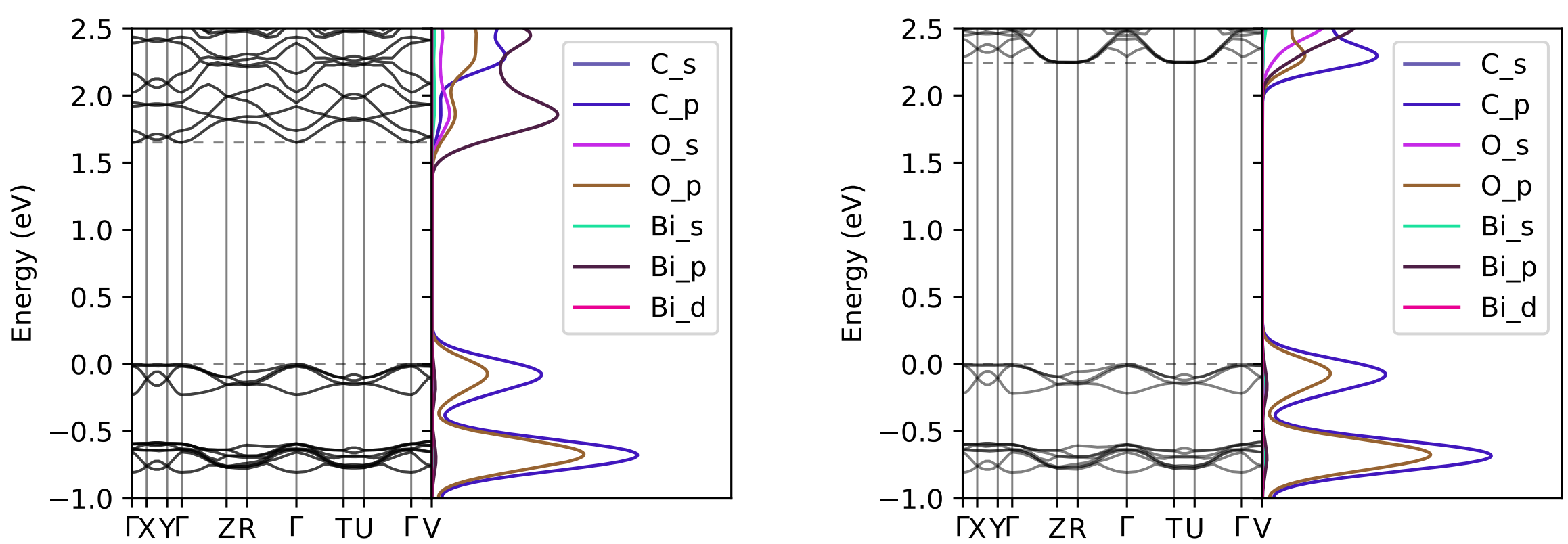

Figure S23: Band structure of static configuration (bare) a) with and b) without SOC.

10.1 Potential energy surface

We noticed a shift between the potential energy surface (PES) of the ideal harmonic approximation (through fitting) and the computed PES (with SOC) for a given phonon mode for the

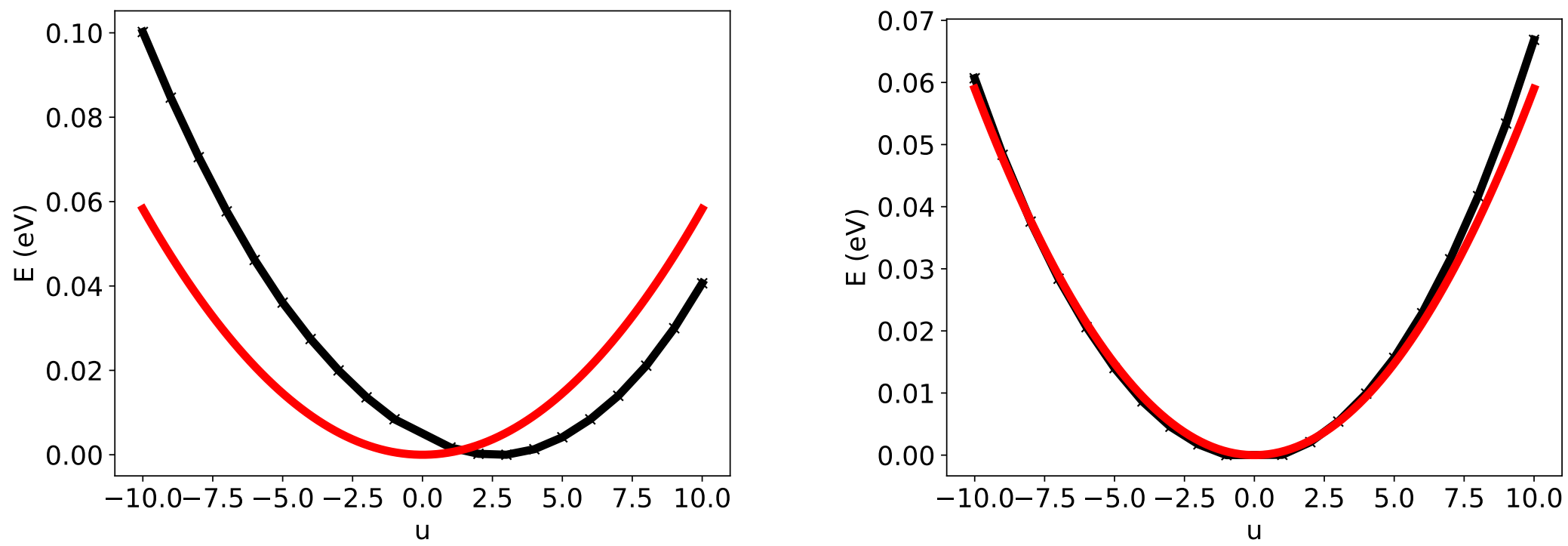

Figure S24: Comparing harmonic (red) and anharmonic (black) potential energy surfaces of SU-101 structure relaxed a) without SOC and b) with SOC, for a low-frequency phonon mode of $\approx 19\,\mathrm{cm}^{-1}$. PES computed with the inclusion of SOC for both cases. The harmonic PES is fitted assuming perfect harmonic behavior given the phonon frequency. The phonon displacement u is given in units of the zero-point width $\frac{1}{\sqrt{2\omega}}$

structure relaxed without the inclusion of SOC, as shown in Figure S24a. The observed shift comprises an instability that indicates that, along this chosen mode, there is a lower energy configuration (around 2.5 units of u). When relaxing the structure with SOC, the instability is resolved, and the harmonic and anharmonic PES agree, as displayed in Figure S24b. Therefore, we can safely consider the harmonic approximation for the proceeding calculations including SOC, as long as the structure of use is the one relaxed with the inclusion of SOC.

10.2 Band structures

At both 0 K and 300 K, the band structures suggest a symmetry breaking that occurs due to electron-phonon coupling, as displayed in Figures S25 and S26. The involvement of Bi orbitals in the conduction band (CB) and the strong SOC suggest that this symmetry breaking can lead to Rashba splitting in the CB, as observed for the example of a displaced configuration at 300 K in Figure S26c. The band splitting is more pronounced along the direction of the channels formed by the metal node (c direction, and $\Gamma \rightarrow Z$ in reciprocal space), which can be due to higher Bi orbital contribution along this direction. While symmetry breaking and Rashba effects intuitively disappear at thermal equilibrium, it has been discussed in

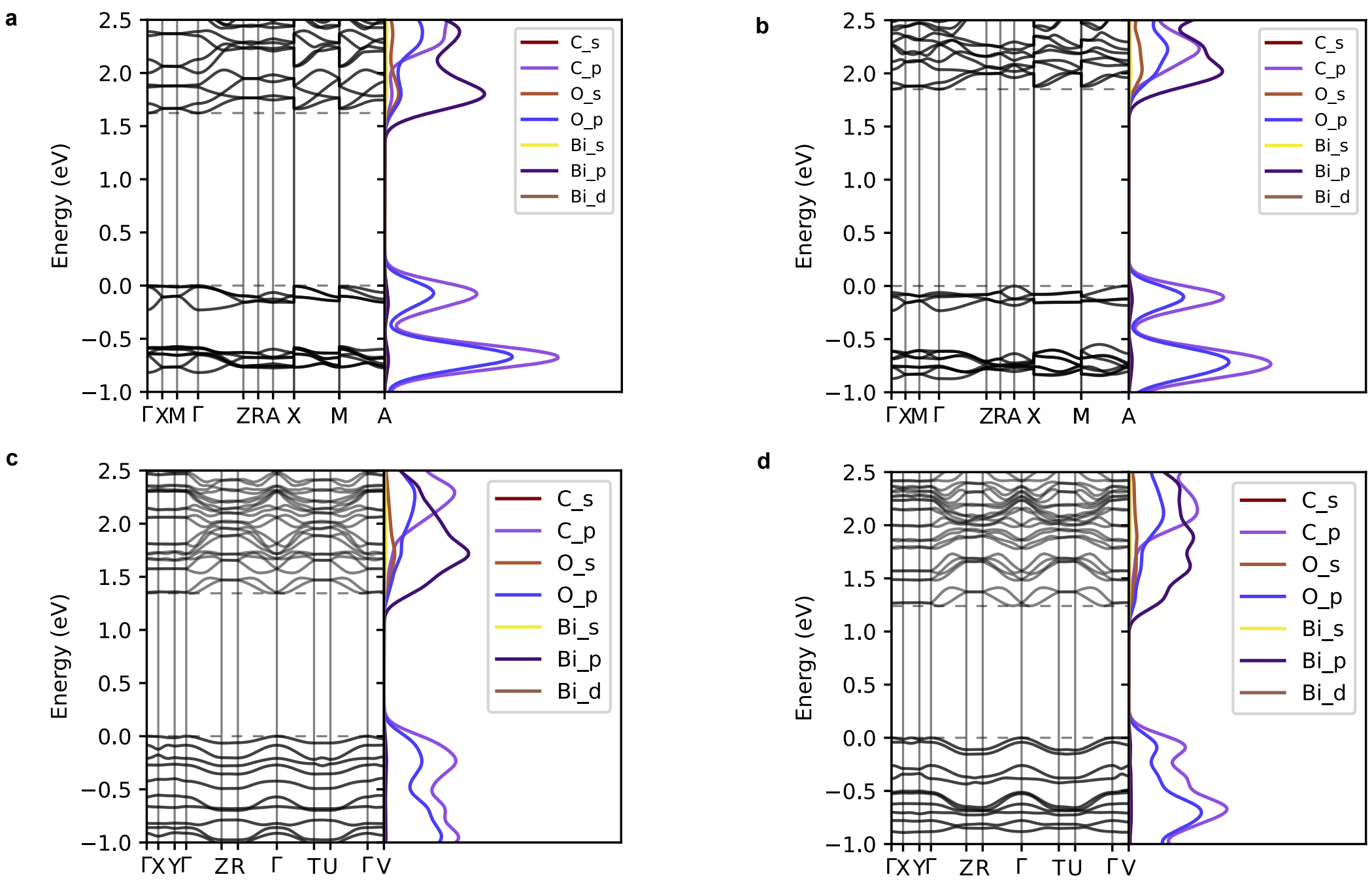

Figure S25: PBE band structure (with SOC) for a) bare, b) water, c) example of displaced configuration at 0 K and d) example of displaced configuration at 300 K.

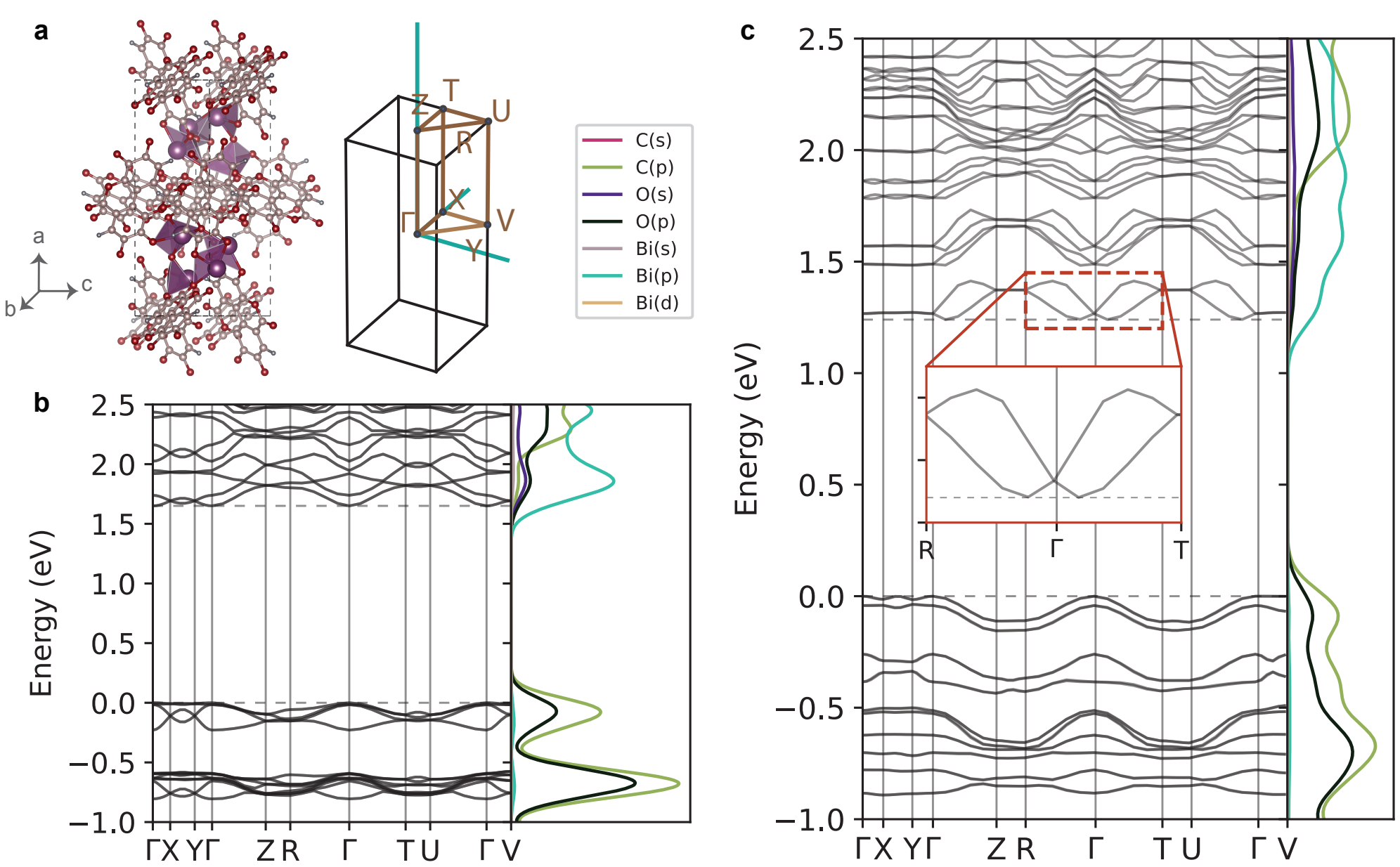

Figure S26: a. Static (undisplaced) SU-101_0 and corresponding Brillouin zone. b. Band structure of SU-101_0 and of c. SU-$101_{300\,K}$, the latter displaying Rashba splitting in the conduction band (see zoomed area).

the literature that out-of-equilibrium phonons can cause time-dependent inversion symmetry breaking, and the observation of dynamic Rashba effects [32–34].

11 GW-BSE convergence tests

11.1 GW

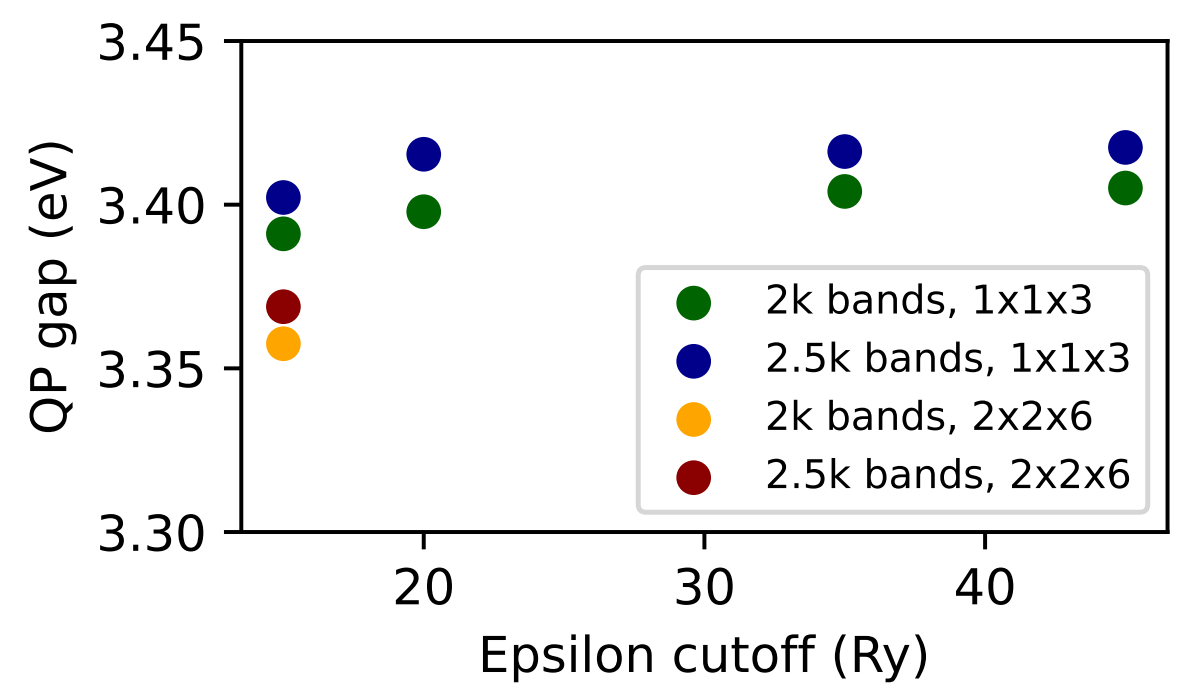

Figure S27: Convergence of quasiparticle band gap with respect to the epsilon cutoff and number of bands using G_0W_0@PBE.

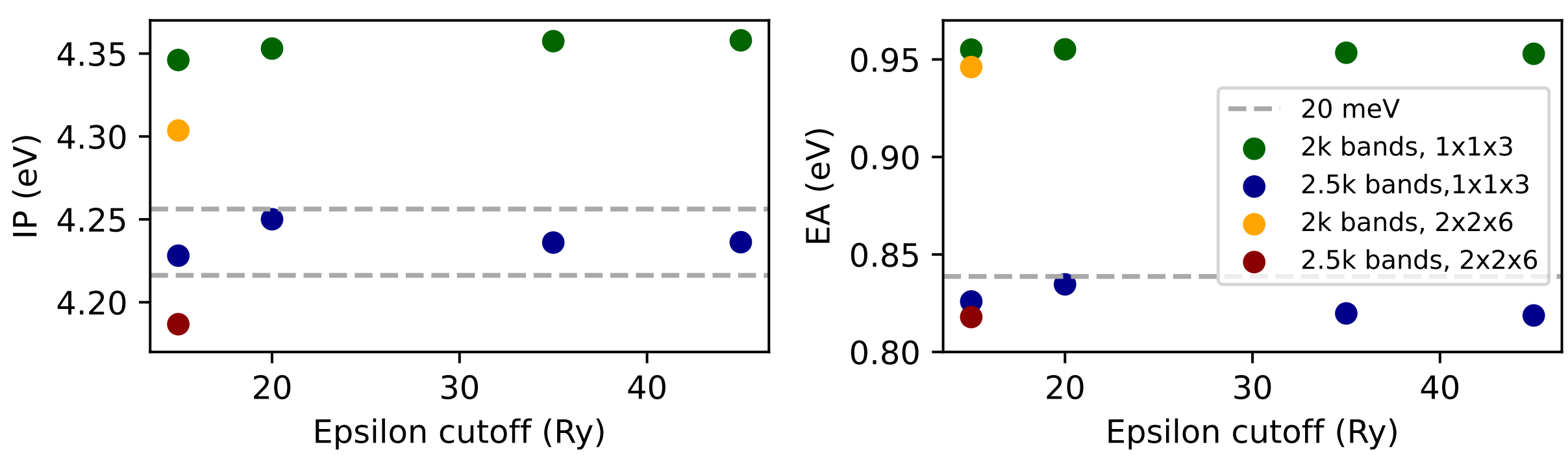

Figure S28: Convergence of GW ionization potential and electron affinity with respect to the epsilon cutoff and number of bands using G_0W_0@PBE.

11.2 Perturbative approach for including SOC

Figure S29 shows the overlap between bands with and without SOC to evaluate the re-ordering of bands. A one-to-one correspondence without re-ordering of bands would result in a diagonal line with high overlap (yellow diagonal dots). We observe that, although re-ordering seems to take place, the mixing of electronic states is low and therefore justifies our approximation. Moreover, the re-ordering of the relevant frontier states seems to take place only in the range

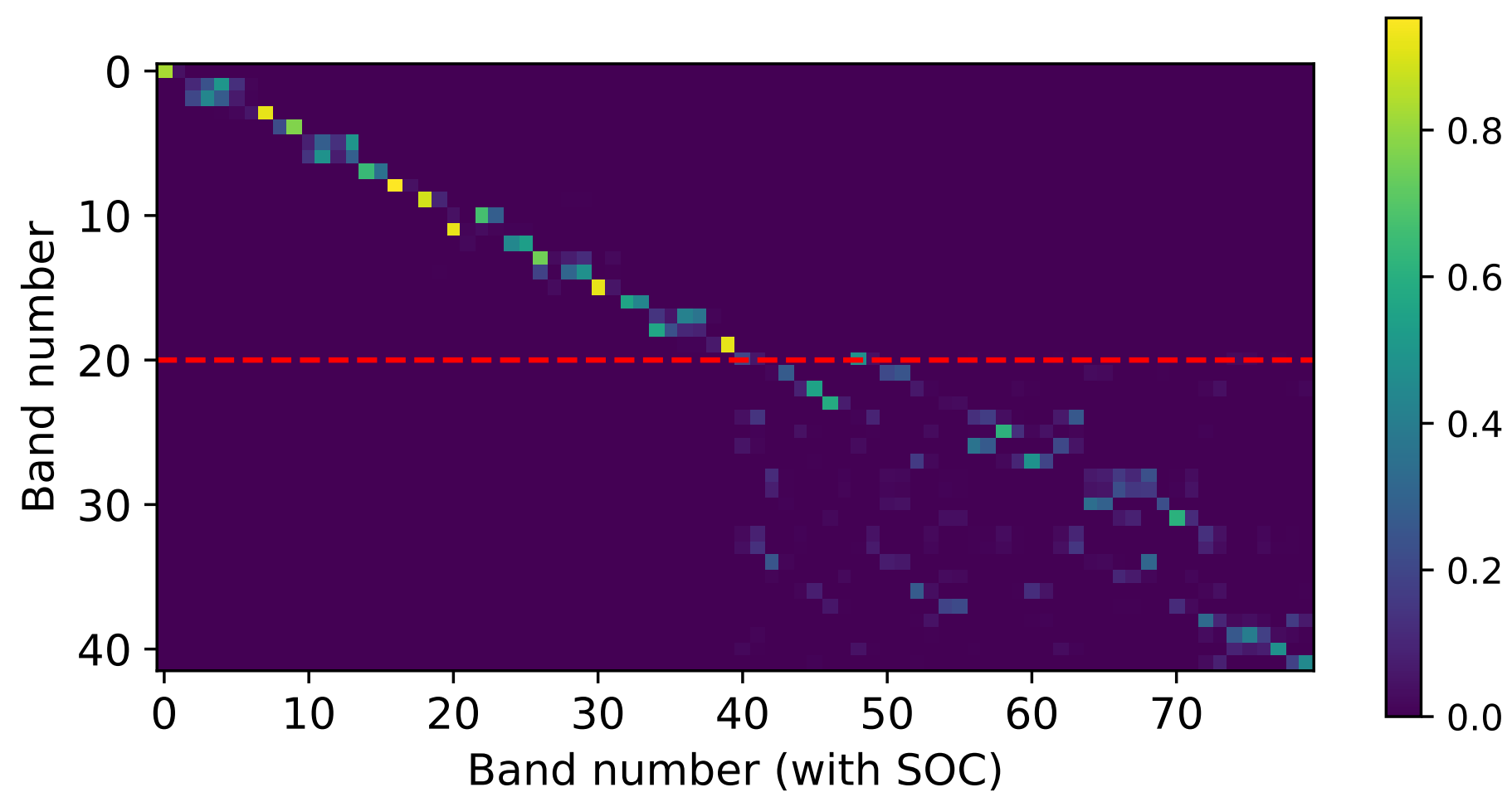

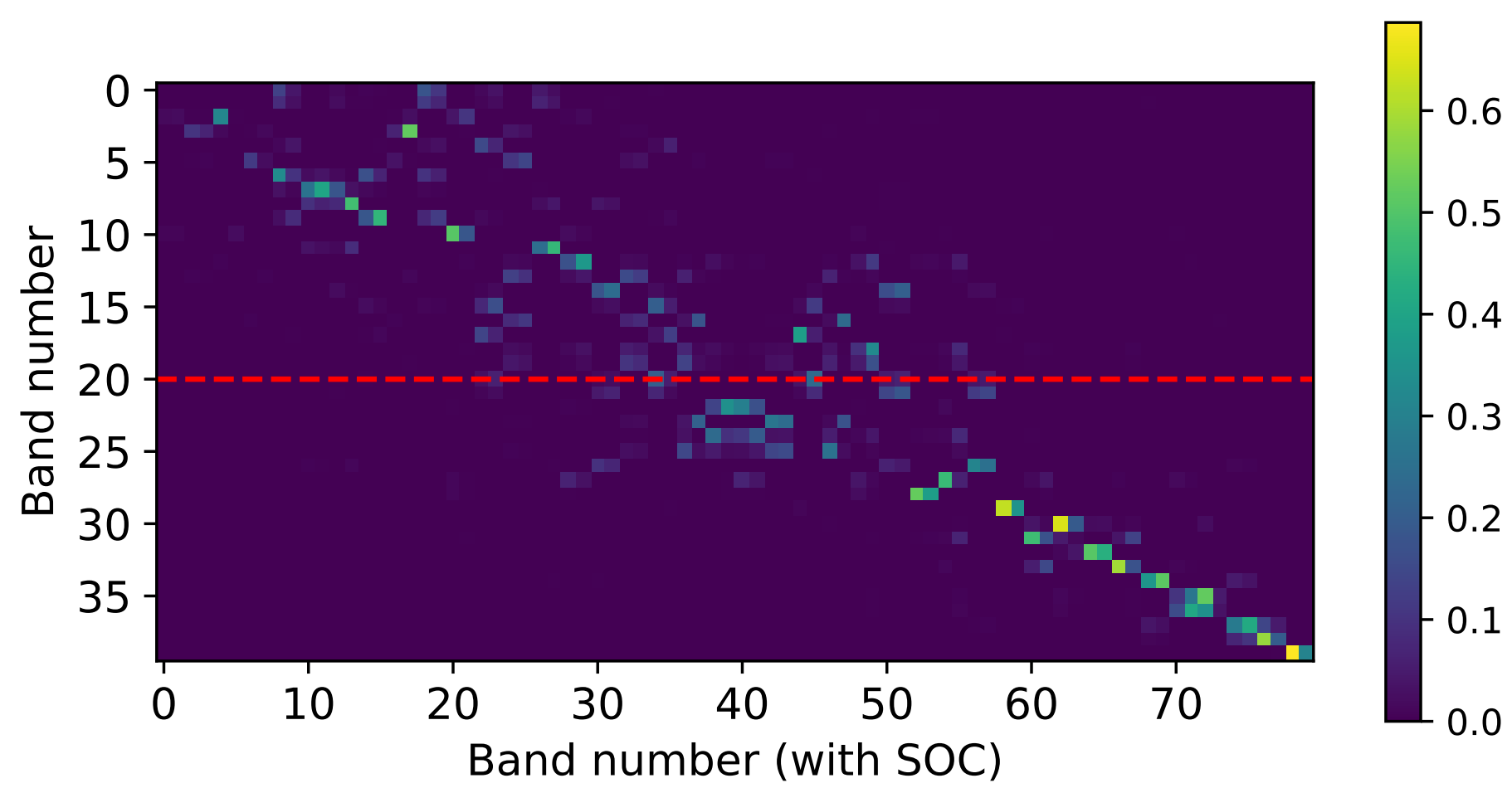

Figure S29: Overlap between the eigenvalues of bands with (x-axis) and without (x-axis) the inclusion of spin-orbit coupling, for both activated (upper plot) and water-bound (lower plot) SU-101 without the effect of phonons. The red dashed line indicates the valence band.

of 20 valence and 20 conduction bands (40 each with inclusion of SOC). For a more accurate treatment of spin-orbit coupling, its explicit treatment would be necessary [35]. However, given the high associated cost, we treat SOC perturbatively in all G_0W_0-BSE calculations.

11.3 BSE

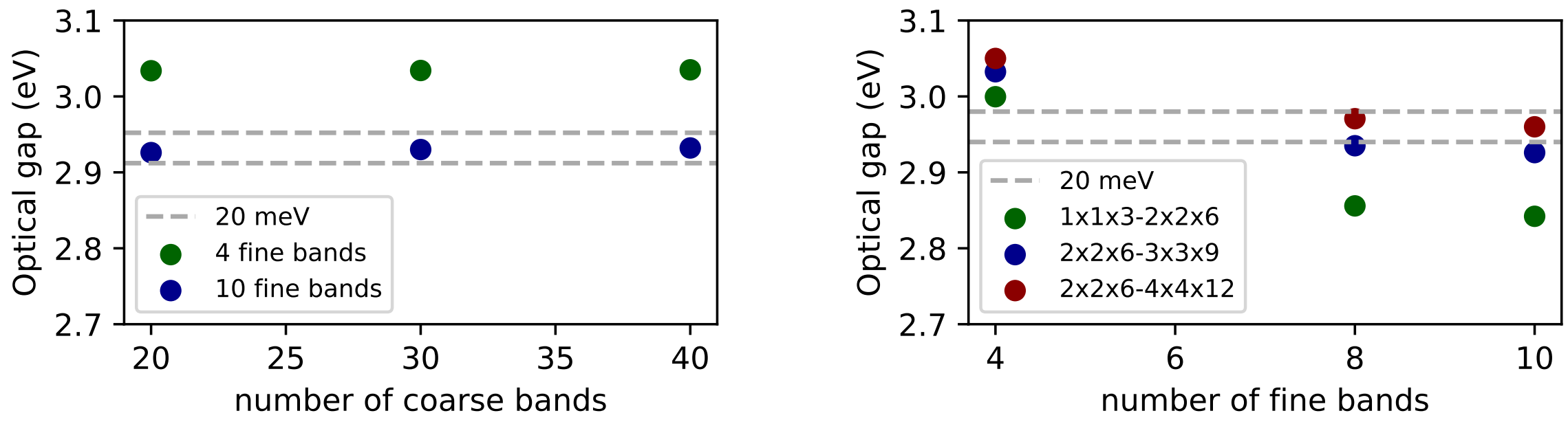

Figure S30: Convergence of G_0W_0-BSE optical gap with respect to the number of coarse and fine bands.

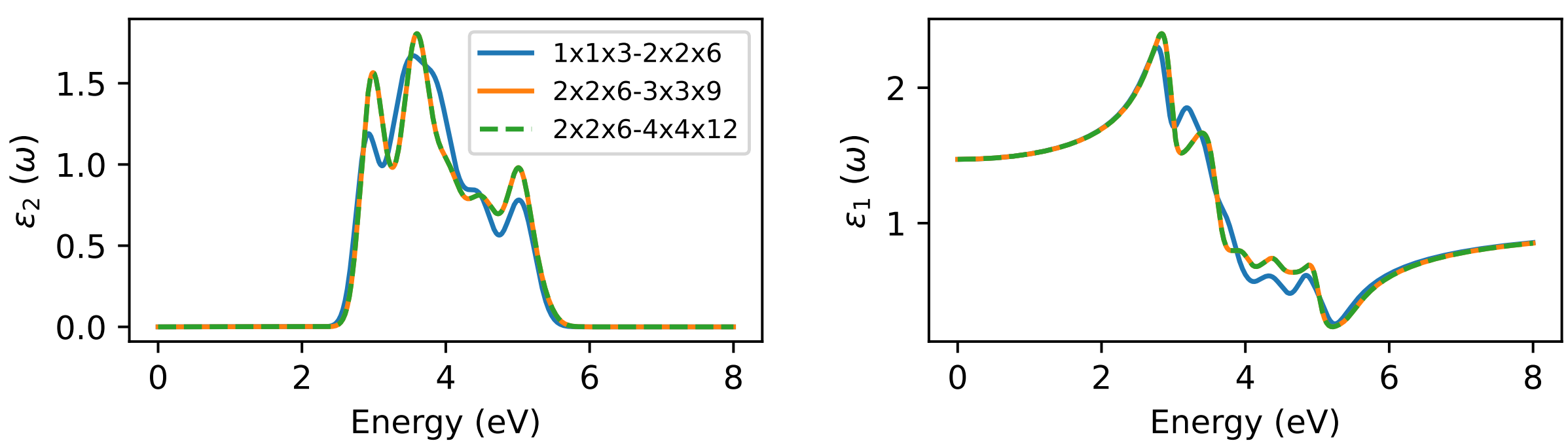

Figure S31: Convergence of G_0W_0-BSE absorption spectra with respect to different grid interpolations.

11.4 Displaced configurations

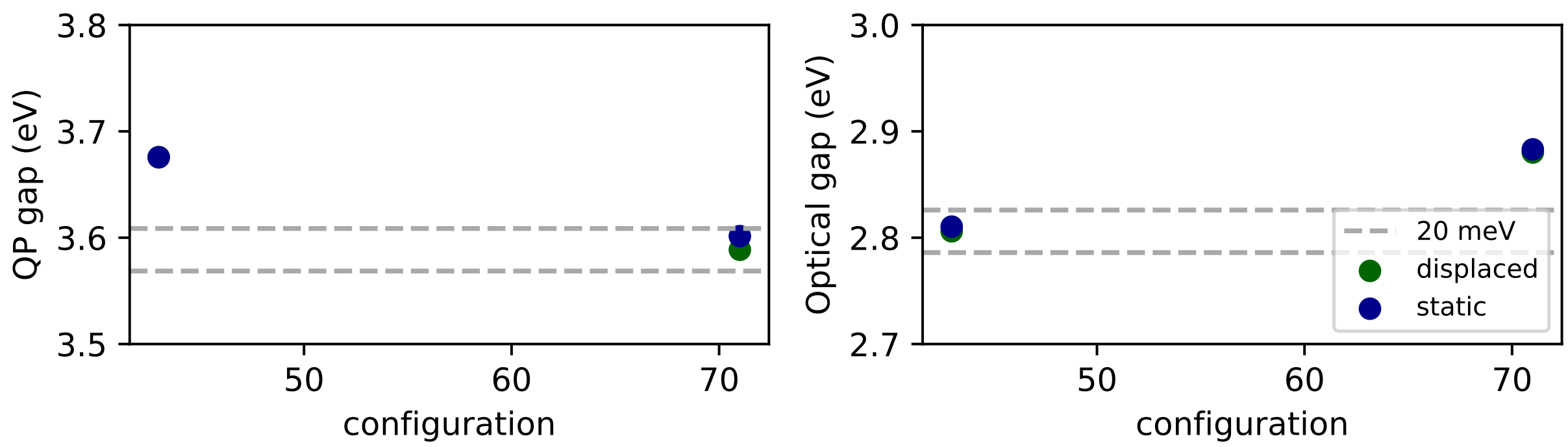

Figure S32: Test for using the dielectric function obtained from the static (undisplaced) calculation to compute G_0W_0-BSE quasiparticle and optical gaps of displaced configurations.

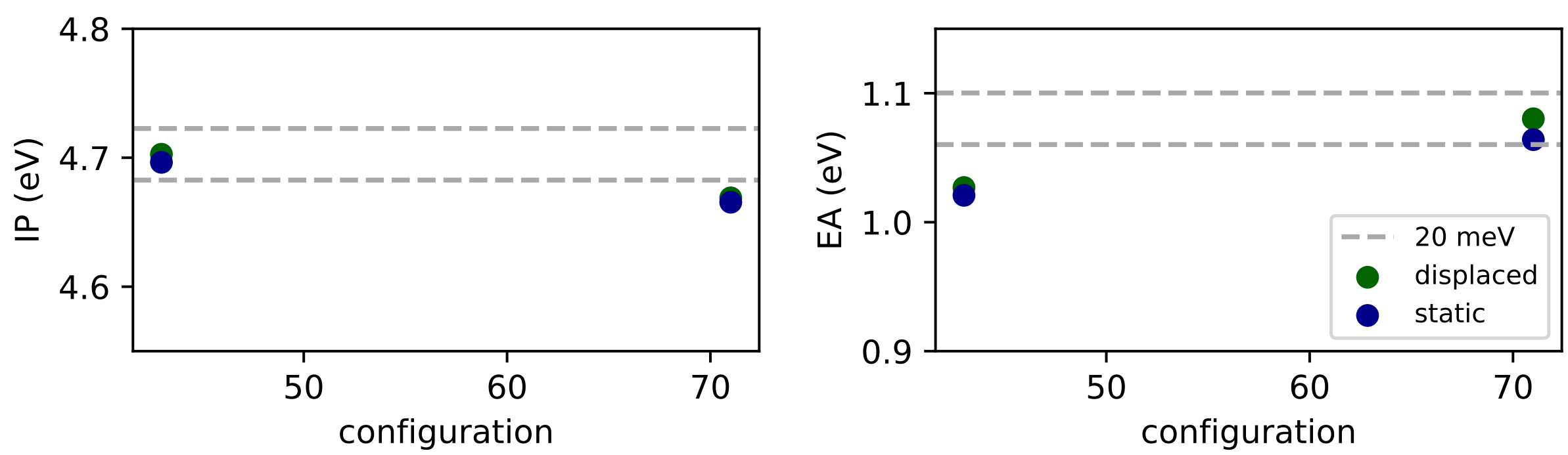

Figure S33: Test for using the dielectric function obtained from the static (undisplaced) calculation to compute GW IP and EA of displaced configurations.

12 Band alignment

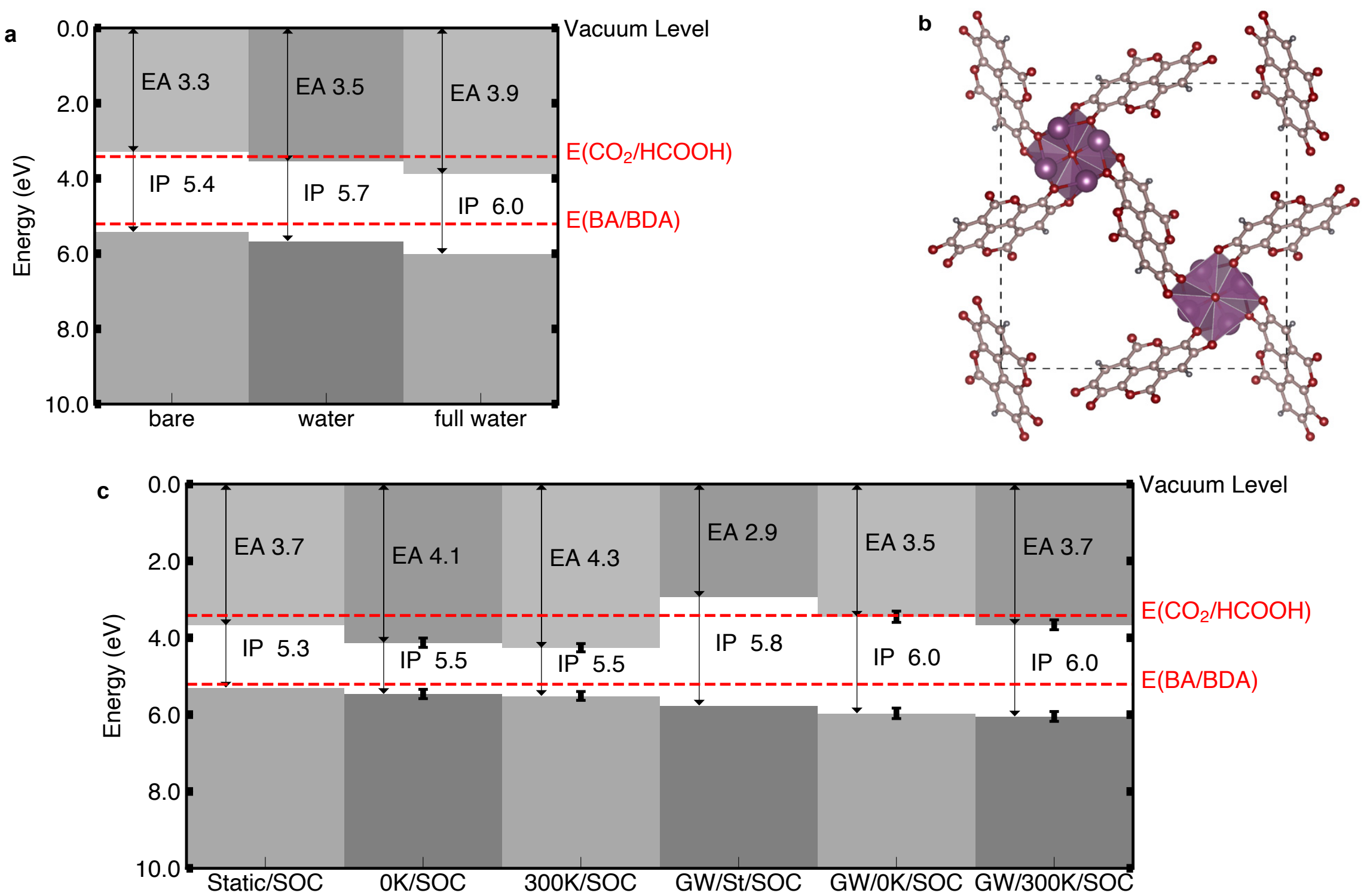

Figure S34: Band alignment to vacuum of a. SU-101 with different water loadings at the DFT level, and b. activated SU-101 for different cases at different levels of theory, including spin-orbit coupling. Redox potentials of reference are CO_2 reduction to formic acid as a case study [36], and coupling of neat benzylamine [11].

Regarding band alignment, as shown in Figure S34, phonons affect not only the electron affinity but also lower the ionization potential at the GW level. This trend is consistent across different levels of theory. While the changes in band alignment are relatively small, they can still influence SU-101's ability to drive specific redox reactions, such as CO_2 reduction reaction (CO2RR). For example, within the quasiparticle picture, SU-101$_0$ is predicted to promote CO2RR without considering electron-phonon coupling. However, when the effects of phonons at both zero point and 300 K are taken into account, SU-101 no longer aligns with the redox potential of CO2RR. This highlights the potential significance of electron-phonon interactions in tuning the photocatalytic performance of SU-101.

13 Averaged predicted optoelectronic properties

Following previous studies on electron-phonon coupling from a finite-displacement approach [37–39], the ensemble average in the adiabatic approximation can be described by Equation (1) in the Methods Section 4.2.

If the nuclear motion is assumed to be harmonic and we consider the Born-Oppenheimer approximation [39], the ensemble average $\langle \mathcal{O}^{har} \rangle$ is then simply

$$\langle \mathcal{O}^{har} \rangle = \lim_{N \to \infty} 1/N \sum_{i=1}^{N} \langle \mathcal{O}(X_i^{har}) \rangle \tag{S2}$$

Therefore, we compute the average phonon effect on the G_0W_0-BSE absorption spectra by simply averaging its values at the 10 Monte-Carlo displaced configurations that best capture the average effect of band gap renormalization at DFT-PBE level.

Below, we show the average BSE absorption spectra, $\epsilon_2(\omega)$, at 0 K and 300 K for bound water and activated SU-101.

13.1 Absorption

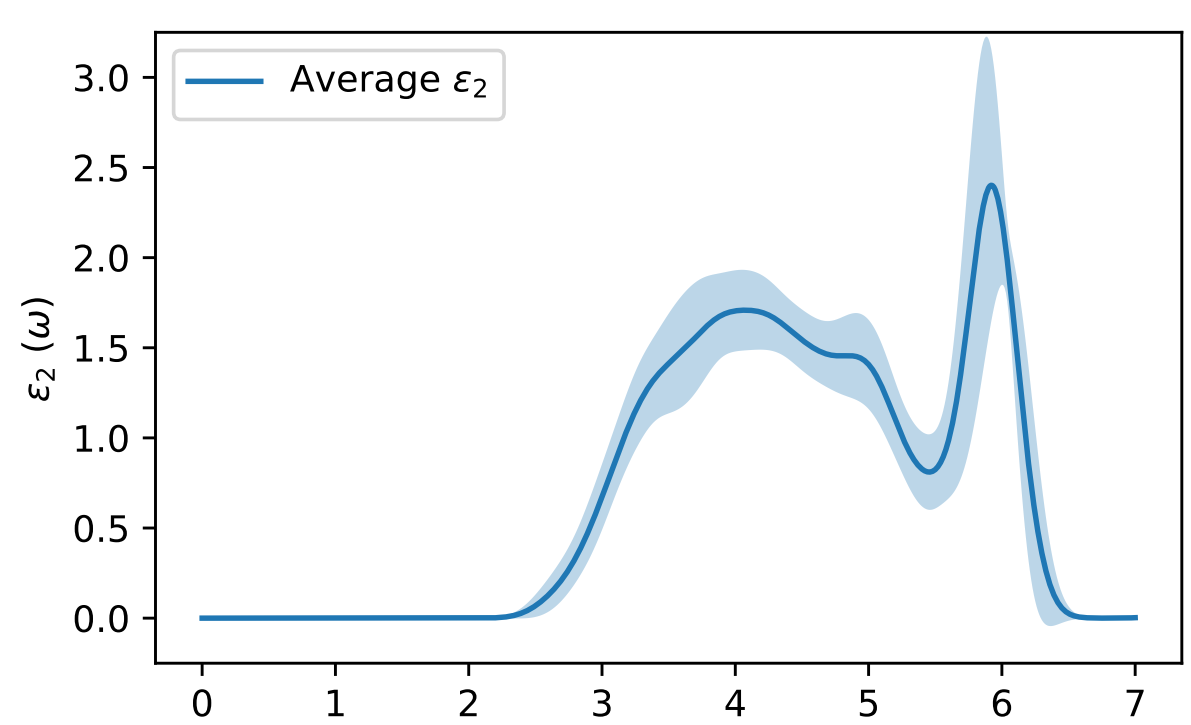

Figure S35: Absorption spectra from G_0W_0-BSE averaged over the 10 water-bound configurations at 0 K (spin up).

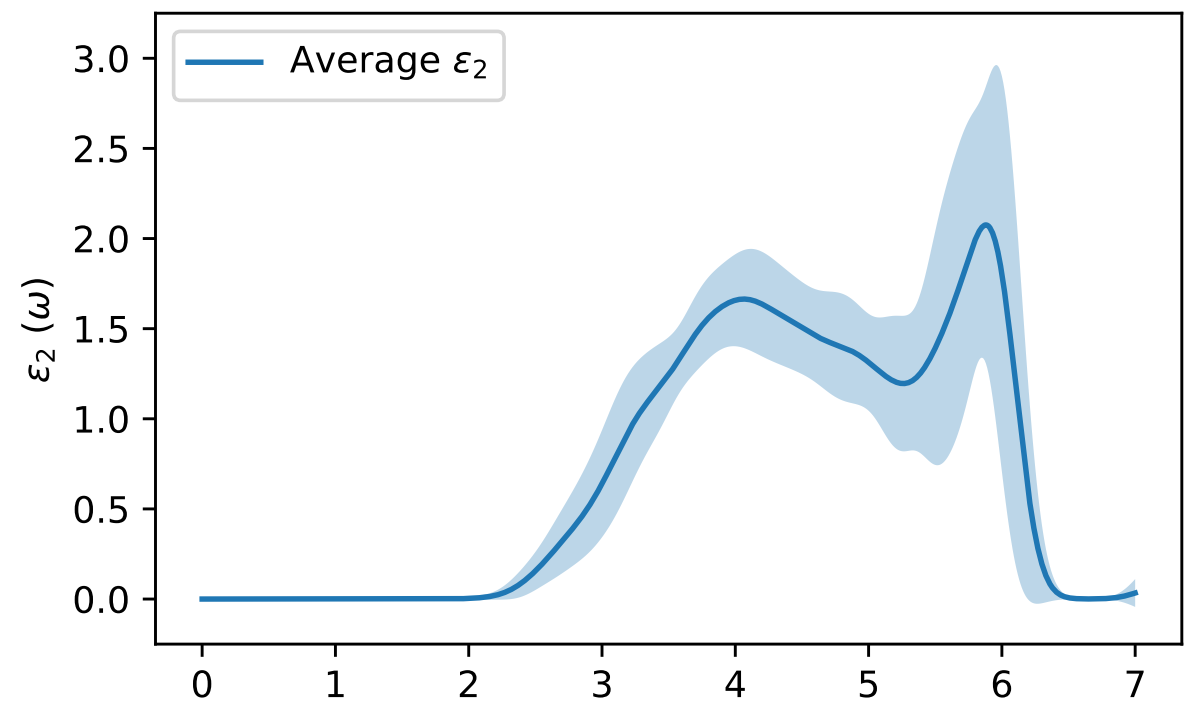

Figure S36: Absorption spectra from G_0W_0-BSE averaged over the 10 water-bound configurations at 300 K (spin up).

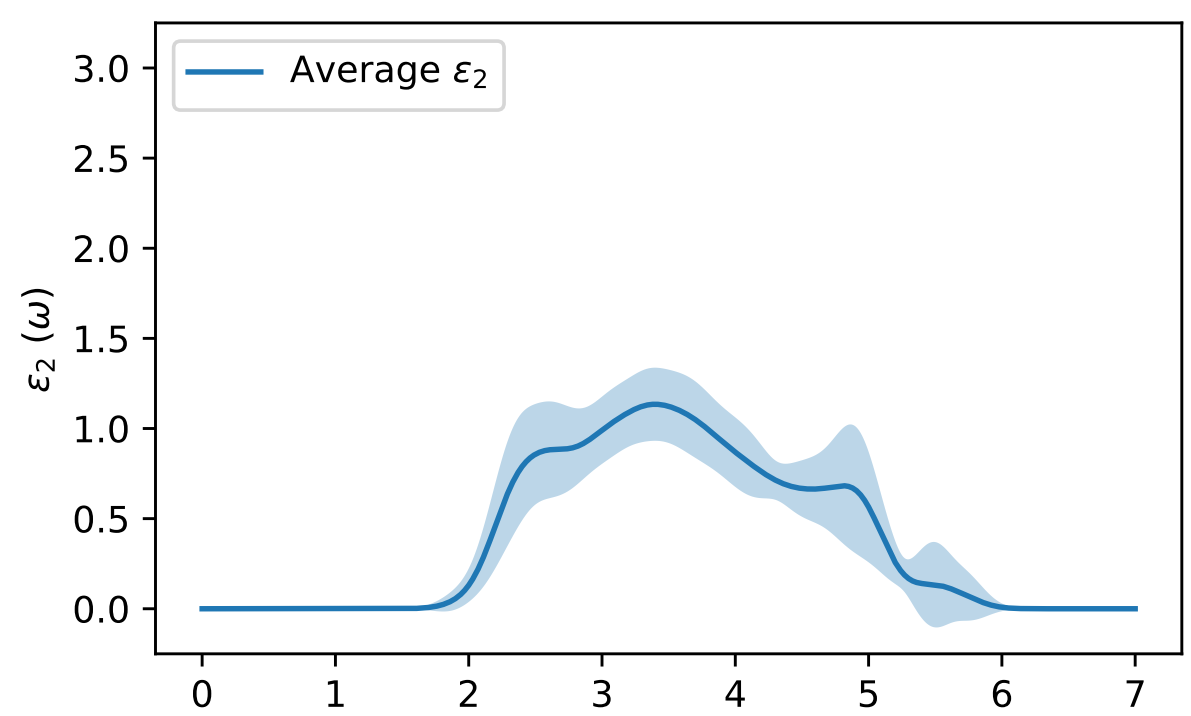

Figure S37: Absorption spectra from G_0W_0-BSE averaged over the 10 activated configurations at 0 K (spin up).

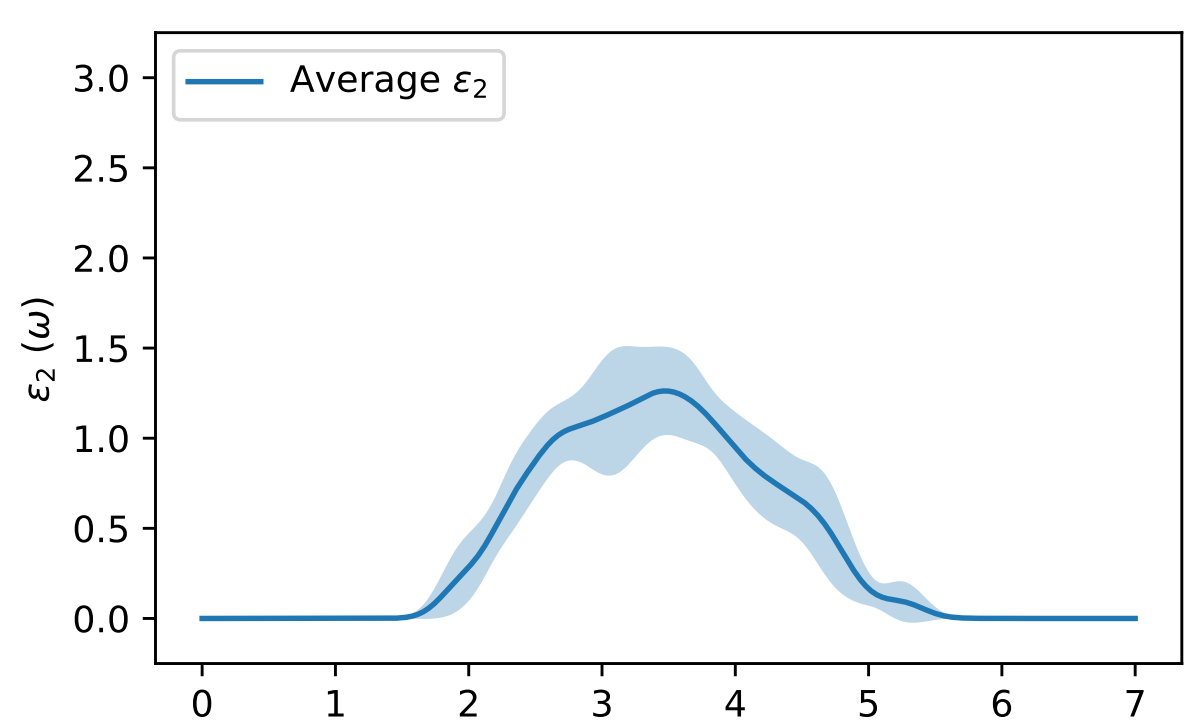

Figure S38: Absorption spectra from G_0W_0-BSE averaged over the 10 activated configurations at 300 K (spin up).

14 Planar-averaged electron-hole correlation function

The spatial extent and anisotropy of the excitonic states are analyzed in terms of the electron–hole correlation function. For a given exciton state S, this quantity may be defined as

$$F_S(\mathbf{r}) = \int_\Omega d^3\mathbf{r}_h \, \left|\Psi_S(\mathbf{r}_e = \mathbf{r} + \mathbf{r}_h, \mathbf{r}_h)\right|^2, \tag{S3}$$

where $\Psi_S(\mathbf{r}_e, \mathbf{r}_h)$ is the exciton wavefunction, $\mathbf{r}_e$ and $\mathbf{r}_h$ denote the electron and hole coordinates, respectively, and the integration is performed over all possible hole positions within the unit cell.

In the present work, rather than performing the integration over many hole positions in Equation (S3), we evaluate this function by fixing the hole position at a physically relevant site. The hole is fixed at a carbonyl oxygen atom in the organic linker, which contributes strongly to the valence-band states and is located near the geometric center of the simulation supercell. We use the `plotxct.x` utility of BerkeleyGW to evaluate the real-space electron probability density for this fixed hole position $\mathbf{r}_h = \mathbf{r}_h^0$,

$$\rho_e(\mathbf{r}_e \mid \mathbf{r}_h^0) = \left|\Psi_S(\mathbf{r}_e, \mathbf{r}_h^0)\right|^2. \tag{S4}$$

To obtain a one-dimensional, direction-resolved representation of the electron–hole correlation, we compute a planar average of F_S along a chosen Cartesian direction. Denoting this direction as $r_\parallel$ and the two perpendicular coordinates as $\mathbf{r}_\perp$, we define

$$g(r_\parallel) = \frac{\displaystyle\int d^2\mathbf{r}_\perp \, F_S(r_\parallel, \mathbf{r}_\perp)}{\displaystyle\int d^2\mathbf{r}_\perp}. \tag{S5}$$

Equation (S5) yields a normalized one-dimensional profile describing the spatial distribution of the electron along direction $r_\parallel$ for a fixed hole position, along the lines of Eq. 2

in [40].

The quantities plotted in Figures 2 and 3 correspond to $g(r_\parallel)$ evaluated along the x, y, and z directions of the simulation cell. The z axis is aligned with the metal-node chain of the MOF, while the x and y directions lie in the plane perpendicular to this axis. The range and origin in Å correspond to that of a 2x2x6 supercell, as shown in Figure 2a,b.

The spatial coordinates used to evaluate F_S and $g(r_\parallel)$ correspond to the real-space grid of the simulation supercell, with the origin defined by the Gaussian cube file coordinate system. The fixed hole position $\mathbf{r}_h^0$ lies close to the geometric center of the supercell.

14.1 Planar-averaged electron-hole correlation function at 0 K and 300 K

As described above and in Methods Section 4.4, for each exciton wavefunction and in each direction, we compute the planar-averaged electron-hole correlation function. In Figure 3, we plot this quantity averaged over the 10 phonon-displaced configurations at 0 K and 300 K, and the standard deviation is also plotted in Figures S39 and S40.

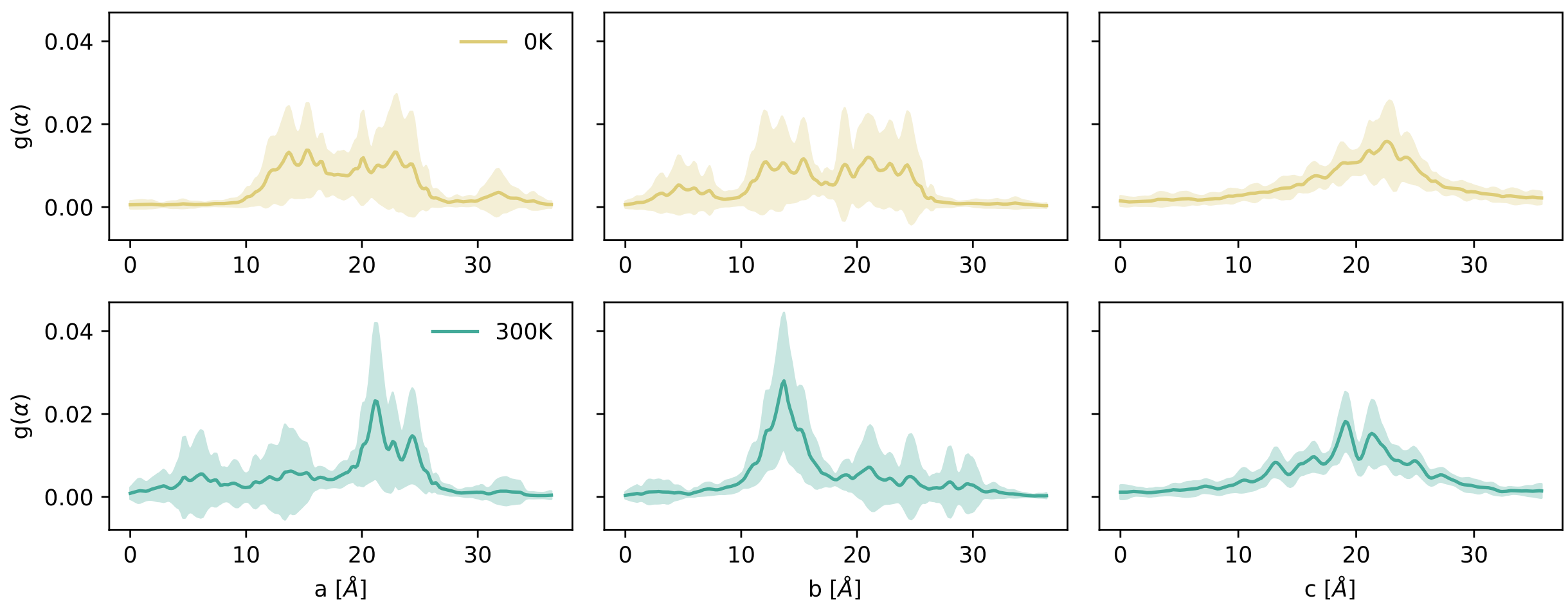

Figure S39: For water-bound SU-101 at 0 K and 300 K, we plot the planar-averaged electron-hole correlation function $g(\alpha)$ (α is the direction of interest) [40], which provides a profile of the probability of finding an electron given a hole fixed at the origin (see Methods Section 4.4, and SI Section 14). This quantity is further averaged for the 10 phonon-sampled configurations at each temperature, and the shaded area represents the standard deviation. The range and origin in Å for all lattice parameters correspond to those of the 2x2x6 supercells represented in Figure 4b.

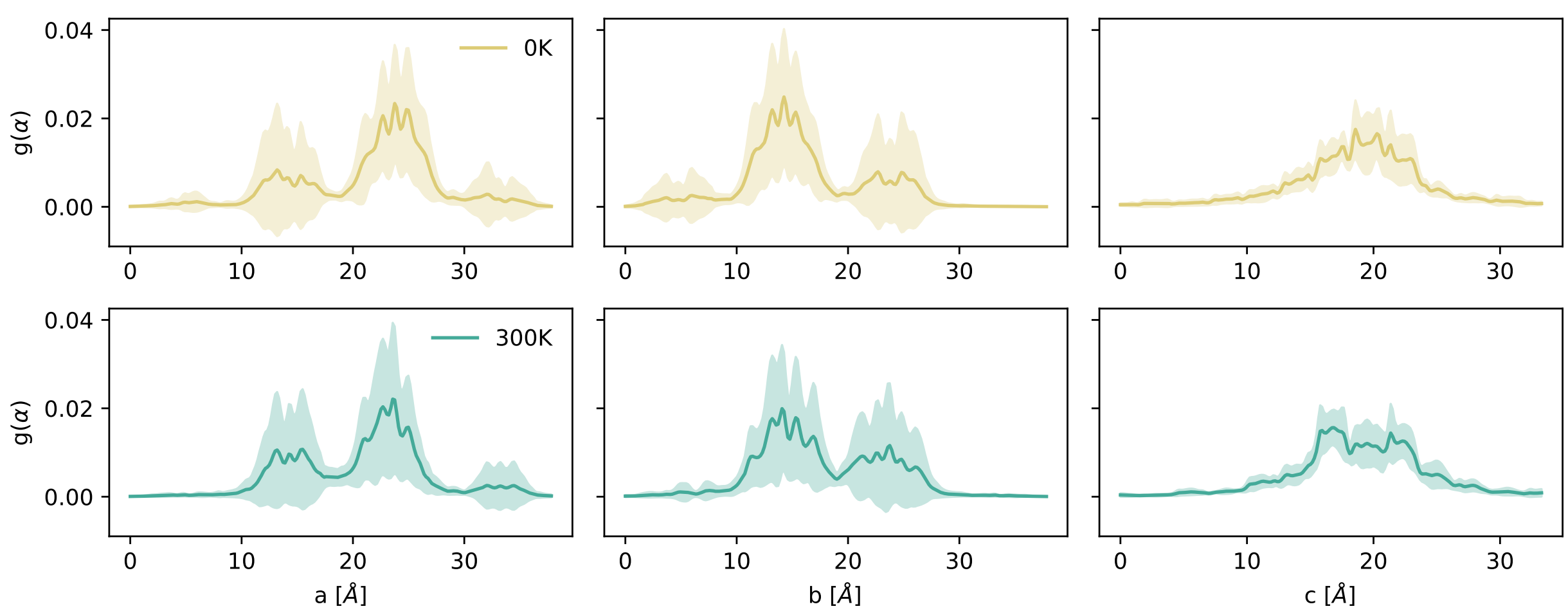

Figure S40: For activated SU-101 at 0 K and 300 K, we plot the planar-averaged electron-hole correlation function $g(\alpha)$ (α is the direction of interest) [40], which provides a profile of the probability of finding an electron given a hole fixed at the origin (see Methods Section 4.4, and SI Section 14). This quantity is further averaged for the 10 phonon-sampled configurations at each temperature, and the shaded area represents the standard deviation. The range and origin in Å for all lattice parameters correspond to those of the 2x2x6 supercells represented in Figure 4a.

15 Experimental Discussion

The permanent microporosity of the structure is also evaluated via N_2 adsorption isotherms at 77 K after solvent exchange and different activation conditions under dynamic vacuum. The measurements show a typical type I isotherm (Figure S41). The total pore volumes and BET surface areas are summarized in Table S3. The highest surface area and pore volume (i.e., 353 m^2g^{-1}, and 0.16 cm^3g^{-1}, respectively), were obtained with activation conditions of 150 °C for 12 hours and solvent exchange with MeOH, which were then used as activation conditions in this study. Although very similar activation conditions were used compared to the original publication (i.e., 150 °C for 10 hours), the surface area obtained in this study is still slightly lower. As noted in the original publication, variations in surface area may be attributed to differences in ligand purity—specifically, whether reagent-grade or supplement-grade ellagic acid was used [1]. The previously reported ligand had a purity of 97%, whereas the one used in this study was $\geq$ 95%, which may explain the slightly lower surface area observed in this study.

We performed additional PXRD measurements at 200 °C under dynamic vacuum, without a prior solvent-exchange step, to investigate whether increasing the temperature causes further changes in the PXRD patterns. PXRD measurements confirm that the framework remains crystalline after activation at 200 °C. However, its BET surface area is lower than that of solvent-exchanged samples, even if those were activated at 150 °C. This trend is consistent in both our measurements and the original report on SU-101, where solvent exchange, rather than activation temperature, was identified as the key step for achieving high accessible surface area. These results confirm that the structural and optical measurements after solvent exchange and activation at 150 °C are representative of the activated form of SU-101.

Thermogravimetric analysis data of the as-made structure of SU-101 was carried out. The sample was heated under air conditions from room temperature to 700 °C to allow for its decomposition into its metal oxide (i.e., Bi_2O_3). As illustrated in Figure S42, the formula that best matched the observed data was determined to be $Bi_2O(H_2O)_2(C_{14}H_2O_8) \cdot 2\ H_2O$,

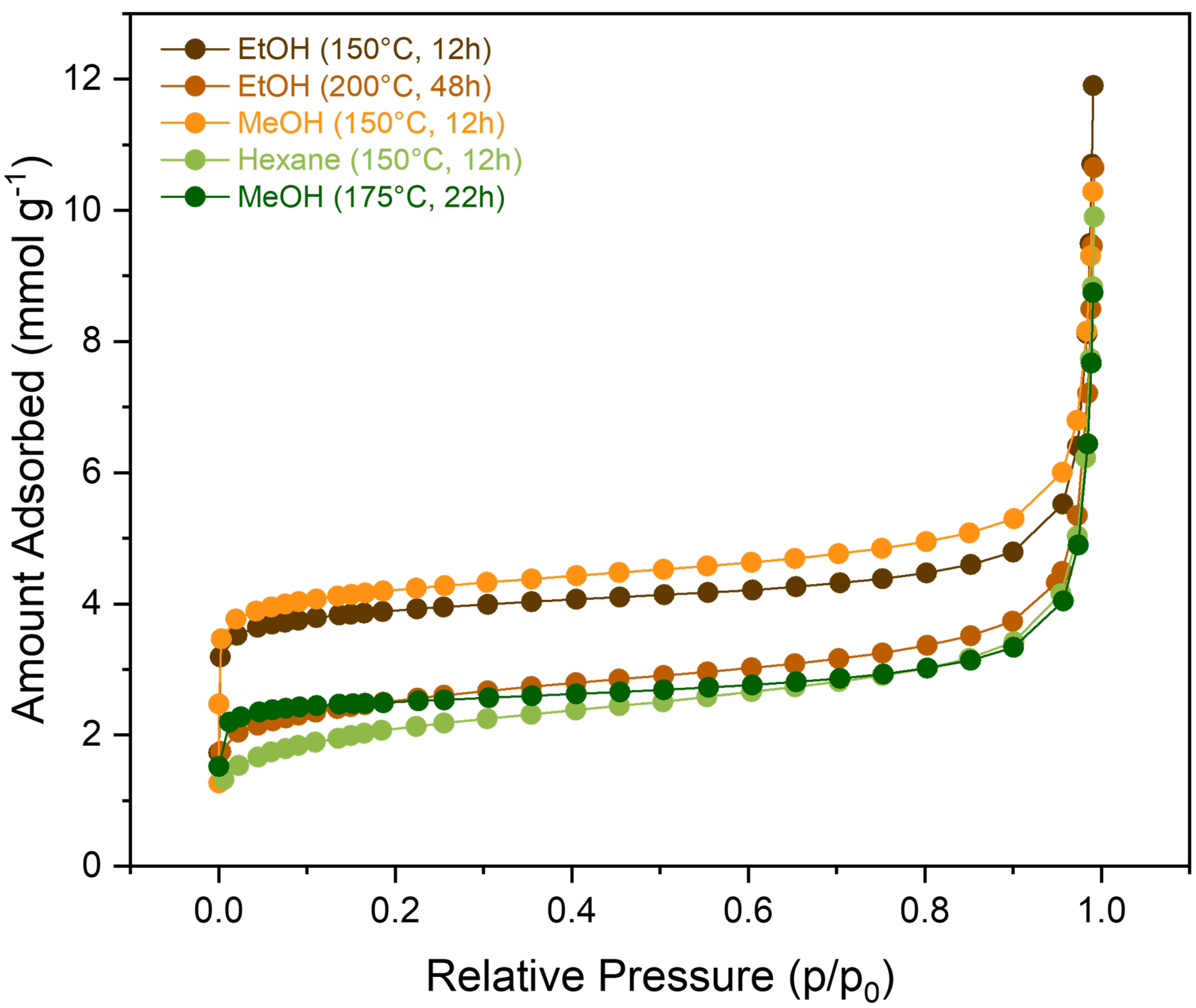

Figure S41: N_2 adsorption isotherms at 77 K of SU-101 activated under different conditions.

Table S3: BET surface areas and pore volumes of SU-101 activated at different conditions.

**Activation Conditions**	**Solvent Exchange (∼ 3 days)**	**BET Surface Area (m^2 g^{-1})**	**Pore Volume (at $p/p_0 = 0.5$, cm^3 g^{-1})**
**150 °C, 12 hours**	**Hexane**	168	0.09
	**MeOH**	353	0.16
	**EtOH**	333	0.14
**175 °C, 22 hours**	**MeOH**	215	0.09
**200 °C, 48 hours**	**EtOH**	247	0.10

accounting for 2 coordinated H_2O molecules and 2 non-coordinated H_2O molecules in the pores.

Elemental analysis on the same sample was also performed. The theoretical values calculated for the chemical formula: $Bi_2O(H_2O)_2(C_{14}H_2O_8) \cdot 2\,H_2O$ are in very good agreement

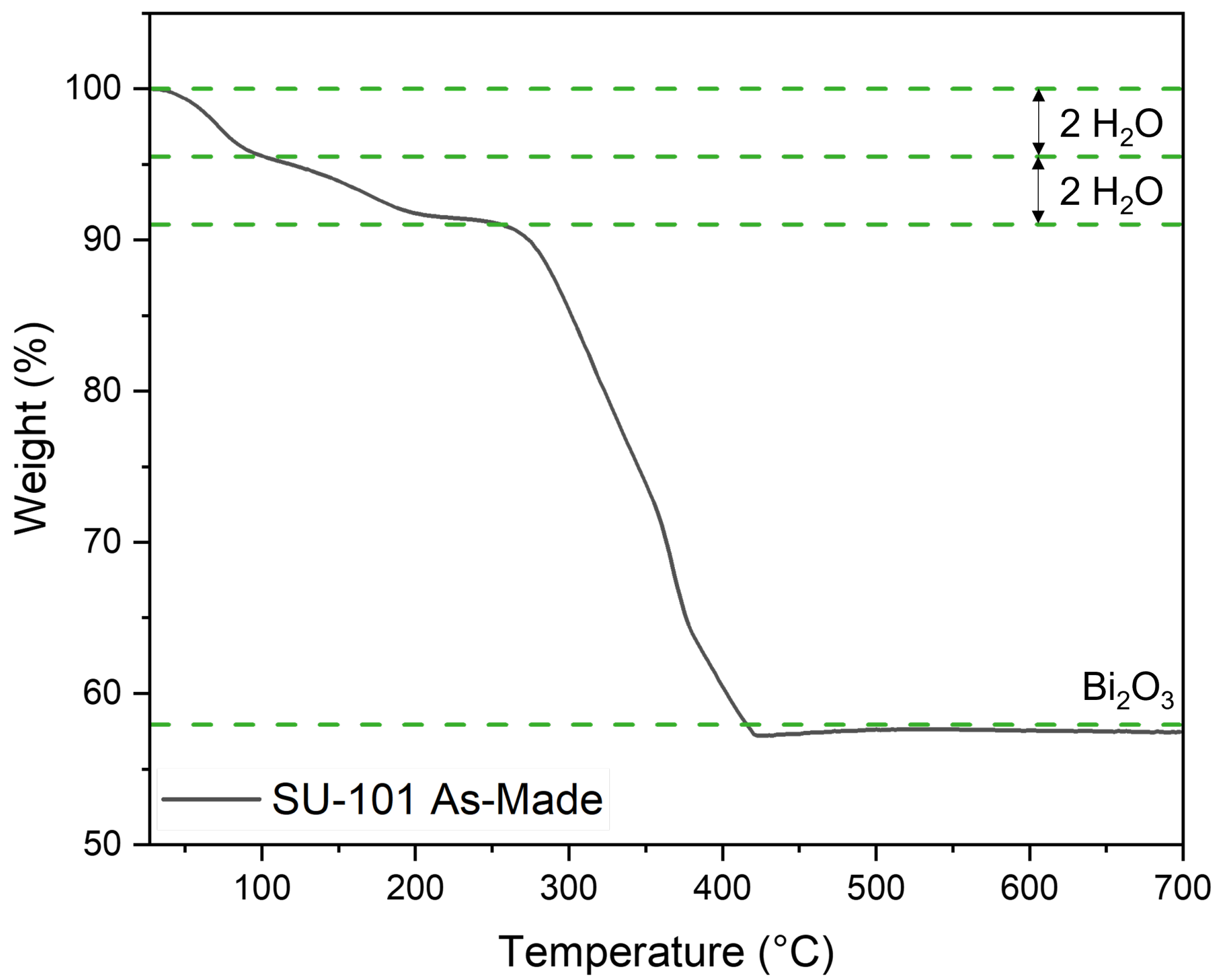

Figure S42: Thermogravimetric measurement of SU-101 in air. The green, dashed lines indicate the expected relative mass remaining, assuming an initial formula of $Bi_2O(H_2O)_2(C_{14}H_2O_8) \cdot 2\ H_2O$, which is then converted into Bi_2O_3, when heated beyond 400 °C.

with the experimental values (Table S4), supporting the phase purity of the sample determined by PXRD and the Le Bail fits.

Table S4: Elemental analysis (EA) of the as-made material of SU-101, with the theoretical values calculated based on the formula: $Bi_2O(H_2O)_2(C_{14}H_2O_8) \cdot 2\ H_2O$.

**EA**	**SU-101 As-Made**	
**Content**	**Theory**	**Experimental**
**%C**	20.91	20.46 ± 0.04
**%H**	1.25	1.31 ± 0.04
**%N**	0.01	0.01 ± 0.01

Fourier-Transform Infrared spectroscopy (FT-IR) experiments were used to confirm the

successful coordination interaction between ellagic acid and bismuth in SU-101 (Figure S43). The bands at 3474 and 1510 cm^{-1}, which can be attributed to the phenolic O-H stretching and bending modes, respectively, in ellagic acid, disappeared in the as-made material of SU-101, confirming the successful chelation of bismuth through this functional group [41]. A broad band observed at 3149 cm^{-1}, in ellagic acid, is assigned to O-H stretching vibrations. In SU-101, this band shifts to higher frequencies (i.e., 3420 cm^{-1}), which may be attributed to weaker hydrogen bonding interactions in SU-101 compared to the free ellagic acid ligand.

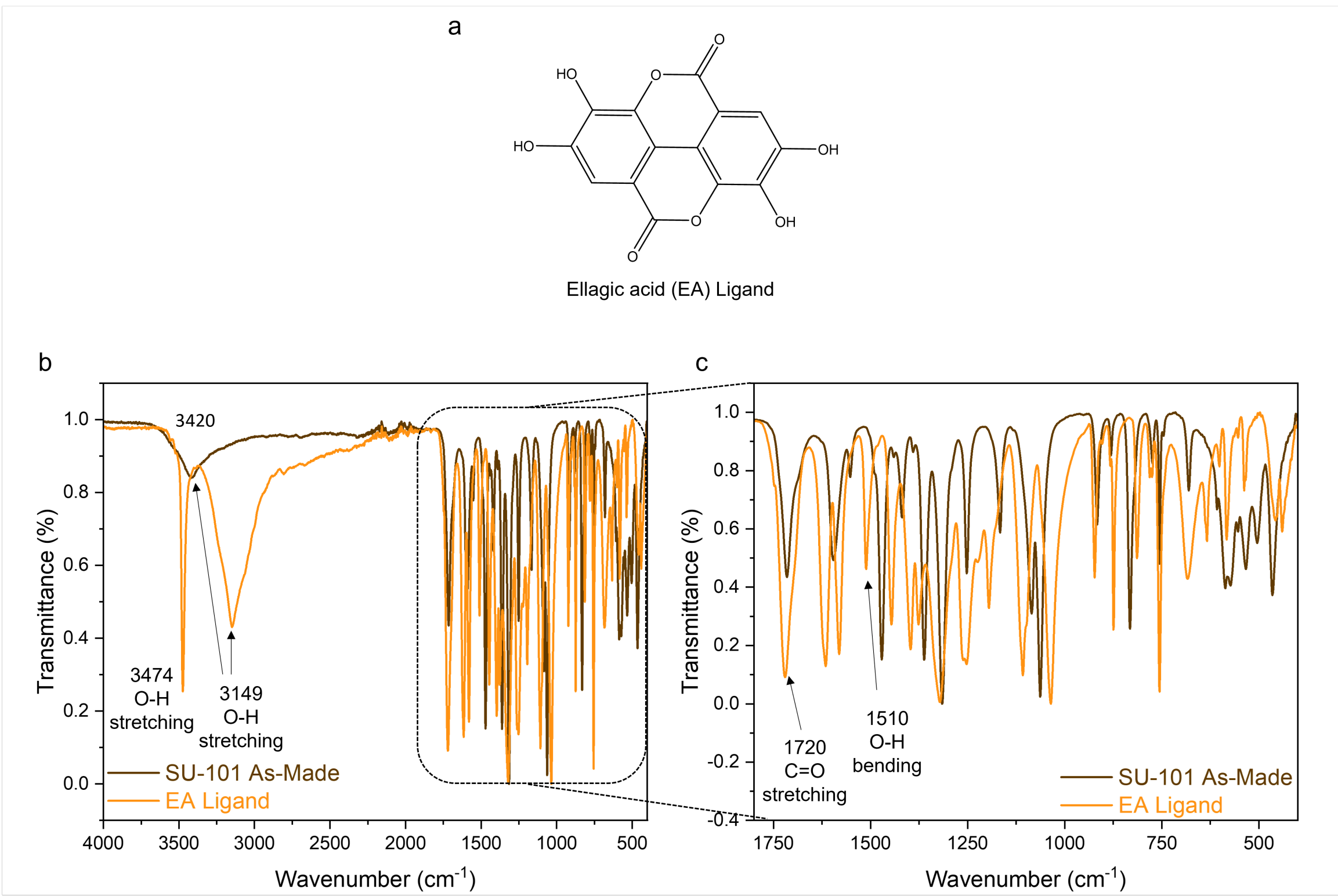

Figure S43: **a)** Chemical structure of the ellagic acid (EA) ligand. **b)** FT-IR spectra of the as-made SU-101 (brown) and the EA ligand (yellow). **c)** Zoomed-in FT-IR spectra of the as-made material and ligand.

FT-IR experiments were also conducted on SU-101 before and after activation to explore the possible changes on the characteristic O-H and C=O stretching and Bi-O vibration bands due to water removal (Figure S44). The almost inexistent O-H stretching mode in the activated sample of SU-101 suggests almost complete activation of the material at 150 °C, for 12 hours. The bands associated with C=O and C=C stretching [42] also shift towards

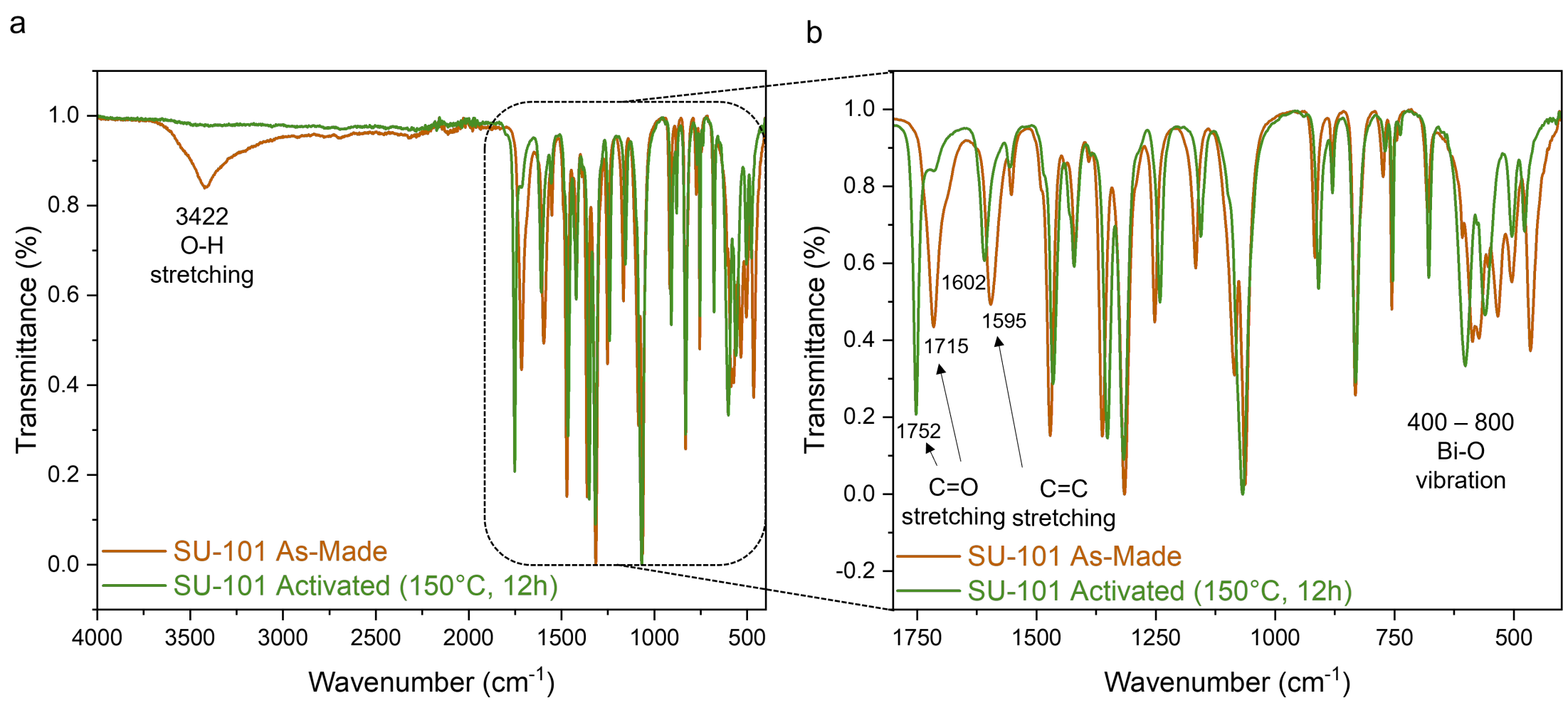

Figure S44: **a)** FT-IR spectra of the as-made SU-101 (brown) and activated SU-101 (green). **b)** Zoomed-in FT-IR spectra of the as-made and activated materials.

higher frequencies upon activation (1715 vs 1752 cm^{-1}, and 1595 vs 1602 cm^{-1}, respectively), which may be attributed to the loss of hydrogen bonding interactions as water molecules are removed, leading to stronger bond stiffness and higher vibrational frequencies. The bands located between 400-800 cm^{-1} are associated with the Bi-O bond [43]. In particular, the bands between 460 and 600 cm^{-1} shift toward higher frequencies upon activation, which is a good indication that at least some coordinated water molecules have been removed.

References

[1] E. S. Grape, J. G. Flores, T. Hidalgo, E. Martínez-Ahumada, A. Gutiérrez-Alejandre, A. Hautier, D. R. Williams, M. O'Keeffe, L. Öhrström, T. Willhammar, P. Horcajada, I. A. Ibarra, and A. K. Inge, "A robust and biocompatible bismuth ellagate mof synthesized under green ambient conditions," *Journal of the American Chemical Society*, vol. 142, p. 16795–16804, Sept. 2020.

[2] Y. Fan, W. Zhou, X. Qiu, H. Li, Y. Jiang, Z. Sun, D. Han, L. Niu, and Z. Tang, "Selective photocatalytic oxidation of methane by quantum-sized bismuth vanadate," *Nature Sustainability*, vol. 4, p. 509–515, Feb. 2021.

[3] T. W. Kim and K.-S. Choi, "Nanoporous bivo 4 photoanodes with dual-layer oxygen evolution catalysts for solar water splitting," *Science*, vol. 343, p. 990–994, Feb. 2014.

[4] P. Riente, M. Fianchini, P. Llanes, M. A. Pericàs, and T. Noël, "Shedding light on the nature of the catalytically active species in photocatalytic reactions using bi2o3 semiconductor," *Nature Communications*, vol. 12, Jan. 2021.

[5] J. L. Obeso, J. Gabriel Flores, C. V. Flores, R. Rios-Escobedo, J. Aguilar-Pliego, A. Ken Inge, J. Antonio de los Reyes, R. A. Peralta, I. A. Ibarra, and C. Leyva, "Lewis acid-catalyzed ring-opening alcoholysis of cyclohexene oxide: The role of open metal sites in the bi(iii)-based metal-organic framework su-101," *ChemCatChem*, vol. 15, June 2023.

[6] O. J. Scherer, "Phosphorus, arsenic, antimony, and bismuth multiply bonded systems with low coordination number — their role as complex ligands," *Angewandte Chemie International Edition in English*, vol. 24, p. 924–943, Nov. 1985.

[7] H. Bhatia, J. Guo, C. N. Savory, M. Rush, D. I. James, A. Dey, C. Chen, D.-K. Bučar, T. M. Clarke, D. O. Scanlon, R. G. Palgrave, and B. C. Schroeder, "Exploring

bismuth coordination complexes as visible-light absorbers: Synthesis, characterization, and photophysical properties," *Inorganic Chemistry*, vol. 63, p. 416–430, Dec. 2023.

[8] L. A. Maurer, O. M. Pearce, F. D. R. Maharaj, N. L. Brown, C. K. Amador, N. H. Damrauer, and M. P. Marshak, "Open for bismuth: Main group metal-to-ligand charge transfer," *Inorganic Chemistry*, vol. 60, p. 10137–10146, June 2021.

[9] H. Wang, X. Xu, C. Lee, C. Johnson, K. Sohlberg, and H.-F. Ji, "Highly selective sensing of nitroaromatics using nanomaterials of ellagic acid," *The Journal of Physical Chemistry C*, vol. 116, p. 4442–4448, Feb. 2012.

[10] E. Bittrich, J. Domke, D. Jehnichen, L. Bittrich, M. Malanin, A. Janke, P. Uhlmann, K.-J. Eichhorn, A. Papamichail, V. Stanishev, V. Darakchieva, M. Al-Hussein, M. Levichkova, T. Fritz, and K. Walzer, "Morphology of thin films of aromatic ellagic acid and its hydrogen bonding interactions," *The Journal of Physical Chemistry C*, vol. 124, p. 16381–16390, July 2020.

[11] R. H. Alzard, L. A. Siddig, A. S. Abdelhamid, and A. Alzamly, "Visible-light-driven photocatalytic coupling of neat benzylamine over a bi-ellagate metal–organic framework," *ACS Omega*, vol. 7, p. 36689–36696, Oct. 2022.

[12] A. J. Chacón-García, S. Rojas, E. S. Grape, F. Salles, T. Willhammar, A. K. Inge, Y. Pérez, and P. Horcajada, "Su-101 for the removal of pharmaceutical active compounds by the combination of adsorption/photocatalytic processes," *Scientific Reports*, vol. 14, Apr. 2024.

[13] Q. Zhang, Y. Liu, Z. Wang, P. Wang, Z. Zheng, H. Cheng, X. Qin, X. Zhang, Y. Dai, and B. Huang, "A biocompatible bismuth based metal-organic framework as efficient light-sensitive drug carrier," *Journal of Colloid and Interface Science*, vol. 617, p. 578–584, July 2022.

[14] B. Mourino, K. M. Jablonka, A. Ortega-Guerrero, and B. Smit, "In search of covalent organic framework photocatalysts: A dft-based screening approach," *Advanced Functional Materials*, vol. n/a, no. n/a, p. 2301594, 2023.

[15] S. P. Huber, S. Zoupanos, M. Uhrin, L. Talirz, L. Kahle, R. Häuselmann, D. Gresch, T. Müller, A. V. Yakutovich, C. W. Andersen, F. F. Ramirez, C. S. Adorf, F. Gargiulo, S. Kumbhar, E. Passaro, C. Johnston, A. Merkys, A. Cepellotti, N. Mounet, N. Marzari, B. Kozinsky, and G. Pizzi, "AiiDA 1.0, a scalable computational infrastructure for automated reproducible workflows and data provenance," *Scientific Data*, vol. 7, Sept. 2020.

[16] M. Uhrin, S. P. Huber, J. Yu, N. Marzari, and G. Pizzi, "Workflows in AiiDA: Engineering a high-throughput, event-based engine for robust and modular computational workflows," *Computational Materials Science*, vol. 187, p. 110086, Feb. 2021.

[17] T. D. Kühne, M. Iannuzzi, M. Del Ben, V. V. Rybkin, P. Seewald, F. Stein, T. Laino, R. Z. Khaliullin, O. Schütt, F. Schiffmann, D. Golze, J. Wilhelm, S. Chulkov, M. H. Bani-Hashemian, V. Weber, U. Borštnik, M. Taillefumier, A. S. Jakobovits, A. Lazzaro, H. Pabst, T. Müller, R. Schade, M. Guidon, S. Andermatt, N. Holmberg, G. K. Schenter, A. Hehn, A. Bussy, F. Belleflamme, G. Tabacchi, A. Glöß, M. Lass, I. Bethune, C. J. Mundy, C. Plessl, M. Watkins, J. VandeVondele, M. Krack, and J. Hutter, "Cp2k: An electronic structure and molecular dynamics software package - quickstep: Efficient and accurate electronic structure calculations," *The Journal of Chemical Physics*, vol. 152, no. 19, p. 194103, 2020.

[18] A. M. Ganose, A. J. Jackson, and D. O. Scanlon, "sumo: Command-line tools for plotting and analysis of periodic *ab initio* calculations," *Journal of Open Source Software*, vol. 3, no. 28, p. 717, 2018.

[19] K. T. Butler, C. H. Hendon, and A. Walsh, "Electronic chemical potentials of porous metal–organic frameworks," *Journal of the American Chemical Society*, vol. 136, no. 7, pp. 2703–2706, 2014. PMID: 24447027.

[20] K. M. Jablonka, "Cube analysis." `https://github.com/kjappelbaum/cubes`. Accessed: 2022-09-17.

[21] M. Fumanal, A. Ortega-Guerrero, K. M. Jablonka, B. Smit, and I. Tavernelli, "Charge separation and charge carrier mobility in photocatalytic metal-organic frameworks," *Adv. Funct. Mater.*, vol. 30, no. 49, p. 2003792, 2020.

[22] M. Fumanal, G. Capano, S. Barthel, B. Smit, and I. Tavernelli, "Energy-based descriptors for photo-catalytically active metal-organic framework discovery," *Journal of Materials Chemistry A*, vol. 8, no. 8, pp. 4473–4482, 2020.

[23] T. Kamencek, N. Bedoya-Martínez, and E. Zojer, "Understanding phonon properties in isoreticular metal-organic frameworks from first principles," *Physical Review Materials*, vol. 3, Nov. 2019.

[24] P. Ghosh, A. M. Alvertis, R. Chowdhury, P. Murto, A. J. Gillett, S. Dong, A. J. Sneyd, H.-H. Cho, E. W. Evans, B. Monserrat, F. Li, C. Schnedermann, H. Bronstein, R. H. Friend, and A. Rao, "Decoupling excitons from high-frequency vibrations in organic molecules," *Nature*, vol. 629, p. 355–362, May 2024.

[25] F. Karsai, M. Engel, E. Flage-Larsen, and G. Kresse, "Electron–phonon coupling in semiconductors within the gw approximation," *New Journal of Physics*, vol. 20, p. 123008, dec 2018.

[26] M. Adams, M. Kozlowska, N. Baroni, M. Oldenburg, R. Ma, D. Busko, A. Turshatov, G. Emandi, M. O. Senge, R. Haldar, *et al.*, "Highly efficient one-dimensional triplet exciton transport in a palladium–porphyrin-based surface-anchored metal–organic framework," *ACS Applied Materials & Interfaces*, vol. 11, no. 17, pp. 15688–15697, 2019.

[27] J. Deslippe, G. Samsonidze, D. A. Strubbe, M. Jain, M. L. Cohen, and S. G. Louie, "Berkeleygw: A massively parallel computer package for the calculation of the quasi-particle and optical properties of materials and nanostructures," *Computer Physics Communications*, vol. 183, p. 1269–1289, June 2012.

[28] M. Rohlfing and S. G. Louie, "Electron-hole excitations and optical spectra from first principles," *Physical Review B*, vol. 62, p. 4927–4944, Aug. 2000.

[29] M. S. Hybertsen and S. G. Louie, "Electron correlation in semiconductors and insulators: Band gaps and quasiparticle energies," *Physical Review B*, vol. 34, p. 5390–5413, Oct. 1986.

[30] D. Y. Qiu, F. H. da Jornada, and S. G. Louie, "Screening and many-body effects in two-dimensional crystals: Monolayer mos2," *Physical Review B*, vol. 93, June 2016.

[31] Z. Tao, J. B. Haber, and J. B. Neaton, "Real-space quantification of exciton localization in acene crystals using wannier function decomposition," 2025.

[32] M. Schlipf and F. Giustino, "Dynamic rashba-dresselhaus effect," *Physical Review Letters*, vol. 127, Dec. 2021.

[33] B. Monserrat and D. Vanderbilt, "Phonon-assisted spin splitting in centrosymmetric crystals," 2017.

[34] J. A. Steele, P. Puech, B. Monserrat, B. Wu, R. X. Yang, T. Kirchartz, H. Yuan, G. Fleury, D. Giovanni, E. Fron, M. Keshavarz, E. Debroye, G. Zhou, T. C. Sum, A. Walsh, J. Hofkens, and M. B. J. Roeffaers, "Role of electron–phonon coupling in the thermal evolution of bulk rashba-like spin-split lead halide perovskites exhibiting dual-band photoluminescence," *ACS Energy Letters*, vol. 4, p. 2205–2212, Aug. 2019.

[35] B. A. Barker, J. Deslippe, J. Lischner, M. Jain, O. V. Yazyev, D. A. Strubbe, and S. G. Louie, "Spinor gw/bethe-salpeter calculations in berkeleygw: Implementation, symmetries, benchmarking, and performance," *Physical Review B*, vol. 106, Sept. 2022.

[36] D. K. Kanan and E. A. Carter, "Band gap engineering of mno via zno alloying: A potential new visible-light photocatalyst," *The Journal of Physical Chemistry C*, vol. 116, p. 9876–9887, Apr. 2012.

[37] B. Monserrat and R. J. Needs, "Comparing electron-phonon coupling strength in diamond, silicon, and silicon carbide: First-principles study," *Physical Review B*, vol. 89, June 2014.

[38] B. Monserrat, "Vibrational averages along thermal lines," *Physical Review B*, vol. 93, Jan. 2016.

[39] A. M. Alvertis, J. B. Haber, E. A. Engel, S. Sharifzadeh, and J. B. Neaton, "Phonon-induced localization of excitons in molecular crystals from first principles," *Physical Review Letters*, vol. 130, Feb. 2023.

[40] S. Sharifzadeh, P. Darancet, L. Kronik, and J. B. Neaton, "Low-energy charge-transfer excitons in organic solids from first-principles: The case of pentacene," *The Journal of Physical Chemistry Letters*, vol. 4, p. 2197–2201, June 2013.

[41] R. H. Alzard, L. A. Siddig, A. S. Abdelhamid, and A. Alzamly, "Visible-light-driven photocatalytic coupling of neat benzylamine over a bi-ellagate metal–organic framework," *ACS omega*, vol. 7, no. 41, pp. 36689–36696, 2022.

[42] S.-J. Lee, B.-S. Cheong, and H.-G. Cho, "Surface-enhanced raman spectroscopic studies of ellagic acid in silver colloids," *Bulletin of the Korean Chemical Society*, vol. 36, no. 6, pp. 1637–1644, 2015.

[43] X. Liu, Y. Su, Q. Zhao, C. Du, and Z. Liu, "Constructing bi24o31cl10/biocl heterojunction via a simple thermal annealing route for achieving enhanced photocatalytic activity and selectivity," *Scientific reports*, vol. 6, no. 1, p. 28689, 2016.
