# Supplementary material for: Hydration-dependent exciton-phonon coupling in a metal-organic framework photocatalyst
Source: Nat Commun. 2026 Aug 5;17:9423. doi: 10.1038/s41467-026-76097-z (PMC13538642; doi:10.1038/s41467-026-76097-z)
Supplement: Supplementary file 2 [file 41467_2026_76097_MOESM2_ESM.pdf]

nature portfolio

Peer Review File

Hydration-dependent exciton-phonon coupling in a metal-organic framework photocatalyst

Corresponding Author: Professor Berend Smit

This file contains all reviewer reports in order by version, followed by all author rebuttals in order by version.

Version 0:

Reviewer comments:

Reviewer #1

(Remarks to the Author)
This work derives the conclusion—via theoretical calculations—that phonon can induce exciton localization, with this effect being particularly pronounced in water-bound SU-101. This is a compelling finding, and research in this direction is indeed relatively scarce. However, the guiding value of this work for experimental studies, the experimental phenomena it can explain, and the scientific problems behind technical bottlenecks it can address are core priorities. Please clarify the significance of this work, or contextualize it with experimental questions. Other specific questions also need to be answered.
1. In photocatalysis, excitons in catalysts are typically expected to be delocalized (to facilitate photogenerated charge separation for separate oxidation/reduction reactions). Yet this work shows that phonons—especially in water-bound MOF catalysts—drive exciton localization (with enhanced localization at elevated temperatures), which would seem to hinder photocatalytic redox processes. However, many photothermal processes in experiments actually boost photocatalytic performance. How can this contradiction be explained from the perspective of phonon-exciton interactions?
2. Is the conclusion obtained here generalizable to MOF materials as a class?
3. The experimental observation signal is not provided in Figure S1.
4. Theoretical calculations indicate that the band gap of SU-101 decreases upon dehydration—has this phenomenon been experimentally verified?
5. For the model construction in Figure 1b, what is the basis for determining atomic coordinates, ligand connectivity, and water molecule positions? Why does water incorporation lead to lattice contraction along the a/b directions but expansion along the c direction?
6. How is the shift of the first XRD peak (Figure 1a) attributed to c-axis expansion? It is recommended to label the Miller indices corresponding to each XRD peak to clarify this correlation.
7. What is the isosurface value used in Figures 2a and 2b?
8. Why do phonons induce exciton localization in water-bound systems? Is this effect related to changes in the dielectric constant?
9. How were the phonons in Figure 5 obtained?

Reviewer #2

(Remarks to the Author)
The authors present a paper on "Hydration-dependent exciton-phonon coupling in a metal-organic framework photocatalyst, the current manuscript needs improve before it should be considered for publication.
1. Thermodynamic stability of MOFs in reported applications should be evaluated and discussed.
2. In the introduction, it should be more discussed detailed about the difference of MOFs with other metallic compounds for electron-phonon coupling,
3. Please explain more about the role of structure in electron-phonon coupling with a mechanism. What is the role of the ligand and metal?
4. What are the advantages of choosing a certain MOF? Which metal is more suitable for which type of MOF?
5. The drawbacks and challenges are suggested also be discussed.
6. Some studies on stable MOFs can be addressed, such as Energy Environ. Sci., 2025,18, 7733.
7. The individual contributions of each metal and ligand to the electron-phonon coupling should be discussed in greater detail.

Reviewer #3

(Remarks to the Author)
In this manuscript "Hydration-dependent exciton-phonon coupling in a metal-organic framework photocatalyst", the authors present a study combining GW-BSE calculations, DFT structural analysis, and experimental UV-Vis photoluminescence measurements to investigate the role of hydration in modulating the excitonic and properties of SU-101 MOF. I belive that the work is impactful in the field of MOF light-harvesting and photocatalysis. The supplementary material is clear and provides additional experimental details, including data treated and processing procedures. However, the manuscript requires careful editing for consistency, and formatting before it can be considered end version for publication. Both "major" and minor conceptual and formatting issues are present.

1) In the abstract and introduction sections, the sentence “ab initio GW and Bethe-Salpether equation” should be corrected to “GW approximation and Bethe–Salpeter equation (BSE)”, and the spelling of “Salpether” should be corrected throughout the manuscript, please check it.

2) Statements such as “the latter in excellent agreement with our computational predictions” are overly strong and should be replaced with “in good agreement.” as recommendation.

3) Sentences like “The excellent agreement with experimental trends…” overstate the results and should be moderated. Please avoid use of this type of structure.

4) In addition, some statements in the manuscript sounds "speculative". For example, the sentence “it is expected that excitons are also strongly bound” could be more accurately phrased as “Previous studies suggest relatively exciton binding energies in MOFs due to...”.

5) Results section (page 4); the authors provide useful structural and functional context for SU-101 and highlights its relevance. However, the beginning of this discussion reads more like an extended background than an analysis of results. I recommend to revise this section to focus more clearly on presenting and interpreting the data.

6) In addition, several formatting and consistency issues also need attention. For instance, energy units should be consistently reported as “eV” rather than “eVs”, and dashes should be used correctly. Please check it in all manuscript.

7) Againd, subjective phrases such as “Interestingly” or “proves crucial” should be avoided. Spelling nomenclature should also be consistent, for example “1,4-benzo dicarboxylic acid (Pag. 6)”. Terminology for photoluminescence measurements should be standardized, using either “photoemission luminescence spectra” or “photoluminescence spectra” consistently. Check it.

8) In the synthesis of SU-101, enter the percent yield of the compound. Why is the specific concentration value for the suspension in the UV-Vis absorption spectra approximately 1.24 µmol in 3 mL? The authors could add a comment for further clarification.

10) Regarding the experimental discussion, the authors report structural analysis after activation at 150 °C for 12 h under dynamic vacuum (“...The stability of the structure is also evaluated after activation at 150 °C for 12 hours, showing still a crystalline powder”). However, according to the thermogravimetric analysis (Supplementary Material, Figure S12), the release of coordinated water occurs close to 200 °C. The fully dehydrated form of the compound appears to exist in the temperature range of 200–280 °C. In this context, activation at 150 °C may not be sufficient to remove coordinated water molecules, and the material analyzed by powder X-ray diffraction (Figure S1) could still correspond to a partially hydrated phase. I know the authors have used the original method in the SU-101 paper, however, did the authors perform PXRD measurements after activation at 200 °C?

11) Additionally, was the activated sample exposed to air in order to evaluate possible rehydration effects? If available, PXRD data collected after activation at 200 °C could be included for comparison.

12) Furthermore, UV–vis and photoluminescence results, since the coordination environment and hydration state can significantly affect the electronic structure and emission properties. Could the authors clarify whether it is possible to perform photoluminescence measurements in the solid state for the SU-101 compound, instead of in suspension, in order to avoid surface alterations and interactions with the solvent (cyclohexane)?

13) I recommend including in the main manuscript text the formula Bi2O(H2O)2(C14H2O8)·nH2O (n = 2) of the compound, as well as its dehydrated form. This will serve only as a reference and for greater clarity. Furthermore, the term "Thermogravimetric Analyzer" should be written as "Thermogravimetric Analysis," and the TG heating rate should also be added to the text.

Then, considering the scientific work as a whole, as well as the information and methods employed, I believe it could be published in Nature Communications after a minor revision.

Reviewer #4

(Remarks to the Author)
This study investigates the hydration-dependent exciton-phonon coupling in SU-101, a metal-organic framework (MOF) photocatalyst, and focuses on the under-explored correlation between solvent coordination and exciton-phonon interactions in the optoelectronic properties of MOFs. It presents somehow innovations in the fundamental research of MOF photocatalysis, yet suffers from several limitations in methodology and research system design. In comparison with relevant studies in the field, obvious shortcomings are also observed in terms of research scope and the depth of experimental validation. The detailed analysis I offer below is presented from three aspects: innovations, inherent limitations, and shortcomings relative to other related works.
Firstly, I'd like to express the the thanks to the contribution. This work fills the research gap in the regulation of exciton-phonon coupling by coordinated solvents in the optoelectronic properties of MOFs, and stands as one of the first systematic studies to reveal the correlation between hydration states and exciton-phonon interactions in MOFs. Its innovations are mainly included (1) First identification of the regulatory effect of coordinated water on exciton-phonon coupling in MOFs, and discovery of hydration-dependent exciton localization effect (2) Expanding the exciton-phonon coupling research system from inorganic/organic semiconductors to hybrid MOF materials, and improving the exciton physics of low-dimensional/hybrid materials (3) Integrating ab initio theory with precise experimental validation to establish a multi-scale research methodology for MOF optoelectronic properties (4) Revealing a potential new dimension for regulating MOF photocatalytic performance: hydration state modulation (5) Clarifying the optoelectronic characteristics of MOFs with large pores and flexible ligands.

Secondly, despite the breakthroughs in core scientific issues, this study has obvious inherent limitations due to the constraints of research system, methodology, and the depth of experimental validation, mainly reflected in the following aspects:
(1) the study only focuses on SU-101 (a Bi-ellagate-based MOF) and does not verify other types of MOFs (e.g., Zn-, Ti-, Zr-based MOFs, or MOFs with different ligands/pore structures). It is thus impossible to determine whether the hydration-dependent exciton-phonon coupling is a unique property of SU-101 or a universal law for MOFs with large pores/flexible ligands, which restricts the applicability of the conclusions.
(2)Even thought the study discovers the regulatory effect of coordinated water on exciton-phonon coupling but fails to clarify its microscopic mechanism. For example, how the coordination bond between coordinated water and Bi sites (Bi-OH_2) affects phonon modes (e.g., aromatic ring vibration, Bi-O stretching); which specific phonon modes are the key to regulating exciton localization; and how the loss of coordinated water changes the π-π stacking and hydrogen bond network of ligands, thereby affecting exciton transport and localization. The mechanism analysis only stays at the correlation between "structural changes and property changes", without an in-depth exploration at the atomic/bonding microscopic level.
(3)Furthermore, the experiments verify the changes in optical properties calculated by theory through UV-Vis absorption and photoluminescence (PL) spectra, but do not correlate hydration states, exciton-phonon coupling with actual photocatalytic performance. For instance, the study does not test the activity differences of SU-101 with different hydration loadings in specific photocatalytic reactions (e.g., benzylamine coupling, pollutant degradation), nor verify the effects of exciton localization/delocalization on charge separation efficiency and photogenerated carrier lifetime. This results in a lack of direct experimental support for the application value of the research.

(4)In simulation section, several simplifications in theoretical simulation may cause deviations. It is mainly presented: (1) neglecting nonadiabatic effects and only treating exciton-phonon coupling based on the Born-Oppenheimer approximation; (2) assuming that the dielectric properties of displaced configurations are consistent with those of the static configuration to reduce computational costs; (3) only considering phonon behavior under the harmonic approximation—although the harmonicity of low/high-frequency modes is verified, the influence of possible anharmonic phonons on coupling in highly flexible MOFs is not evaluated. These simplifications may lead to deviations between the calculated results of exciton binding energy and band gap renormalization and the actual values. (4) the study only simulates the phonon effects at 0 K and 300 K, and does not explore the influences of low temperatures (e.g., 77 K) and medium-high temperatures (e.g., 400 K, 500 K) on the exciton-phonon coupling of hydrated/activated SU-101. It is thus impossible to clarify the synergistic regulation law of temperature and hydration state, and it is difficult to match the possible non-room temperature working conditions in actual photocatalytic processes. (5) only two types of samples are prepared in the experiments，fox example, hydrated state (as-made) and fully dehydrated state (activated), without achieving gradient regulation of hydration loading (e.g., intermediate states with different water contents). In addition, in-situ characterization techniques (e.g., in-situ XRD, in-situ FT-IR, in-situ XPS) are not used to real-time monitor the evolution of MOF structure and electronic properties during the change of hydration states, making it impossible to establish a quantitative relationship among "hydration loading, exciton-phonon coupling strength, and optical properties".

As a fundamental research on MOF optoelectronic properties, this study exhibits obvious shortcomings in research scope, methodological perfection, the integration depth of experiment and theory, and application orientation compared with relevant studies on MOF excitons, exciton-phonon coupling in organic/inorganic semiconductors, and MOF photocatalytic modification. The specific comparisons I proposed are as follows:
(1)Previous studies (10.1021/acs.jpclett.1c00543) have confirmed the existence of strongly bound excitons in MOFs, but most of these studies focus on the excitonic characteristics of MOFs in a single state. Although this study expands the regulatory dimension of hydration states, related works have realized the combination of in-situ spectroscopy and femtosecond transient absorption to investigate the exciton relaxation and charge transfer dynamics in MOFs. In contrast, this study only conducts steady-state UV-Vis and PL characterization, without involving the dynamic processes of excitons, making it impossible to clarify how phonons affect exciton lifetime and dissociation efficiency. In addition, some studies (e.g., Ph.D thesis: Probing Exciton Dynamics in Metal-Organic Frameworks; 10.1002/adfm.202522083; 10.1002/anie.202418017; 10.1039/D2SC02503E) have realized the comparison of multiple MOF systems and established a quantitative law between

“ligand conjugation degree/metal node type and exciton binding energy”. However, this study only focuses on SU-101 without system expansion, resulting in the universality of its conclusions being far lower than that of related studies.

Exciton-phonon coupling is a classic research direction in organic molecular crystals, halide perovskites, and two-dimensional semiconductors. Mature studies in this field have realized the inclusion of nonadiabatic effects, simulation of anharmonic phonons, and quantitative characterization of exciton-phonon coupling (e.g., coupling strength factor λ). In comparison, this study has the following shortcomings:
(1)It does not quantitatively calculate the exciton-phonon coupling strength, but only indirectly reflects the coupling effect through the degree of exciton localization and the amplitude of band gap renormalization. There is a lack of quantitative comparison with traditional semiconductors, making it impossible to clarify the strength level of exciton-phonon coupling in MOFs.
(2)It neglects nonadiabatic effects and anharmonic phonons, while studies on organic crystals and perovskites have confirmed that nonadiabatic effects and anharmonic phonons are crucial for exciton dissociation and transport. The simplified assumptions in this study make it unable to explain more complex exciton dynamic behaviors. Related studies (e.g., https://chemrxiv.org/engage/chemrxiv/article-details/67819b93fa469535b982b707; 10.1021/acs.nanolett.3c01936) have realized the precise regulation of single phonon modes and clarified the effect of specific phonon modes on excitons. However, this study only points out that aromatic ring vibration and Bi-O stretching are key phonon modes, without realizing the separation and regulation of single modes, leading to vague mechanism analysis.
(3)The core value of this study is to provide a new regulatory dimension for MOF photocatalysis, but this MS lacks the correlation with performance and the verification of actual application scenarios compared with applied studies on MOF photocatalytic modification.This work does not test any photocatalytic reaction activity and only stays at the characterization of optical properties, making it impossible to prove the improvement effect of hydration state regulation on actual catalytic performance. It is necessary to supplement the multi-index optimization of MOF photocatalysts (e.g., light absorption range, charge separation efficiency, cycle stability) and clarify the correlation between modification mechanism and catalytic performance combined with in-situ characterization.
In addition, this study only conducts offline hydration/activation treatment, without realizing in-situ regulation, nor evaluating the stability of hydration states (e.g., whether hydration states change during the catalytic process), resulting in a lack of feasibility analysis for practical applications.

This study only characterizes the structural changes after hydration/dehydration through XRD and FT-IR, without clarifying the specific coordination sites, binding modes, and dynamic behaviors of coordinated water in SU-101. It is suggested to supplement the combination of in-situ neutron scattering, in-situ NMR, and molecular simulation to accurately characterize the interaction sites, binding energy, and dynamic behaviors between solvents and MOFs, and establish a quantitative model of “hydration loading - structural parameters - optical properties” (refer to 10.1038/s41598-019-51344-0).

I hope the authors need to provide relevant experimental and theoretical data according to the above suggestion, After revisions, the manuscript is recommended to be resubmitted to other Nature Portfolio journals.

Version 1:

Reviewer comments:

Reviewer #1

(Remarks to the Author)
"I am satisfied with the revisions made by the authors. Accordingly, I recommend accepting the manuscript in its current form.

Reviewer #2

(Remarks to the Author)
The author has carefully revised the article in accordance with my suggestions. I recommend that the editor accept this article.

Reviewer #3

(Remarks to the Author)
Based on these review comments, the authors were able, within the available format, to address all the questions and considerations raised during the review process. Then, after evaluating the changes made to the manuscript and the revised version, I believe that the manuscript is ready for publication.

Reviewer #4

(Remarks to the Author)
none

Open Access This Peer Review File is licensed under a Creative Commons Attribution 4.0 International License, which permits use, sharing, adaptation, distribution and reproduction in any medium or format, as long as you give appropriate credit to the original author(s) and the source, provide a link to the Creative Commons license, and indicate if changes were made.

In cases where reviewers are anonymous, credit should be given to 'Anonymous Referee' and the source.

The images or other third party material in this Peer Review File are included in the article's Creative Commons license, unless indicated otherwise in a credit line to the material. If material is not included in the article's Creative Commons license and your intended use is not permitted by statutory regulation or exceeds the permitted use, you will need to obtain permission directly from the copyright holder.

To view a copy of this license, visit https://creativecommons.org/licenses/by/4.0/

RESPONSE TO THE REVIEWERS

Referee comments article Nature Communications - NCOMMS-26-009753:
Hydration-dependent exciton-phonon coupling in a metal-organic framework photocatalyst

---

REVIEWER 1

Recommendation: Revisions

Comments: This work derives the conclusion—via theoretical calculations—that phonon can induce exciton localization, with this effect being particularly pronounced in water-bound SU-101. This is a compelling finding, and research in this direction is indeed relatively scarce.

Reviewer Point P 1.1 — However, the guiding value of this work for experimental studies, the experimental phenomena it can explain, and the scientific problems behind technical bottlenecks it can address are core priorities. Please clarify the significance of this work, or contextualize it with experimental questions.
Other specific questions also need to be answered. In photocatalysis, excitons in catalysts are typically expected to be delocalized (to facilitate photogenerated charge separation for separate oxidation/reduction reactions). Yet this work shows that phonons—especially in water-bound MOF catalysts—drive exciton localization (with enhanced localization at elevated temperatures), which would seem to hinder photocatalytic redox processes. However, many photothermal processes in experiments actually boost photocatalytic performance. How can this contradiction be explained from the perspective of phonon-exciton interactions?

Reply: *We thank the reviewer for carefully assessing the manuscript and for their insightful comments. We have clarified the "contradiction" by adding the following to the manuscript:*

> *Strong phonon-driven exciton localization in water-bound SU-101 may influence photocatalysis in non-trivial ways. Photocatalysis reflects a balance of multiple processes across different timescales that may compete. Phonon effects, including those beyond the adiabatic limit, can be either detrimental or desired (e.g. ultrafast exciton dissociation[1]) for photocatalysis, and are oftentimes competing. Moreover, although exciton localization is often linked to non-radiative recombination, this pathway is not necessarily rate-limiting in carrier generation. Therefore, the overall impact of exciton–phonon coupling on photocatalysis remains system-dependent, and our results provide a first step toward its microscopic understanding.*

Furthermore, to better put the significance of this work in context, we have added:

> *Lastly, exciton-phonon coupling and its sensitivity to changes in the local chemical environment can influence multiple aspects of photocatalytic processes, including light absorption, and subsequent exciton dynamics and and dissociation into free charge carriers. Therefore, a comprehensive view on exciton-phonon coupling in materials with strong excitonic effects is essential for interpreting experimental observations and guiding photocatalyst design.*

Reviewer Point P 1.2 — Is the conclusion obtained here generalizable to MOF materials as a class?

Reply: *We have clarified the generalization of our conclusions to other MOFs in the last paragraph of the Discussion section:*

> *In SU-101, the impact of bound solvent arises indirectly through structural modifications, where solvent-derived hydrogen bonds modulate metal-metal distances and consequently the electronic structure of this MOF. Other MOFs with bound solvents may exhibit similar behavior, provided that the solvent induces comparable changes in the atomic geometry. More generally, we expect strong excitonic effects and exciton-phonon coupling to be common features of MOFs, particularly those with soft degrees of freedom associated with organic linkers and large pores.*

We have also added a sentence on how the Bi—Bi distances may play an important role:

> *The presence of Bi with its polarizable core and the degree of Bi—Bi spacing also affects the optoelectronic properties of SU-101, e.g. via contributions to dielectric screening.*

Reviewer Point P 1.3 — The experimental observation signal is not provided in Figure S1.

Reply: *We have updated the plotting style to clarify the difference between the observed and calculated signals in Figure S1.*

Reviewer Point P 1.4 — Theoretical calculations indicate that the band gap of SU-101 decreases upon dehydration—has this phenomenon been experimentally verified?

Reply: *Yes, and we have added a discussion regarding this in the Supporting Information:*

> *We have experimentally probed the effect of activation (dehydration) on the optical gap of SU-101 using UV-vis diffuse reflectance spectroscopy and Tauc analysis (Figure S16). Both our G_0W_0-BSE and experimental results show a consistent decrease in the apparent optical gap upon activation compared to the as-made material. This trend is reproducible across independently prepared samples and is in qualitative agreement with the theoretical prediction that dehydration reduces the gap.*

We now refer to this discussion in the manuscript:

> *We notice a slight decrease in the Tauc plot-derived optical gap upon water removal (Figure S16, see Supporting Information Section 7 for more details), and a consistent red shift in the emission spectra (Figure S13).*

Reviewer Point P 1.5 — For the model construction in Figure 1b, what is the basis for determining atomic coordinates, ligand connectivity, and water molecule positions? Why does water incorporation lead to lattice contraction along the a/b directions but expansion along the c direction?

Reply: *We clarified the model construction in Figure 1 with the following modification:*

> *View in ab plane, cell parameters, and view of rod-shaped metal node array along the c axis for b. water-bound SU-101, and c. activated SU-101, obtained from a previously reported structural model (CCDC 2003313[2]). Water-bound SU-101 is obtained by DFT-optimizing the structure after free solvent removal, whereas the activated SU-101 is obtained by DFT-optimizing the structure after removing all water molecules.*

Regarding the expansion along c direction, we have clarified:

> *The changes in lattice parameters are due to the loss of H-bonds between Bi-coordinated water and neighboring O atoms, which lead to shorter Bi—Bi distances in water-bound SU-101, and potentially improved π-π stacking (Figure 1).*

Reviewer Point P 1.6 — How is the shift of the first XRD peak (Figure 1a) attributed to c-axis expansion? It is recommended to label the Miller indices corresponding to each XRD peak to clarify this correlation.

Reply: *We have added a paragraph in the Supporting Information to clarify the assignment of the lattice parameter changes:*

> *The assignment of the lattice parameter changes (i.e., contraction in a and b, and expansion along c) is not based on the shift of a single diffraction peak, but rather on full-pattern analysis via Le Bail fit of the PXRD data. The first low-angle peak in Figure 1a corresponds to the (110) reflection. Its shift to higher 2θ upon activation indicates a reduction in the corresponding d-spacing, and therefore a contraction in the ab plane. As reported in Figure S1, the refined unit cell parameters show a decrease in a and b and a concomitant increase in c upon activation. The (110) reflection alone does not provide direct information about changes along the c-axis.*

As per the reviewer's recommendation, we have included Miller index labeling of the main reflection in Figure 1a and other indices in Figure S1 to improve clarity. To avoid any ambiguity, we modified the language in the manuscript as follows:

> *Upon removal of coordinated water, Le Bail refinement of the PXRD data in Figure S1 reveals a contraction along the a and b axes ($\approx 3\%$), and an expansion along the c axis ($\approx 3\%$). The shift of the low-angle (110) reflection to higher 2θ is consistent with a reduction in d-spacing within the ab plane, while the expansion along c is determined from the refined lattice parameters.*

Reviewer Point P 1.7 — What is the isosurface value used in Figures 2a and 2b?

Reply: *Thanks to the reviewer's comment, we have revisited the isosurface values utilized, and we chose new values to encompass a more standardized visual representation of the excitons. We have clarified the isosurface values in the legend of Figure 2, as below:*

> *Isosurfaces are shown enclosing 80% of the exciton probability density, with the corresponding isosurface values (water-bound: 10, activated: 558.5). Blue regions correspond to isosurface cross-sections.*

Reviewer Point P 1.8 — Why do phonons induce exciton localization in water-bound systems? Is this effect related to changes in the dielectric constant?

Reply: *This is an important question, and after investigating relevant atomic displacements and phonon modes in activated and water-bound SU-101, we added the following discussion to the manuscript:*

> *The localization arises from dynamic disorder — phonon-driven symmetry breaking associated with atomic displacements away from their equilibrium positions — rather than from changes in the dielectric properties which, in polar materials, can lead to exciton delocalization.[3]*

> *To understand the origin of the stronger phonon-induced exciton localization in water-bound SU-101, we analyze the 20 largest atomic displacements in the Monte Carlo-sampled configurations at 0 K and 300 K (Figure S10). In this analysis, larger displacements indicate a greater contribution of these atoms to the renormalization of the exciton energies. In the activated SU-101, C atoms involved in C=C bonds rank among the most displaced. In contrast, significant displacements of carbonyl O atoms are observed only in the water-bound structure. These observations suggest that hydration modifies the atomic degrees of freedom that couple to the exciton, leading to distinct exciton–phonon coupling mechanisms in the two cases.*

We have also added a section on the Supporting Information explaining how we did this analysis (Section 6.3).

We also analyzed the mode contribution to the overall phonon effects in both systems, modifying Figure 4 and adding the following text:

> *To clarify the mechanism behind the hydration-dependent electron-phonon coupling, we analyze the individual phonon mode contributions to the band gap renormalization in both systems (see Supporting Information Section 6.2). The phonon modes that contribute the most to the gap renormalization of activated SU-101 are the zero-point aromatic ring vibrations, such as the asymmetrical C=C stretching modes (≈ 1450 cm^{-1}, see Figure 4a-b, Figure S8) at 0 K; and a few lower frequency modes excited at 300 K (≈ 90 cm^{-1}, see Figure 4c), associated with a collective tilting of the ellagate linker and Bi-O stretching. For water-bound SU-101 (Figure 4d-e), the important phonon modes at 0 K (≈ 1480 cm^{-1}) are very similar to those of activated SU-101. However, at 300 K (Figure 4f), low-frequency modes associated with the rotation of the Bi-OH2 bond (≈ 200 cm^{-1}) also contribute for water-bound SU-101. These modes are not present in the activated MOF, for which a collective tilting of the linker is the major contributor at 300 K. Our findings demonstrate that different phonons impact excitonic properties at 300 K upon bound water removal, and explain the origin of the hydration-dependent phonon-induced exciton localization.*

Reviewer Point P 1.9 — How were the phonons in Figure 5 obtained?

Reply: *For clarity, we have redirected the reader in the legend of Figure 5 to the relevant section in the Supporting Information explaining how the absorption spectra with the inclusion of phonons is obtained:*

> *a. Ultraviolet-visible (UV-Vis) absorption spectra of the as-made (yellow) and activated (blue) materials of SU-101 measured under the same conditions, b. G_0W_0-BSE absorbance spectra of water-bound SU-101 and activated SU-101 (see Methods Section 4.4), without phonons (dashed lines) and with phonons (averaged over 10 displacements, see Supporting Information Section 13) at 0 K (dotted line) and 300 K (solid line).*

REVIEWER 2

Recommendation: Revisions

Comments: The authors present a paper on "Hydration-dependent exciton-phonon coupling in a metal-organic framework photocatalyst", the current manuscript needs improve before it should be considered for publication.

Reviewer Point P 2.1 — Thermodynamic stability of MOFs in reported applications should be evaluated and discussed.

Reply: *Indeed, thermodynamic stability in computational studies of MOFs can be important, in particular for in silico predicted MOF structures. However, here we focus on a MOF, SU-101, which is known to be stable,*[2] *a result validated by the experiments in our manuscript. Therefore, we can be confident in the thermodynamic stability of this material with regards to the scope of our manuscript.*

Reviewer Point P 2.2 — In the introduction, it should be more discussed detailed about the difference of MOFs with other metallic compounds for electron-phonon coupling,

Reply: *The reviewer makes a good point that it is important to explain what makes MOFs different from other classes of materials. We have elaborated further on this context:*

> *The strong excitonic effects and electron-phonon coupling in MOFs stem from electronic excitations localized on the organic linkers and are similar to those reported in organic solids, which can exhibit strong exciton-phonon coupling leading to localization.*[4] *As with organic solids and other molecular systems, displacements of atoms along high-frequency intramolecular coordinates drive this strong coupling,*[5] *and soft degrees of freedom give rise to the temperature dependence*[4] *and even self-trapping.*[6] *In inorganic semiconductors, Wannier-Mott excitons are delocalized over many unit cells and couple predominantly to polar optical phonon modes,*[3,7] *a mechanism that is essentially absent when the exciton is sub-unit-cell in extent in a less-polar MOF.*

Reviewer Point P 2.3 — Please explain more about the role of structure in electron-phonon coupling with a mechanism. What is the role of the ligand and metal?

Reply: *This is an important point, and we have added a paragraph on the contribution of specific atoms to the exciton-phonon coupling. Please see Point P 1.8.*

Reviewer Point P 2.4 — What are the advantages of choosing a certain MOF? Which metal is more suitable for which type of MOF?

Reply: *We agree with the reviewer that establishing design rules would be interesting, but this question is challenging to answer definitively without extensive additional studies beyond the scope of our work. We have elaborated on the matter:*

> *While extending our findings for other MOFs would be challenging due to the associated computational cost, we expect that phonons and excitons play an important role for MOF photocatalysts in general as they do here when the low-energy excitations are dominated by flexible organic linkers.*

Reviewer Point P 2.5 — The drawbacks and challenges are suggested also be discussed.

Reply: *We have highlighted the main challenge in extending our findings to other MOFs, as shown in Point P 2.4. Our considerations regarding neglecting anharmonicity and nonadiabatic effects are also stated, see Point P 4.8 and Point P 4.4.*

Reviewer Point P 2.6 — Some studies on stable MOFs can be addressed, such as Energy Environ. Sci., 2025,18, 7733.

Reply: *We are now addressing this study in the introduction:*

> *In MOFs, the metal sites are mostly coordinated by the surrounding organic linkers through reversible bonds, such as metal-carboxylate bonds, which influence the stability of these materials.*[8,9]

Reviewer Point P 2.7 — The individual contributions of each metal and ligand to the electron-phonon coupling should be discussed in greater detail.

Reply: *Please refer to Point P 1.8.*

REVIEWER 3

Recommendation: Revisions

Comments: In this manuscript "Hydration-dependent exciton-phonon coupling in a metal-organic framework photocatalyst", the authors present a study combining GW-BSE calculations, DFT structural analysis, and experimental UV-Vis photoluminescence measurements to investigate the role of hydration in modulating the excitonic and properties of SU-101 MOF. I belive that the work is impactful in the field of MOF light-harvesting and photocatalysis. The supplementary material is clear and provides additional experimental details, including data treated and processing procedures. However, the manuscript requires careful editing for consistency, and formatting before it can be considered end version for publication. Both "major" and minor conceptual and formatting issues are present. Then, considering the scientific work as a whole, as well as the information and methods employed, I believe it could be published in Nature Communications after a minor revision.

Reviewer Point P 3.1 — In the abstract and introduction sections, the sentence "ab initio GW and Bethe-Salpether equation" should be corrected to "GW approximation and Bethe–Salpeter equation (BSE)", and the spelling of "Salpether" should be corrected throughout the manuscript, please check it.

Reply: *We thank the reviewer for their careful assessment of the manuscript and insightful comments.*
We have fixed the typos, and the sentence in question now reads:

> *With the GW approximation and the Bethe–Salpeter equation (BSE) and treating exciton-phonon coupling with a finite difference method, we show that exciton-phonon interactions in SU-101 are strong and highly sensitive to the presence of bound water molecules.*

Reviewer Point P 3.2 — Statements such as "the latter in excellent agreement with our computational predictions" are overly strong and should be replaced with "in good agreement." as recommendation.
Sentences like "The excellent agreement with experimental trends. . . " overstate the results and should be moderated. Please avoid use of this type of structure.

Reply: *We have changed the relevant sections according to the reviewer's suggestions:*

> *Guided by our calculations, we synthesize SU-101 with different water loadings, and measure its photoluminescence emission and UV-Vis absorption spectra, the latter in* ***good*** *agreement with our computational predictions.*
>
> *The* ***good*** *agreement with experimental trends in optical absorption allows us to rationalize changes in the UV-Vis spectra upon bound solvent removal.*

Reviewer Point P 3.3 — In addition, some statements in the manuscript sounds "speculative". For example, the sentence "it is expected that excitons are also strongly bound" could be more accurately phrased as "Previous studies suggest relatively exciton binding energies in MOFs due to...".

Reply: *We have rephrased this sentence as suggested:*

> *For MOFs, due to their hybrid organic-inorganic nature and inherent porosity, studies report relatively large exciton binding energies*[10,11] *and significant phonon effects.*[12,13]

and

> *Although studies on exciton binding energies in MOFs are rare, MOFs can host strongly bound excitons due to their inherent porosity, low polarizability, and consequent weak dielectric screening.*[10,11]

Reviewer Point P 3.4 — Results section (page 4); the authors provide useful structural and functional context for SU-101 and highlights its relevance. However, the beginning of this discussion reads more like an extended background than an analysis of results. I recommend to revise this section to focus more clearly on presenting and interpreting the data.

Reply: *The reviewer raises a good point, and we have accommodated their suggestions by moving the extended background information to the Supporting Information, and elaborating on relevant structural information throughout the manuscript when needed:*

> *SU-101 is composed of ellagate organic linkers and rod-shaped metal nodes, with Bi being further coordinated by bound solvent (water). For more details on this MOF, we refer the reader to the Supporting Information.*
>
> *Upon removal of coordinated water, Le Bail refinement of the PXRD data in Figure S1 reveals a contraction along the* a *and* b *axes (*$\approx$ *3%), and an expansion along the* c *axis (*$\approx$ *3%). The shift of the low-angle (110) reflection to higher* 2θ *is consistent with a reduction in d-spacing within the ab plane, while the expansion along* c *is determined from the refined lattice parameters. The* c *axis is the direction in which SU-101's metal nodes arrange in "channels" in a rod-like shape. Therefore, activating SU-101 leads not only to an exposure of open metal sites,*[14] *but also to a contraction in the direction of the rod-shaped metal node.*

Reviewer Point P 3.5 — In addition, several formatting and consistency issues also need attention. For instance, energy units should be consistently reported as "eV" rather than "eVs", and dashes should be used correctly. Please check it in all manuscript.

Reply: *We have removed the occurrence of "eVs", the sentence now reads:*

> *In MOF-5, a commonly studied MOF with Zn clusters and 1,4-benzo dicarboxylic acid linkers, the exciton binding energy was reported to be substantial, on the order of a few eV.*[10]

We have also modified how we talk about numerical ranges, with one example below:

> *On the other hand, classic organic semiconductors tend to have more strongly-bound excitons with binding energies on the order of 0.5 eV to 1 eV,*[15] *(...).*

Reviewer Point P 3.6 — Againd, subjective phrases such as "Interestingly" or "proves crucial" should be avoided. Spelling nomenclature should also be consistent, for example "1,4-benzo dicarboxylic acid (Pag. 6)". Terminology for photoluminescence measurements should be standardized, using either "photoemission luminescence spectra" or "photoluminescence spectra" consistently. Check it.

Reply: *All occurrences of PL spectra were standardized to "photoluminescence emission spectra", an example highlighted below:*

> *To investigate whether these features can be seen experimentally, we measure both ultraviolet-visible (UV-Vis) absorption and photoluminescence emission spectra of water-bound and activated SU-101 (Figure 5a,Figure S13b).*

We rephrased the usage of "Interestingly" and "proves crucial" as highlighted below, and we have removed subjective statements as requested:

> *While we predict that both water-bound and activated SU-101 have similar strongly bound excitons at 300 K, the way they couple to phonons is hydration-dependent, with stronger exciton localization due to phonons for water-bound SU-101.*
>
> *Furthermore, our results indicate that accounting for phonons and excitons is important for interpreting the changes in the measured UV-Vis absorption spectra upon bound water removal.*

Reviewer Point P 3.7 — In the synthesis of SU-101, enter the percent yield of the compound. Why is the specific concentration value for the suspension in the UV-Vis absorption spectra approximately 1.24 µmol in 3 mL? The authors could add a comment for further clarification.

Reply: *We have updated the SU-101 synthesis description in the Experimental Methods section to specify the yield of the reaction.*

> *After the reaction, the MOF was recovered with a yield of 75 % by centrifugation and washed with water.*

And regarding the concentration value, we have elaborated further in the Methods section:

> *The concentration was chosen to ensure sufficient signal intensity while maintaining adequate light transmission through the suspension. We note that the spectral features are not sensitive to variations in concentration within this regime; the reported value (1.24 µmol in 3 mL) is provided for completeness and reproducibility.*

Reviewer Point P 3.8 — Regarding the experimental discussion, the authors report structural analysis after activation at 150 °C for 12 h under dynamic vacuum ("...The stability of the structure is also evaluated after activation at 150 °C for 12 hours, showing still a crystalline powder"). However, according to the thermogravimetric analysis (Supplementary Material, Figure S12), the release of coordinated water occurs close to 200 °C. The fully dehydrated form of the compound appears to exist in the temperature range of 200 °C to 280 °C. In this context, activation at 150 °C may not be sufficient to remove coordinated water molecules, and the material analyzed by powder X-ray diffraction (Figure S1) could still correspond to a partially hydrated phase. I know the authors have used the original method in the SU-101 paper, however, did the authors perform PXRD measurements after activation at 200 °C?

Reply: *To address this point, we performed additional activation of SU-101 at 200 °C under dynamic vacuum, without a prior solvent-exchange step, as suggested. Our new results have been incorporated in the Supporting Information:*

> *We performed additional PXRD measurements at 200 °C under dynamic vacuum, without a prior solvent-exchange step, to investigate whether increasing the temperature causes further changes in the PXRD patterns. PXRD measurements confirm that the framework remains crystalline after activation at 200 °C. However, its BET surface area is lower than that of solvent-exchanged*

> *samples, even if those were activated at 150 °C. This trend is consistent in both our measurements and the original report on SU-101, where solvent exchange, rather than activation temperature, was identified as the key step for achieving high accessible surface area. These results confirm that the structural and optical measurements after solvent exchange and activation at 150 °C are representative of the activated form of SU-101.*

Reviewer Point P 3.9 — Additionally, was the activated sample exposed to air in order to evaluate possible rehydration effects? If available, PXRD data collected after activation at 200 °C could be included for comparison.

Reply: *We thank the reviewer for this suggestion. In response, we performed additional PXRD measurements on samples activated at 150 °C and 200 °C (without solvent exchange), with data collected in ambient conditions. In both cases, the PXRD patterns of the MOF show the expected shift of the low-angle reflection. However, this shift is less pronounced after air exposure, which we attribute to partial rehydration of the framework under ambient conditions.*

Reviewer Point P 3.10 — Furthermore, UV–vis and photoluminescence results, since the coordination environment and hydration state can significantly affect the electronic structure and emission properties. Could the authors clarify whether it is possible to perform photoluminescence measurements in the solid state for the SU-101 compound, instead of in suspension, in order to avoid surface alterations and interactions with the solvent (cyclohexane)?

Reply: *We thank the reviewer for this suggestion. In response, we performed additional UV-vis and photoluminescence measurements in the solid state for SU-101, and we added our new results to the Supporting Information:*

> *We performed additional UV-vis and photoluminescence measurements in the solid state for SU-101, including (i) the as-synthesised material, (ii) samples activated at 150 °C, and (iii) samples activated at 200 °C (Figure S14,Figure S15). These activated samples were prepared without prior solvent exchange. We observe that the photoluminescence spectra in the solid state exhibit more structured features compared to the smoother emission profiles obtained in suspension. The difference is consistent with increased spectral broadening in suspension, where dynamic disorder and solvent interactions lead to the merging of individual transitions to form a continuous band. Importantly, the overall trends upon activation remain consistent between the two measurement approaches, supporting that the observed changes in optical properties are intrinsic to the material and not driven by solvent effects.*

Reviewer Point P 3.11 — I recommend including in the main manuscript text the formula Bi2O(H2O)2(C14H2O8)·nH2O (n = 2) of the compound, as well as its dehydrated form. This will serve only as a reference and for greater clarity. Furthermore, the term "Thermogravimetric Analyzer" should be written as "Thermogravimetric Analysis," and the TG heating rate should also be added to the text.

Reply: *We thank the reviewer for pointing out the typo, and we have corrected it to "Thermogravimetric Analysis".*

We have added the heating rate to the Methods section:

> ***Thermogravimetric Analysis (TGA)*** *The PerkinElmer TG instrument was used for TGA measurement. The measurement was performed under air flow from room temperature up to 700 °C, starting at 30 °C, with a heating rate of 5 K/min.*

Lastly, we have included the chemical formulas for greater clarity, as suggested:

> *To evaluate this effect further, we perform GW-BSE calculations on the system with (water-bound/hydrated,* $Bi_2O(H_2O)_2(C_{14}H_2O_8)$*) and without (activated,* $Bi_2O(C_{14}H_2O_8)$*) coordinated water molecules at Bi sites.*

REVIEWER 4

Recommendation: Revisions

Comments: This study investigates the hydration-dependent exciton-phonon coupling in SU-101, a metal-organic framework (MOF) photocatalyst, and focuses on the under-explored correlation between solvent coordination and exciton-phonon interactions in the optoelectronic properties of MOFs. It presents somehow innovations in the fundamental research of MOF photocatalysis, yet suffers from several limitations in methodology and research system design. In comparison with relevant studies in the field, obvious shortcomings are also observed in terms of research scope and the depth of experimental validation. The detailed analysis I offer below is presented from three aspects: innovations, inherent limitations, and shortcomings relative to other related works. Firstly, I'd like to express the the thanks to the contribution. This work fills the research gap in the regulation of exciton-phonon coupling by coordinated solvents in the optoelectronic properties of MOFs, and stands as one of the first systematic studies to reveal the correlation between hydration states and exciton-phonon interactions in MOFs. Its innovations are mainly included (1) First identification of the regulatory effect of coordinated water on exciton-phonon coupling in MOFs, and discovery of hydration-dependent exciton localization effect (2) Expanding the exciton-phonon coupling research system from inorganic/organic semiconductors to hybrid MOF materials, and improving the exciton physics of low-dimensional/hybrid materials (3) Integrating ab initio theory with precise experimental validation to establish a multi-scale research methodology for MOF optoelectronic properties (4) Revealing a potential new dimension for regulating MOF photocatalytic performance: hydration state modulation (5) Clarifying the optoelectronic characteristics of MOFs with large pores and flexible ligands. I hope the authors need to provide relevant experimental and theoretical data according to the above suggestion, After revisions, the manuscript is recommended to be resubmitted to other Nature Portfolio journals.

Reviewer Point P 4.1 — Secondly, despite the breakthroughs in core scientific issues, this study has obvious inherent limitations due to the constraints of research system, methodology, and the depth of experimental validation, mainly reflected in the following aspects: the study only focuses on SU-101 (a Bi-ellagate-based MOF) and does not verify other types of MOFs (e.g., Zn-, Ti-, Zr-based MOFs, or MOFs with different ligands/pore structures). It is thus impossible to determine whether the hydration-dependent exciton-phonon coupling is a unique property of SU-101 or a universal law for MOFs with large pores/flexible ligands, which restricts the applicability of the conclusions.

Reply: *We thank the reviewer for their detailed analysis of the manuscript. We have elaborated on the implications of our findings for experimental observations and for other systems in the Discussion. Please refer to Points P 1.1 and P 1.2.*
We emphasize the substantial computational cost of these calculations and note that, while not exhaustive, this work represents an important first step.

Reviewer Point P 4.2 — Even thought the study discovers the regulatory effect of coordinated water on exciton-phonon coupling but fails to clarify its microscopic mechanism. For example, how the coordination bond between coordinated water and Bi sites (Bi-OH_2) affects phonon modes (e.g., aromatic ring vibration, Bi-O stretching); which specific phonon modes are the key to regulating exciton localization; and how the loss of coordinated water changes the π-π stacking and hydrogen bond network

of ligands, thereby affecting exciton transport and localization. The mechanism analysis only stays at the correlation between "structural changes and property changes", without an in-depth exploration at the atomic/bonding microscopic level.

Reply: *We have further elaborated on the different mechanisms for exciton-phonon coupling for water-bound and activated SU-101, as well as the different phonon modes contributing to the overall phonon effect at* 0 K *and* 300 K*. Please refer to Point P 1.8.*

Reviewer Point P 4.3 — Furthermore, the experiments verify the changes in optical properties calculated by theory through UV-Vis absorption and photoluminescence (PL) spectra, but do not correlate hydration states, exciton-phonon coupling with actual photocatalytic performance. For instance, the study does not test the activity differences of SU-101 with different hydration loadings in specific photocatalytic reactions (e.g., benzylamine coupling, pollutant degradation), nor verify the effects of exciton localization/delocalization on charge separation efficiency and photogenerated carrier lifetime. This results in a lack of direct experimental support for the application value of the research.

Reply: *Regarding photocatalytic performance, we have added a short discussion in the manuscript. Please see Point P 1.1.*
We have added new results on the photogenerated carrier lifetime, please see Point P 4.7.
Lastly, the impact of exciton-phonon coupling and exciton localization on different steps of photocatalysis can be nontrivial and system-dependent. In the case of charge separation efficiency, exciton localization may promote charge recombination, but it can also enhance charge separation, for example in the formation of charge-transfer excitons. Therefore, we refrain from making general statements.

Reviewer Point P 4.4 — In simulation section, several simplifications in theoretical simulation may cause deviations. It is mainly presented: neglecting nonadiabatic effects and only treating exciton-phonon coupling based on the Born-Oppenheimer approximation; assuming that the dielectric properties of displaced configurations are consistent with those of the static configuration to reduce computational costs; only considering phonon behavior under the harmonic approximation—although the harmonicity of low/high-frequency modes is verified, the influence of possible anharmonic phonons on coupling in highly flexible MOFs is not evaluated. These simplifications may lead to deviations between the calculated results of exciton binding energy and band gap renormalization and the actual values.

Reply: *We agree with the reviewer that these are approximations taken into account in order to make our calculations tractable. However, we clearly and explicitly state this in the text, where we have now added a comment on the flexibility of the linkers:*

> *Nonadiabatic effects are neglected here, an acceptable approximation for organic linkers and in the context of steady-state measurements.*[4,16] *As shown in Figure S7, the harmonic approximation is justified by the good agreement between the harmonic and anharmonic potential energy surfaces for the optical modes with frequencies of* $18.8\,\mathrm{cm}^{-1}$ *and* $3071.2\,\mathrm{cm}^{-1}$ *(without SOC), and* $18.8\,\mathrm{cm}^{-1}$ *(with SOC). Such agreement can be attributed to the aromatic and polycyclic nature of the linkers in SU-101, when compared to linkers with softer degrees of freedom, such as succinic acid.*
>
> *For the displaced configurations, we reduce the computational cost by assuming that the change in the dielectric properties is minimal from the static (no-phonon) case to the displaced cases (see Supporting Information Section 11.4). In organic systems, the majority of the phonons are non-polar*[17] *and do not contribute to the static dielectric constant. Assuming a similar behavior for our material, we use the dielectric function from the static (no-phonon) structure (in Supporting Information Section 11.4, we test this approximation for a couple of displaced structures to validate our approach).*

Reviewer Point P 4.5 — the study only simulates the phonon effects at 0 K and 300 K, and does not explore the influences of low temperatures (e.g., 77 K) and medium-high temperatures (e.g., 400 K, 500 K) on the exciton-phonon coupling of hydrated/activated SU-101. It is thus impossible to clarify the synergistic regulation law of temperature and hydration state, and it is difficult to match the possible non-room temperature working conditions in actual photocatalytic processes.

Reply: *We have elaborated on the topic. We emphasize that these are non-trivial and expensive calculations that cannot be done for many temperatures.*

> *We evaluate phonon effects at these temperatures to disentangle the relative contributions of quantum nuclear motion and finite-temperature effects, particularly at room temperature which most closely reflects typical experimental conditions.*

We have also added on the importance of zero-point motion when compared to finite-temperature effects for SU-101:

> *Altogether, the vibrational renormalization of SU-101's DFT-PBE gap under different levels of hydration is dominated by zero-point effects.*

Reviewer Point P 4.6 — only two types of samples are prepared in the experiment, fox example, hydrated state (as-made) and fully dehydrated state (activated), without achieving gradient regulation of hydration loading (e.g., intermediate states with different water contents). In addition, in-situ characterization techniques (e.g., in-situ XRD, in-situ FT-IR, in-situ XPS) are not used to real-time monitor the evolution of MOF structure and electronic properties during the change of hydration states, making it impossible to establish a quantitative relationship among "hydration loading, exciton-phonon coupling strength, and optical properties".

Reply: *We have elaborated on the different levels of hydration:*

> *Partial hydration would likely result in structural changes between those observed for the bound water and fully activated states.*

Moreover, we have added a comment on in-situ studies, hopefully clarifying the scope of our work:

> *Although in-situ studies could capture real-time hydration effects, our first-principles analysis already reveals the main trends in exciton-phonon coupling and its hydration-dependency in SU-101.*

Reviewer Point P 4.7 — As a fundamental research on MOF optoelectronic properties, this study exhibits obvious shortcomings in research scope, methodological perfection, the integration depth of experiment and theory, and application orientation compared with relevant studies on MOF excitons, exciton-phonon coupling in organic/inorganic semiconductors, and MOF photocatalytic modification. The specific comparisons I proposed are as follows: Previous studies (10.1021/acs.jpclett.1c00543) have confirmed the existence of strongly bound excitons in MOFs, but most of these studies focus on the excitonic characteristics of MOFs in a single state. Although this study expands the regulatory dimension of hydration states, related works have realized the combination of in-situ spectroscopy and femtosecond transient absorption to investigate the exciton relaxation and charge transfer dynamics in MOFs. In contrast, this study only conducts steady-state UV-Vis and PL characterization, without involving the dynamic processes of excitons, making it impossible to clarify how phonons affect exciton lifetime and dissociation efficiency. In addition, some studies (e.g., Ph.D thesis: Probing Exciton Dynamics in Metal-Organic Frameworks;

10.1002/adfm.202522083; 10.1002/anie.202418017; 10.1039/D2SC02503E) have realized the comparison of multiple MOF systems and established a quantitative law between "ligand conjugation degree/metal node type and exciton binding energy". However, this study only focuses on SU-101 without system expansion, resulting in the universality of its conclusions being far lower than that of related studies.

Reply: *We thank the reviewer for their detailed and thoughtful assessment. We agree that advanced spectroscopic approaches, such as femtosecond transient absorption, provide powerful insights into exciton dynamics and charge transfer processes in MOFs. However, the primary objective of the present work is different: rather than establishing a comprehensive picture of the exciton relaxation dynamics, we focus on how the coordination environment (specifically hydration state) of the MOF modulates its electronic structure and steady-state optical response.*
In response to the reviewer's point, we have performed additional time-resolved photoluminescence measurements. We have incorporated the new results in the manuscript:

> *Lastly, we have performed time-resolved photoluminescence measurements. The average PL lifetime increases from* 2.496 ns *in the water-bound material to* 2.849 ns *after activation, as shown in Figure S17. This change suggests a minor modification of the overall excited-state decay rate upon dehydration. As such, these results are consistent with changes in the local coordination environment affecting exciton-phonon coupling and potential relaxation pathways.*

However, we refrain from making strong claims regarding exciton dynamics, exciton-phonon coupling strength, or charge separation processes based on this data alone. A rigorous treatment of these effects would require ultrafast spectroscopic techniques, which are beyond the scope of the present study.

Regarding the generality of the conclusions, we acknowledge that broader trends across MOF families have been established in prior work. In contrast, our study focuses on isolating the effect of hydration state within a single, well-defined system, allowing us to directly link changes in the coordination environment to modifications in the electronic structure and optical response. As hydration is a common feature among MOFs, we believe that this represents a broadly relevant and experimentally accessible approach to tuning optoelectronic behavior that will inspire future works. Even though we have not explicitly demonstrated this effect in additional MOFs, the details of how it arises in SU-101 clarify general conditions for observing these effects: low-energy excitations predominantly arising from organic linkers and appreciable changes in structure associated with hydration.

Reviewer Point P 4.8 — Exciton-phonon coupling is a classic research direction in organic molecular crystals, halide perovskites, and two-dimensional semiconductors. Mature studies in this field have realized the inclusion of nonadiabatic effects, simulation of anharmonic phonons, and quantitative characterization of exciton-phonon coupling (e.g., coupling strength factor λ). In comparison, this study has the following shortcomings: It does not quantitatively calculate the exciton-phonon coupling strength, but only indirectly reflects the coupling effect through the degree of exciton localization and the amplitude of band gap renormalization. There is a lack of quantitative comparison with traditional semiconductors, making it impossible to clarify the strength level of exciton-phonon coupling in MOFs.

Reply: *Exciton-phonon coupling in, e.g., halide perovskites and organic crystals is very different due to having Wannier-Mott vs Frenkel-like excitons, which call for different methodologies. Non-adiabaticity is indeed important in the latter but typically not the former, which better resembles our systems. This is highlighted in the main text as:*

> *Nonadiabatic effects are neglected here, an acceptable approximation for organic linkers.*[4,16]

Moreover, on anharmonicity, we have stated:

> *We test for possible deviations from the harmonic approximation by performing explicit displacements along a low-frequency and a high-frequency phonon mode, computing the total energy, and comparing the actual potential energy surface behavior with that of a perfectly harmonic system with the same frequency through fitting. According to Figure S7, even at larger displacements, the harmonic approximation seems to hold.*

Lastly, as an indication of the strength of electron-phonon coupling, we have performed mode-resolved electron-phonon coupling calculations that we discuss in Supporting Information Section 6.2 and in the main text as highlighted in Point P 1.8.

Reviewer Point P 4.9 — It neglects nonadiabatic effects and anharmonic phonons, while studies on organic crystals and perovskites have confirmed that nonadiabatic effects and anharmonic phonons are crucial for exciton dissociation and transport. The simplified assumptions in this study make it unable to explain more complex exciton dynamic behaviors. Related studies (e.g., `https://chemrxiv.org/engage/chemrxiv/article-details/67819b93fa469535b982b707`; doi:10.1021/acs.nanolett.3c01936) have realized the precise regulation of single phonon modes and clarified the effect of specific phonon modes on excitons. However, this study only points out that aromatic ring vibration and Bi-O stretching are key phonon modes, without realizing the separation and regulation of single modes, leading to vague mechanism analysis.

Reply: *Regarding the effect of specific phonon modes, please see Point P 1.8. Regarding nonadiabatic effects and anharmonic phonons, please see Point P 4.8.*

Reviewer Point P 4.10 — The core value of this study is to provide a new regulatory dimension for MOF photocatalysis, but this MS lacks the correlation with performance and the verification of actual application scenarios compared with applied studies on MOF photocatalytic modification.This work does not test any photocatalytic reaction activity and only stays at the characterization of optical properties, making it impossible to prove the improvement effect of hydration state regulation on actual catalytic performance. It is necessary to supplement the multi-index optimization of MOF photocatalysts (e.g., light absorption range, charge separation efficiency, cycle stability) and clarify the correlation between modification mechanism and catalytic performance combined with in-situ characterization.

Reply: *Please see Point P 1.1.*

Reviewer Point P 4.11 — In addition, this study only conducts offline hydration/activation treatment, without realizing in-situ regulation, nor evaluating the stability of hydration states (e.g., whether hydration states change during the catalytic process), resulting in a lack of feasibility analysis for practical applications.

Reply: *Please see Point P 4.6.*

Reviewer Point P 4.12 — This study only characterizes the structural changes after hydration/dehydration through XRD and FT-IR, without clarifying the specific coordination sites, binding modes, and dynamic behaviors of coordinated water in SU-101. It is suggested to supplement the combination of in-situ neutron scattering, in-situ NMR, and molecular simulation to accurately characterize the interaction sites, binding energy, and dynamic behaviors between solvents and MOFs, and establish a quantitative model of “hydration loading - structural parameters - optical properties” (refer to doi:10.1038/s41598-019-51344-0).

Reply: *Please see Point P 4.6.*

REFERENCES

[1] Gant, S. E.; Alvertis, A. M.; Coveney, C. J. N.; Haber, J. B.; Filip, M. R.; Neaton, J. B. Ultrafast spontaneous exciton dissociation via phonon emission in BiVO 4. *Physical Review Research* **2026**, *8*, DOI: doi:10.1103/xm7z-v1ks.

[2] Grape, E. S.; Flores, J. G.; Hidalgo, T.; Martínez-Ahumada, E.; Gutiérrez-Alejandre, A.; Hautier, A.; Williams, D. R.; O'Keeffe, M.; Öhrström, L.; Willhammar, T.; Horcajada, P.; Ibarra, I. A.; Inge, A. K. A Robust and Biocompatible Bismuth Ellagate MOF Synthesized Under Green Ambient Conditions. *Journal of the American Chemical Society* **2020**, *142*, 16795–16804, DOI: doi:10.1021/jacs.0c07525.

[3] Alvertis, A. M.; Haber, J. B.; Li, Z.; Coveney, C. J. N.; Louie, S. G.; Filip, M. R.; Neaton, J. B. Phonon screening and dissociation of excitons at finite temperatures from first principles. *Proceedings of the National Academy of Sciences* **2024**, *121*, DOI: doi:10.1073/pnas.2403434121.

[4] Alvertis, A. M.; Haber, J. B.; Engel, E. A.; Sharifzadeh, S.; Neaton, J. B. Phonon-Induced Localization of Excitons in Molecular Crystals from First Principles. *Physical Review Letters* **2023**, *130*, DOI: doi:10.1103/physrevlett.130.086401.

[5] Alvertis, A. M.; Williams-Young, D. B.; Bruneval, F.; Neaton, J. B. Influence of Electronic Correlations on Electron–Phonon Interactions of Molecular Systems with the <i>GW</i> and Coupled Cluster Methods. *Journal of Chemical Theory and Computation* **2024**, *20*, 6175–6183, DOI: doi:10.1021/acs.jctc.4c00327.

[6] Feng, S.; Yang, Y.; Gao, L.; Watanabe, K.; Taniguchi, T.; Hu, Z.; Lu, J.; Ni, Z. Tunable self-trapped excitons in 2D layered rubrene. *Applied Physics Letters* **2021**, *118*, DOI: doi:10.1063/5.0049942.

[7] Filip, M. R.; Haber, J. B.; Neaton, J. B. Phonon Screening of Excitons in Semiconductors: Halide Perovskites and Beyond. *Physical Review Letters* **2021**, *127*, DOI: doi:10.1103/physrevlett.127.067401.

[8] Sanati, S.; Varma, R. S.; Liu, M.; Abazari, R. Non-noble metal–organic frameworks in electrocatalytic urea-assisted hydrogen production and energy-saving regeneration. *Energy & Environmental Science* **2025**, *18*, 7733–7755, DOI: doi:10.1039/d5ee02029h.

[9] Rieth, A. J.; Wright, A. M.; Dincă, M. Kinetic stability of metal–organic frameworks for corrosive and coordinating gas capture. *Nature Reviews Materials* **2019**, *4*, 708–725, DOI: doi:10.1038/s41578-019-0140-1.

[10] Kshirsagar, A. R.; Blase, X.; Attaccalite, C.; Poloni, R. Strongly Bound Excitons in Metal–Organic Framework MOF-5: A Many-Body Perturbation Theory Study. *The Journal of Physical Chemistry Letters* **2021**, *12*, 4045–4051, DOI: doi:10.1021/acs.jpclett.1c00543.

[11] Lee, J.-H.; Lee, S.-H.; Son, Y.-W. Benchmarking First-Principles Approaches for the Band Gap Prediction of Nanoporous Materials. *Journal of Chemical Theory and Computation* **2025**, *21*, 6922–6932, DOI: doi:10.1021/acs.jctc.5c00818.

[12] Chen, D.; Vankova, N.; Jha, G.; Yu, X.; Wang, Y.; Lin, L.; Kirschhöfer, F.; Greifenstein, R.; Redel, E.; Heine, T.; Wöll, C. Ultrastrong Electron-Phonon Coupling in Uranium-Organic Frameworks Leading to Inverse Luminescence Temperature Dependence. *Angewandte Chemie International Edition* **2024**, *63*, DOI: doi:10.1002/anie.202318559.

[13] Ma, W.; Song, X.; Yin, J.; Fei, H. Intrinsic self-trapped broadband emission from zinc halide-based metal–organic frameworks. *Chemical Communications* **2021**, *57*, 1396–1399, DOI: doi:10.1039/d0cc07320b.

[14] Obeso, J. L.; Gabriel Flores, J.; Flores, C. V.; Rios-Escobedo, R.; Aguilar-Pliego, J.; Ken Inge, A.; Antonio de los Reyes, J.; Peralta, R. A.; Ibarra, I. A.; Leyva, C. Lewis Acid-Catalyzed Ring-Opening Alcoholysis of Cyclohexene Oxide: The Role of Open Metal Sites in the Bi(III)-based Metal-Organic Framework SU-101. *ChemCatChem* **2023**, *15*, DOI: doi:10.1002/cctc.202300471.

[15] Rangel, T.; Berland, K.; Sharifzadeh, S.; Brown-Altvater, F.; Lee, K.; Hyldgaard, P.; Kronik, L.; Neaton, J. B. Structural and excited-state properties of oligoacene crystals from first principles. *Physical Review B* **2016**, *93*, DOI: doi:10.1103/physrevb.93.115206.

[16] Miglio, A.; Brousseau-Couture, V.; Godbout, E.; Antonius, G.; Chan, Y.-H.; Louie, S. G.; Côté, M.; Giantomassi, M.; Gonze, X. Predominance of non-adiabatic effects in zero-point renormalization of the electronic band gap. *npj Computational Materials* **2020**, *6*, DOI: doi:10.1038/s41524-020-00434-z.

[17] Fennie, C. J.; Rabe, K. M. Structural and dielectric properties of Sr2TiO4 from first principles. *Physical Review B* **2003**, *68*, DOI: doi:10.1103/physrevb.68.184111.
