# Supplementary material for: Hydration-dependent exciton-phonon coupling in a metal-organic framework photocatalyst
Source: Nat Commun. 2026 Aug 5;17:9423. doi: 10.1038/s41467-026-76097-z (PMC13538642; doi:10.1038/s41467-026-76097-z)
Supplement: Supplementary file 3 [file 41467_2026_76097_MOESM3_ESM.pdf]

Description of Additional Supplementary Files

Supplementary Movie 1

Description: Animated mode number 282, important for band gap renormalization of activated SU-101 at 0K and 300K (Fig. 3a)

Supplementary Movie 2

Description: Animated mode number 283, important for band gap renormalization of activated SU-101 at 0K and 300K (Fig. 3b)

Supplementary Movie 3

Description: Animated mode number 28, important for band gap renormalization of activated SU-101 at 300K (Fig. 3c)

Supplementary Movie 4

Description: Animated mode number 332, important for band gap renormalization of water-bound SU-101 at 0K and 300K (Fig. 3d)

Supplementary Movie 5

Description: Animated mode number 331, important for band gap renormalization of water-bound SU-101 at 0K and 300K (Fig. 3e)

Supplementary Movie 6

Description: Animated mode number 79, important for band gap renormalization of water-bound SU-101 at 300K (Fig. 3f)
