# Supplementary material for: Hydration-dependent exciton-phonon coupling in a metal-organic framework photocatalyst
Source: Nat Commun. 2026 Aug 5;17:9423. doi: 10.1038/s41467-026-76097-z (PMC13538642; doi:10.1038/s41467-026-76097-z)
Supplement: Supplementary file 4 [file 41467_2026_76097_MOESM10_ESM.docx]

*Supplementary Information Inventory*

The Supplementary Information provides additional details on structural properties, band structures, DFT-based photocatalytic descriptors, excitonic properties, absorption spectra, transient photoluminescence decays, and phonon effects. It also includes convergence tests and analyses supporting the approximations used throughout this work.
